# Substrate expansion of *Geotrichum candidum* alcohol dehydrogenase towards diaryl ketones by mutation

**DOI:** 10.1007/s00253-024-13375-0

**Published:** 2024-12-27

**Authors:** Zhongyao Tang, Yuuki Takagi, Afifa Ayu Koesoema, Tomoko Matsuda

**Affiliations:** Department of Life Science and Technology: Tokyo Kogyo Daigaku Seimei Rikogakuin Seimei Rikogakukei, Institute of Science Tokyo, 4259 Nagatsuta-Cho Midzeori-Ku, Yokohama, 226-8501 Japan

**Keywords:** Alcohol dehydrogenase, Asymmetric reduction, Diaryl ketone, Chiral diaryl alcohol, Site-directed mutagenesis, Enzyme engineering

## Abstract

**Abstract:**

Chiral diaryl alcohols, such as (4-chlorophenyl)(pyridin-2-yl)methanol, are important intermediates for pharmaceutical synthesis. However, using alcohol dehydrogenases (ADHs) in the asymmetric reduction of diaryl ketones to produce the corresponding alcohols is challenging due to steric hindrance in the substrate binding pockets of the enzymes. In this study, the steric hindrance of the ADH from *Geotrichum candidum* NBRC 4597 (*G. candidum* acetophenone reductase, *Gc*APRD) was eliminated by simultaneous site-directed mutagenesis of Phe56 (in the large pocket) and Trp288 (in the small pocket). As a result, two double mutants, Phe56Ile/Trp288Ala, and Phe56Ala/Trp288Ala, exhibited much higher specific activities towards 2-(4′-chlorobenzoyl)pyridine (4.5 μmol/min/mg and 3.4 μmol/min/mg, respectively) than the wild type (< 0.2 μmol/min/mg). In whole-cell-catalyzed asymmetric reductions of diaryl ketones, Phe56Ile/Trp288Ala significantly increased the isolated yields, which were over 90% for the reactions of most of the tested substrates. Regarding enantioselectivity, Phe56Ile/Trp288Ala and Phe56Ala/Trp288Ala, and Trp288Ala generally exhibited similar selectivity to produce (*R*)-alcohols with up to 97% *ee*.

**Key points:**

*• Phe56 in Geotrichum reductase (GcAPRD) was mutated to eliminate steric hindrance.*

*• Mutation at Phe56 increased enzymatic activity and expanded substrate specificity.*

*• Phe56Ile/Trp288Ala showed high activity and (R)-selectivity towards diaryl ketones.*

**Graphical Abstract:**

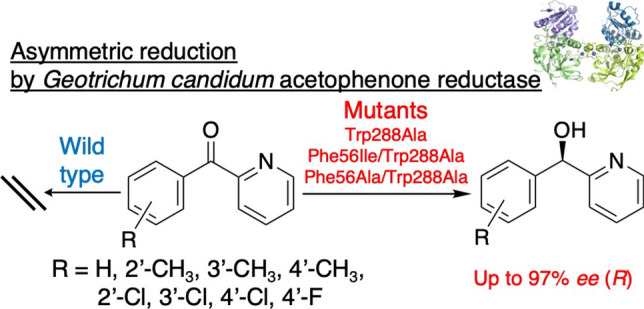

**Supplementary Information:**

The online version contains supplementary material available at 10.1007/s00253-024-13375-0.

## Introduction

Enantiopure alcohols, such as diaryl alcohols, are important intermediates for pharmaceutical synthesis. Among these alcohols, (*S*)-phenyl(pyridin-2-yl)methanol has analgesic and anticonvulsant properties (Li et al. 2021; Özdemir and Şahin 2024); (*S*)-(4-chlorophenyl)(pyridin-2-yl)methanol is an important intermediate for the synthesis of antihistamines, bepotastine (Chen et al. 2021; Li et al. 2021) and carbinoxamine (Xu et al. 2020; Li et al. 2021). Using alcohol dehydrogenases (ADHs), which belong to the large family of NAD(P)H-dependent oxidoreductases, in the asymmetric reduction of ketones is an environmentally friendly and straightforward method to produce enantiopure alcohols (Hou et al. 2020; Koesoema et al. 2020b; Hall 2021; Sardauna et al. 2023). Now biocatalytic asymmetric reduction of ketones by ADHs has attracted increasing attention as an alternative to chemical synthesis, which typically uses expensive metal catalysts or resolving agents. However, due to steric hindrance in substrate binding pockets of ADHs, their application in the reduction of diaryl ketones is often obstructed by low activity (Hou et al. 2020; Wu et al. 2020). The similarity in the size of the two aryl groups also makes the asymmetric reduction challenging, since they are difficult to be discriminated by the binding pockets (Wang et al. 2018; Xu et al. 2020). As a result, only a few ADHs have been demonstrated to catalyze the reduction of diaryl ketones, such as ADH from *Candida glabrata* (*Cg*ADH) (Sun et al. 2022), ADH from *Lactobacillus kefiri* (*Lk*ADH) (Wu et al. 2020, 2021), and ADH from *Kluyveromyces polyspora* (*Kp*ADH) (Xu et al. 2018, 2020; Zhang et al. 2022, 2024a). However, these ADHs belong to the short-chain dehydrogenase/reductase (SDR) family (Sun et al. 2022; Shanbhag 2023; Yuan et al. 2024). To the best of our present knowledge, using an ADH from medium-chain dehydrogenase/reductase (MDR) family to catalyze the reduction of diaryl ketones has been less studied, and the related studies mainly focus on ADH from *Thermoanaerobacter brockii* (*Tb*SADH) (Liu et al. 2019; Qu et al. 2019, 2022; Chen et al. 2021; Jiang et al. 2022). 

**Fig. 1:**
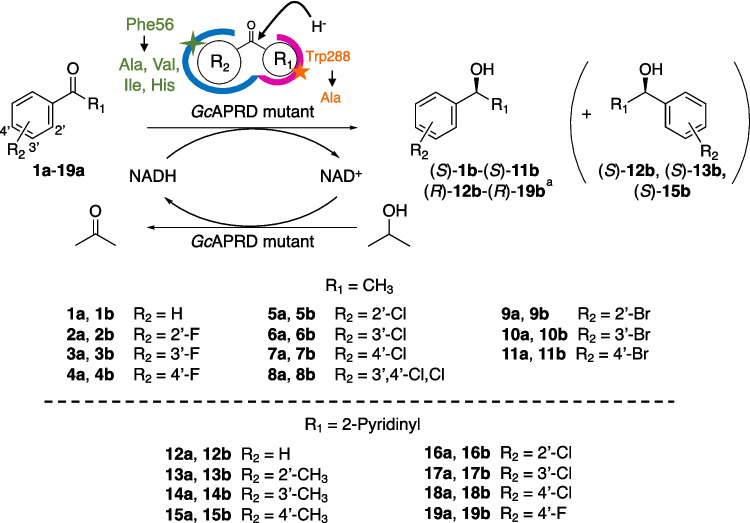
Mutants constructed and substrates/products used in this study. ^a^The absolute configuration (*S*/*R*) is determined by the Cahn–Ingold–Prelog priority rule. Therefore, when the phenyl group enters the large pocket, and the methyl or pyridyl group enters the small pocket, (*S*)-**1b**-(*S*)-**11b** and (*R*)-**12b**-(*R*)-**19b** are formed, respectively

To develop a variety of biocatalysts for the reduction of challenging diaryl ketones, further studies on enzyme mutagenesis are required to eliminate steric hindrance. An ADH from *Geotrichum candidum* NBRC 4597 (*G. candidum* acetophenone reductase, *Gc*APRD, PDB ID: 6ISV) belonging to the MDR family is a suitable target since it has been reported to present high thermostability (Nakata et al. 2010; Yamamoto et al. 2013; T.sriwong et al. 2020; T.sriwong et al. 2022) and non-aqueous solvent tolerance (Nakamura et al. 1999; Yamamoto et al. 2013). Furthermore, following Prelog’s rule, this enzyme exhibited excellent (*S*)-enantioselectivity in the asymmetric reduction of various ketones, consistent with observations from other Prelog ADH-catalyzed reductions (Prelog 1964; Koesoema et al. 2019a, b, 2020a, b, c; Shanbhag 2023; Zhang et al. 2024b), for example, *ee* > 99% (*S*) for the reduction of 3-hexanone (Koesoema et al. 2019b). *Gc*APRD has small and large pockets in the substrate binding site (Fig. 1), and steric hindrance in the small pocket can be eliminated by mutating Trp288 to Ala, Val, or other small amino acids (Koesoema et al. 2019a). Then, the Trp288 mutants exhibited expanded substrate specificity towards aliphatic ketones and altered enantioselectivity (Koesoema et al. 2019a, 2020b). For example, Trp288Val was able to catalyze the reduction of 4-octanone (propyl butyl ketone) (*ee* 87% (*R*)), which was not catalyzed by the wild type (Koesoema et al. 2019a, 2020b).

To investigate the reduction of diaryl ketones by *Gc*APRD, mutagenesis targeting the amino acids in the large pocket is necessary for expanding substrate specificity and improving activity. Based on the crystal structure of *Gc*APRD, the large pocket is composed of Ser47, His50, Ile51, Phe56, His66, Asn119, Leu122, and Leu264 (Koesoema et al. 2019b). Among these residues, Phe56 is located at the entrance of the large pocket. In this study, Phe56 was mutated to smaller amino acids based on preliminary docking simulation studies using diphenyl ketone as a substrate. First, four *Gc*APRD Phe56 single mutants (Phe56Ala, Phe56Val, Phe56Ile, and Phe56His) were constructed, considering the Van der Waals volumes and hydrophobicity of the amino acids, and were used for the reduction of acetophenone and its analogs (**1a**-**11a**) (Fig. 1). As a result, Phe56Ile demonstrated high activity and enantioselectivity (*ee* 99% (*S*)). Next, the double mutants Phe56Ile/Trp288Ala and Phe56Ala/Trp288Ala were constructed, and these double mutants and single mutants were used for the reduction of 2-benzoylpyridine analogs (**12a**-**19a**) (Fig. 1). As a result, improved activities towards diaryl ketones were observed, especially in the case of Phe56Ile/Trp288Ala. Regarding enantioselectivity, the two double mutants and Trp288Ala exhibited similar (*R*)-enantioselectivity to produce alcohols with up to 97% *ee*.

## Materials and methods

### Chemicals

Most chemicals are purchased from Nacalai Tesque (Kyoto, Japan), Wako (Osaka, Japan), Tokyo Chemical Industry (Tokyo, Japan), Bio-Rad (California, USA), and Sigma-Aldrich (St. Louis, USA). Primers for inverse PCR were obtained from Toyobo (Osaka, Japan) and Thermo Fisher Scientific (Tokyo, Japan). Substrates **1a**-**12a** and **18a** were purchased from Nacalai Tesque (Kyoto, Japan), while other substrates **13a**-**17a** and **19a** were synthesized by Grignard reactions following the literature method (Tao et al. 2012). Regarding racemic alcohol standards, *rac*-**1b** was purchased from Nacalai Tesque (Kyoto, Japan), while *rac*-**2b** and *rac*-**6b**-**11b** were previously prepared by our group (Koesoema et al. 2020a). Other racemic alcohol standards, *rac*-**3b**-**5b** and *rac*-**12b**-**19b**, were synthesized by sodium borohydride reduction of the corresponding ketones. Detailed information about the synthesis of **13a**-**17a**, **19a**, *rac*-**3b**-**5b**, and *rac*-**12b**-**19b** is provided in the Supplementary information (Sects. 1 and 2).

### Preparation of *Gc*APRD mutants by site-directed mutagenesis

*Gc*APRD wild type and Trp288Ala were prepared according to previously reported procedures (Yamamoto et al. 2013; Koesoema et al. 2019a). Using the pET-21b (+)-*Gc*APRD-His or pET-21b (+)-*Gc*APRD-His-Trp288Ala plasmid prepared in the previous study as templates, the plasmids for *Gc*APRD Phe56 mutants and double mutants were constructed by inverse PCR as reported previously (Koesoema et al. 2019a). The primer sequences are listed in Supplementary Table 1. Transformation of the resulting plasmids into *Escherichia coli* Rosetta™(DE3)pLysS (Novagen, USA), cultivation of the *E. coli*, overexpression of the enzyme using isopropyl β-d-1-thiogalactopyranoside (IPTG) induction, preparation of the cell-free extract, and enzyme purification were done as reported previously (Koesoema et al. 2019b). The protein concentration was measured by the Bradford method using bovine serum albumin (BSA) as the standard. A standard curve was made using different concentrations of BSA (0.125, 0.25, and 0.5 mg/mL). One milliliter of Bradford reagent was added to 20 μL of purified enzyme solution. The absorbance at 595 nm was measured after incubating the mixture at room temperature for 3 min. The SDS-PAGE of the mutants is shown in Supplementary Fig. 1.

### Enzymatic activity assays

All enzymatic assays were performed at 37 °C in triplicate by measuring the absorbance at 340 nm for 132 s using a UV–Vis spectrophotometer (UV-1900, Shimadzu, Japan) to determine the decrease in NADH concentration during ketone reduction. Activity was measured in 100 mM HEPES–NaOH buffer (pH 7.2, 1.0 mL) containing **1a**-**11a** (1.25 mM) or **12a**-**19a** (1 mM), NADH (0.3 mM), and purified enzyme (0.2–13.7 μg).

### Analytical-scale asymmetric reduction of 1a-11a by *Gc*APRD Phe56Ile

Analytical-scale reductions of **1a**-**11a** were performed in HEPES–NaOH buffer (100 mM pH 7.2, 3.0 mL) consisting of NAD^+^ (0.2 mM), 2-propanol (15% v/v), **1a**-**11a** (10 mM), and purified *Gc*APRD Phe56Ile (the amount of enzyme was calculated from the relative activity to achieve initial reaction rate of 3 µmol/min). Reductions were done at 30 °C with shaking at 200 rpm for 3 h. 270 μL of the reaction mixture was extracted with diethyl ether for conversion and enantioselectivity excess (*ee*) determination by chiral GC analysis with a flame ionization detector (GC-14B, Shimadzu, Japan) equipped with a chiral column (CP-Chirasil-Dex-CB, 0.32 mm × 0.25 μm × 50 m, Agilent, USA). The GC analysis conditions and retention times of the ketones, as well as *R* and *S* enantiomers, are listed in Supplementary Table 2.

### Preparative-scale asymmetric reduction of 5a by *Gc*APRD Phe56Ile

The preparative-scale reduction was performed in HEPES–NaOH buffer (100 mM pH 7.2, 50 mL) consisting of NAD^+^ (46 mg), 2-propanol (1.5 mL), **5a** (52.2 mg, 0.34 mmol), and purified *Gc*APRD Phe56Ile (15.17 mg/mL, 135 μL) at 30 °C with shaking at 200 rpm for 18 h. The product was extracted with diethyl ether three times, dried over MgSO_4_, and evaporated under reduced pressure. Silica gel column chromatography (hexane: ethyl acetate, 4:1) was performed to give (*S*)-**5b**. The product was characterized by ^1^H-NMR analysis, and *ee* was determined by chiral GC analysis. The absolute configuration was determined by comparing the optical rotation sign of the product with that reported in the literature. The ^1^H-NMR spectrum matched those reported in the literature (Liu et al. 2021). Detailed information is provided in the Supplementary information (Sect. 3).

### Asymmetric reduction of diaryl ketones by *Gc*APRD Trp288Ala, Phe56Ile/Trp288Ala, and Phe56Ala/Trp288Ala

Reductions of **12a**-**19a** were performed in HEPES–NaOH buffer (100 mM pH 7.2, 3.0 mL) consisting of 2-propanol (15% v/v for Trp288Ala and Phe56Ile/Trp288Ala or 5% v/v for Phe56Ala/Trp288Ala), a substrate (10.1–16.7 mM), and whole cells (0.5 g wet weight) at 30 °C with a shaking speed of 250 rpm for 3 h. The products were extracted with diethyl ether three times and evaporated under reduced pressure. Silica gel column chromatography (hexane: ethyl acetate, 2:1) was performed to give the corresponding products, **12b**-**19b**. The products were characterized by ^1^H-NMR analysis, and *ee* was determined by chiral HPLC analysis (LC-20AD with SPD-20A UV–Vis Detector, Shimadzu, Japan), equipped with a chiral column (CHIRALPAK^Ⓡ^ IA-3, 4.6 mm × 3 μm × 250 mm, Daicel, Japan). The HPLC analysis conditions and the retention times of the *R* and *S* enantiomers are listed in Supplementary Table 3. The ^1^H-NMR spectra were in agreement with those reported in the literature for **12b**-**16b** (Nian et al. 2019), **17b** (Liu et al. 2019), and **18b**-**19b** (Nian et al. 2019). Detailed information is provided in the Supplementary information (Sect. 4).

## Results

### Activity of *Gc*APRD Phe56 mutants towards acetophenone and its analogs

Phe56 in the large pocket in the *Gc*APRD substrate binding site was mutated to smaller amino acids, Ala, Val, Ile, and His, to eliminate the steric hindrance. Thus, single mutants (Phe56Ala, Phe56Val, Phe56Ile, and Phe56His) were constructed, overexpressed in *E. coli*, and purified, and their reduction activities towards acetophenone **1a** as a representative substrate was investigated. As listed in Table 1, among the tested enzymes, Phe56Ile exhibited the highest specific activity (48.8 μmol/min/mg) towards **1a**. Based on this result, Phe56Ile was selected as a promising candidate for the subsequent substrate specificity study.

**Table 1:** The specific activity of *Gc*APRD wild type and Phe56 mutants towards **1a**

Enzyme	Wild type^a^	Phe56Ala	Phe56Val	Phe56Ile	Phe56His
Specific activity towards **1a** (μmol/min/mg)	22.2	7.2	27.6	48.8	10.5

^a^The preparative-scale reduction of **1a** by the wild type was published previously, but not those for the relative activity

**Fig. 2:**
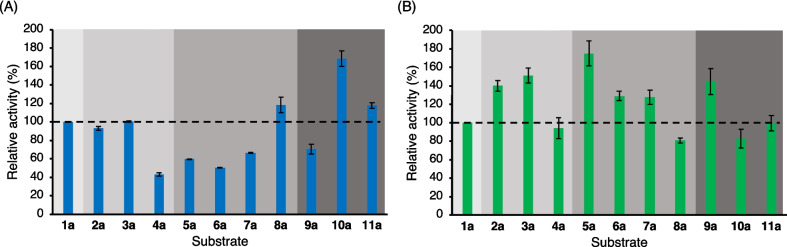
The relative activity of (**A**) wild type^a^ and (**B**) Phe56Ile towards **1a**-**11a**. ^a^The preparative-scale reduction of these substrates by the wild type were published previously, but not those for the relative activity

Then, the reduction activity of Phe56Ile towards **2a**-**11a** was investigated. The specific activity towards **1a** was set to 100% to calculate the relative activities towards **2a**-**11a** (Fig. 2). Phe56Ile exhibited improved activities on all the tested substrates compared to the wild type. Moreover, Phe56Ile exhibited a correlation between the position of the halogenated substituent and the activity; significantly increased activities of the 2′-chloro- or 2′-bromo-substituted compounds, **5a** and **9a**, compared to **1a** were observed (Supplementary Table 4).

### Asymmetric reduction of acetophenone and its analogs by Phe56Ile

**Fig. 3:**
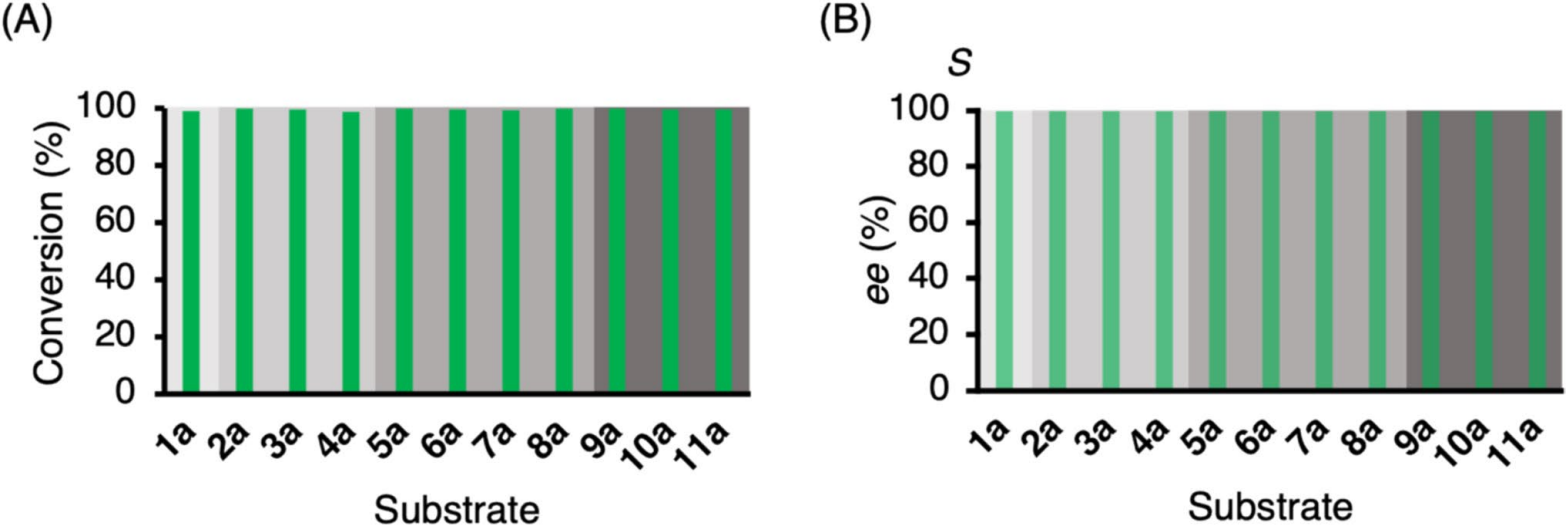
Analytical-scale reductions of **1a**-**11a** by *Gc*APRD Phe56Ile: (**A**) conversion and (**B**) enantioselectivity. The reaction conditions are described in the “Materials and methods,” section “Analytical-scale asymmetric reduction of 1a-11a by *Gc*APRD Phe56Ile”

Following the analysis of activity, asymmetric reductions of **1a**-**11a** on an analytical-scale by purified Phe56Ile were performed to confirm the conversion and enantioselectivity. 2-Propanol (15% v/v) was used as an auxiliary substrate for cofactor regeneration and as a cosolvent to facilitate the dissolution of ketones. The results are shown in Fig. 3. The reduction proceeded with more than 98% conversion for all tested substrates. Moreover, the enantioselectivities were > 99% *ee* (*S*) following Prelog’s rule (Prelog 1964), independent of the size and position of the halogenated substituents. To further investigate the versatility of Phe56Ile in organic synthesis, a preparative-scale reduction was conducted using **5a**, as the highest relative activity was obtained with **5a** among all tested substrates. The reaction resulted in > 99% conversion, 76.5% isolated yield, and > 99% *ee* (*S*).

### Activity of *Gc*APRD mutants towards diaryl ketones

With the excellent performance of Phe56Ile towards **1a**-**11a**, Phe56 in the large pocket and Trp288 in the small pocket were mutated to construct the double mutants, Phe56Ile/Trp288Ala and Phe56Ala/Trp288Ala. The two double mutants, as well as the wild type, Phe56Ile, Phe56Ala, and Trp288Ala were overexpressed in *E. coli*, purified, and the reduction activity towards 2-(4′-chlorobenzoyl)pyridine (**18a**) as a representative substrate was investigated. The specific activities are listed in Table 2. Compared to the wild type (< 0.2 μmol/min/mg), Trp288Ala increased the activity by more than sevenfold to 1.5 μmol/min/mg. Further mutagenesis in the large pocket to construct the double mutants, Phe56Ile/Trp288Ala and Phe56Ala/Trp288Ala, increased the specific activity more than 23-fold and 17-fold to 4.5 and 3.4 μmol/min/mg, respectively, although the Phe56 mutation alone did not show a significant change in activity.

**Table 2:** The specific activity of *Gc*APRD wild type and mutants towards **18a**

Enzyme	Wild type	Phe56Ile	Phe56Ala	Trp288Ala	Phe56Ile/Trp288Ala	Phe56Ala/Trp288Ala
Specific activity (μmol/min/mg)	< 0.2	< 0.1	< 0.1	1.5	4.5	3.4

### Asymmetric reduction of diaryl ketones by *Gc*APRD Trp288Ala, Phe56Ile/Trp288Ala, and Phe56Ala/Trp288Ala

**Fig. 4:**
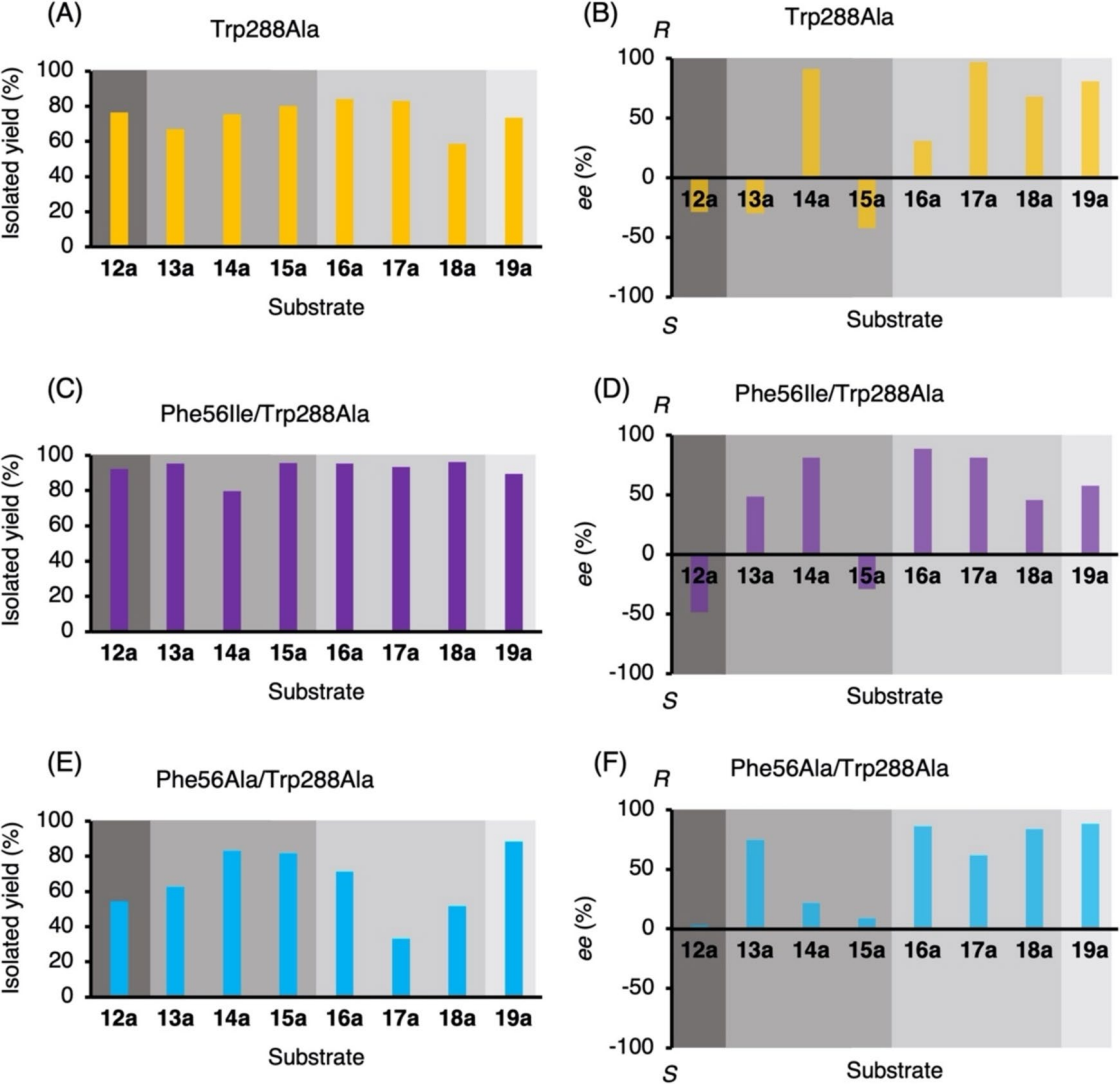
Reductions of **12a**–**19a** by *Gc*APRD mutants: (**A**) yield and (**B**) *ee* for the reaction by Trp288Ala, (**C**) yield and (**D**) *ee* for the reaction by Phe56Ile/Trp288Ala, and (**E**) yield and (**F**) *ee* for the reaction by Phe56Ala/Trp288Ala. The reaction conditions are described in the “Materials and methods,” section “Asymmetric reduction of diaryl ketones by *Gc*APRD Trp288Ala, Phe56Ile/Trp288Ala, and Phe56Ala/Trp288Ala”

Following activity analysis, asymmetric reductions of **12a**-**19a** by whole cells harboring Trp288Ala, Phe56Ile/Trp288Ala, or Phe56Ala/Trp288Ala were performed to confirm isolated yields and enantioselectivities. The reaction also used 2-propanol as an auxiliary substrate and cosolvent, similar to the single mutant cases. The results are shown in Fig. 4. Using Trp288Ala, isolated yields exceeding 80% were obtained for the reduction of **15a**-**17a**, while yields for the reactions of other substrates ranged from 58 to 80%. Compared to Trp288Ala, Phe56Ile/Trp288Ala significantly increased the yields for the reactions of most of the tested substrates. Specifically, the yields of **15b** and **18b** increased to 96%, and other products, except for **14b**, were obtained with yields above or around 90%. Phe56Ile/Trp288Ala not only improved activity (initial reaction rate) towards **18a** (Table 2) but also demonstrated high performance in the reduction reactions of 3 h. On the other hand, although Phe56Ala/Trp288Ala exhibited higher enzymatic activity towards **18a** than Trp288Ala (Table 2), higher isolated yields were not achieved, resulting in only up to 81% yield.

Regarding enantioselectivity, all three mutants generally produced (*R*)-alcohols following Prelog’s rule (Prelog 1964). For example, (*R*)-alcohols were produced by Trp288Ala in the reductions of *meta*-substituted **14a** and **17a** with *ee* of 91 and 97%, respectively. Phe56Ile/Trp288Ala also demonstrated high (*R*)-enantioselectivity for **14a** and **17a**, while the highest enantioselectivity of Phe56Ile/Trp288Ala was observed in the reduction of **16a**. The enantioselectivity for **16a** was also high when using the other double mutant, Phe56Ala/Trp288Ala. On the other hand, (*S*)-alcohols were produced from the reduction of **12a** and **15a** by Trp288Ala and Phe56Ile/Trp288Ala. When comparing Trp288Ala with Phe56Ile/Trp288Ala and Phe56Ala/Trp288Ala, significant improvements in enantioselectivity were observed for the reduction of 2′-substituted substrates **13a** and **16a**.

## Discussion

Based on the previous research on the reduction of ketones by ADHs, there are three factors affecting the activity, yield, and, enantioselectivity of ADH-catalyzed reactions: substrate binding pocket sizes (Qin et al. 2018; Koesoema et al. 2019a; Wu et al. 2020), hydrophobicity of amino acids in the binding pockets (Qin et al. 2018; Wu et al. 2021), and substituent properties of substrates (Qin et al. 2018; Koesoema et al. 2019a). These factors collectively determine the catalytic performance.

First, the sizes of the binding pockets of *Gc*APRD wild type and Trp288Ala were considered to determine their suitability for diaryl ketone reductions. In our previous study, the *Gc*APRD wild type exhibited excellent (*S*)-enantioselectivity for the reduction of halogenated acetophenones (Koesoema et al. 2019a), whereas Trp288Ala exhibited (*R*)-enantioselectivity (Koesoema et al. 2020a). This demonstrates that the small binding pocket of the wild type is suitable for a methyl group but too small for an aryl group (Koesoema et al. 2019a), whereas the enlarged small pocket in Trp288Ala is suitable for an aryl group (Koesoema et al. 2019a, 2020a), which provides a basis for Trp288Ala, but not the wild type, to exhibit catalytic activity toward diaryl ketones. As expected, Trp288Ala exhibited higher activity towards **18a** (1.5 μmol/min/mg) compared to the wild type (less than 0.2 μmol/min/mg) (Table 2).

Since the phenyl ring and the pyridine ring are similar in size, achieving high enantioselectivity is challenging (Wang et al. 2018; Xu et al. 2020). Nevertheless, depending on the substituent, *Gc*APRD mutants can reduce diaryl ketones with high enantioselectivity. For example, Trp288Ala exhibited high (*R*)-enantioselectivity for 3′-substituted substrates, yielding 91% *ee* (*R*) for **14b** and 97% *ee* (*R*) for **17b**. This observation suggests that the substituted phenyl group, rather than the pyridyl group, effectively enters the large pocket. This result aligns with the fact that 3′-substituted phenyl groups have a higher tendency to enter the large pocket than 2′- or 4′-substituted phenyl groups in the reduction of substituted acetophenones by Trp288Ala in previous studies (Koesoema et al. 2020a).

Further mutation of Phe56 at the entrance of the large pocket to smaller amino acids, creating mutants Phe56Ile/Trp288Ala and Phe56Ala/Trp288Ala, enlarged the large binding pocket by eliminating steric hindrance. Therefore, Phe56Ile/Trp288Ala exhibited the highest activity towards **18a** (4.5 μmol/min/mg) among all the tested enzymes. The higher activity of Phe56Ala/Trp288Ala towards **18a** (3.4 μmol/min/mg) than that of Trp288Ala is also attributed to the increased size of the large binding pocket. The literature search on mutations at position Phe56 or the associated loop region of MDR was conducted, and the alignment results of *Gc*APRD and several MDRs are shown in Supplementary Figs. 2 and 3. This position or the associated loop region has been mutated to design ADH-A mutant (Phe43Thr/Tyr54Gly/Leu119Tyr/Phe282Trp) to oxidize 1-phenylpropane-(1*R*,2*S*)-diol regioselectivity (Maurer et al. 2018), to design *cp*ADH5 (Cys57Val/Trp286Ser) to catalyze methyl 3-hexanoate (Ensari et al. 2018), and to design *Lactococcus lactis* alcohol dehydrogenase (LlAdhA) mutant (Tyr50Phe/Asn110Ser/Ile212Thr/Leu264Val) to reduce isobutyraldehyde to isobutanol (Liu et al. 2012) (Supplementary Fig. 3B). To the best of our knowledge, this position has not been mutated to expand the substrate specificity towards diaryl ketones. Previous studies only focused on another loop for the reduction of diaryl ketones to design *Tb*SADH mutants (Ala85Gly/Ile86Leu and Ala85Gly/Ile86Ala/Gln101Ala) (Liu et al. 2019; Qu et al. 2019, 2022; Chen et al. 2021; Jiang et al. 2022) (Supplementary Fig. 3B). These indicated that the loop in the large binding pocket was found to be targeted in this study to control the activity and enantioselectivity of MDR towards diaryl ketones.

Furthermore, the studies on ADH from *Thermoanaerobacter ethanolicus* (*Te*SADH, identical in sequence to *Tb*SADH) mutants mainly focused on mutations at position Trp110, which was mutated to Ala or Gly to enlarge the large binding pocket and enable the reduction of 2-tetralones that could not be reduced by the wild type (Musa et al. 2007; Bsharat et al. 2017). This strategy aligns with our approach of mutating Phe56 in *Gc*APRD to Ile or Ala to expand substrate specificity by enlarging the large binding pocket. Although Phe56 in *Gc*APRD and Trp110 in *Te*SADH are not related in the amino acid sequence, they occupy a similar space when the structures are compared (Supplementary Fig. 3C).

The mutation of Phe56 to Ile or Ala was especially effective in overcoming the limitation of the reaction of the 2′-substituted phenyl compounds. Phe56Ile exhibited especially higher activities towards 2′-chloro or 2′-bromo substituted compounds (**5a** and **9a**) than the wild type (Fig. 2, Supplementary Table 4). Moreover, Phe56Ile/Trp288Ala and Phe56Ala/Trp288Ala exhibited stricter (*R*)-enantioselectivity for 2′-substituted diaryl ketones (**13a** and **16a**) than Trp288Ala. For example, the *ee* of (*R*)-**16b** increased from 31% for Trp288Ala to 88% for Phe56Ile/Trp288Ala and 86% for Phe56Ala/Trp288Ala (Fig. 3). These results demonstrate that enlarging the large binding pocket can effectively eliminate the steric hindrance caused by the 2′-substituted phenyl group. Moreover, these results also demonstrated that matching the hydrophobicity of the substituent and the substrate binding site is important. As the halogen or methyl substitutes are strongly hydrophobic, the high hydrophobicity (Mozhaev et al. 1988; Bagchi 2013) of the large pocket in Phe56Ile is more favorable to 2′-chloro or 2′-bromo acetophenone and methyl or chloro-substituted 2-benzoylpyridines than that in the wild type. Importantly, these results may pave the way for solving the general problem in various biocatalysts of the steric hindrance caused by the 2′-substitution of the phenyl group at the α-position (Xu et al. 2018; Hoshino et al. 2020; Otsu et al. 2020; Wu et al. 2020, 2021; Zhang et al. 2022).

The electronegativity of the substituent on the substrates also affects the enantioselectivity of the reactions of diaryl ketones. When the substituent is located at the 2′- or 4′-position of the phenyl ring, there is an increasing tendency of (*R*)-enantioselectivity as the electronegativity of the substituent increases. For example, in the cases of 4′-substituted substrates, **15a** has an electron-donating methyl group, while **18a** and **19a** contain an electron-withdrawing chloro or fluoro group. The enantioselectivity for these substrates were reversed from *S* (or close to *racemic*) to *R* with increasing electronegativity. Trp288Ala showed 42% *ee* (*S*) for **15b** but achieved 68% *ee* (*R*) for **18b** and 81% *ee* (*R*) for **19b**. Phe56Ile/Trp288Ala exhibited a similar trend, with 29% *ee* (*S*) for **15b**, 46% *ee* (*R*) for **18b,** and 58% *ee* (*R*) for **19b**. For Phe56Ala/Trp288Ala, the enantioselectivities were 9% *ee* (*R*) for **15b**, 84% *ee* (*R*) for **18b**, and 88% *ee* (*R*) for **19b**. For 2′-substituted substrates, when the substituent was changed from methyl group (**13a**) to chloro group (**16a**), the (*R*)-enantioselectivities were increased. For Phe56Ile/Trp288Ala, *ee* (*R*) increased from 48% (**13b**) to 88% (**16b**). For Phe56Ala/Trp288Ala, *ee* (*R*) increased from 75% (**13b**) to 86% (**16b**). Notably, for Trp288Ala, *ee* reversed from 30% (*S*) (**13b**) to 31% (*R*) (**16b**). These results demonstrated that the electronegativity of the substituent on 2′- and 4′-positions of the phenyl group is critical to the enantioselectivity of *Gc*APRD mutants towards 2′-benzoylpyridine analogs, which is consistent with other previous studies of *Kp*ADH (Zhang et al. 2022).

In conclusion, a new mutation site, Phe56, in the large binding pocket of *Gc*APRD has been identified as important for eliminating steric hindrance. The new *Gc*APRD mutant, Phe56Ile, exhibited improved activity and excellent (*S*)-enantioselectivity for acetophenone and its halogenated analogs, as the entrance of the large pocket was enlarged through mutagenesis. Moreover, *Gc*APRD Trp288Ala exhibited catalytic activity towards 2-(4’-chlorobenzoyl)pyridine (**18a**). Therefore, Phe56Ile/Trp288Ala and Phe56Ala/Trp288Ala with further enhanced activities were constructed. In whole-cell-catalyzed reductions of diaryl ketones by Phe56Ile/Trp288Ala, significant increases in isolated yields of over 90% were obtained for most of the tested substrates. Moreover, excellent enantioselectivity of up to 97% *ee* (*R*) for the reduction of diaryl ketones was achieved. These results demonstrate that the *Gc*APRD mutants obtained in this study are promising biocatalysts for the preparation of valuable chiral diaryl alcohols, providing support for future immobilization studies of *Gc*APRD mutants and their applications in flow reactions.

## Supplementary Information

Below is the link to the electronic supplementary material.

Supplementary file1 (PDF 2.44 MB)

Supplementary file2 (PDF 33.7 MB)

## Data Availability

All data supporting the findings of this study are available within the paper and its Supplementary Information.
