# Supplementary material for: Substrate expansion of Geotrichum candidum alcohol dehydrogenase towards diaryl ketones by mutation
Source: Appl Microbiol Biotechnol. 2024 Dec 27;108(1):545. doi: 10.1007/s00253-024-13375-0 (PMC11680648; doi:10.1007/s00253-024-13375-0)
Supplement: Supplementary file 1 — Supplementary file1 (PDF 2.44 MB) [file 253_2024_13375_MOESM1_ESM.pdf]

**Journal name: Applied Microbiology and Biotechnology**

**Supplementary Materials**

# Substrate Expansion of *Geotrichum candidum* Alcohol Dehydrogenase towards Diaryl Ketones by Mutation

Zhongyao Tang[1], Yuuki Takagi[1], Afifa Ayu Koesoema[1], Tomoko Matsuda[1*]

Department of Life Science and Technology, School of Life Science and Technology, Institute of Science Tokyo, 4259 Nagatsuta-cho Midori-ku, Yokohama 226-8501, Japan

Corresponding author:
Tomoko Matsuda (tmatsuda@bio.titech.ac.jp, +81-45-924-5757)

Table of contents

1. Synthesis of **13a-17a** and **19a** by Grignard reaction ........ 1

2. Synthesis of *rac*-**3b-5b** and *rac*-**12b-19b** by sodium borohydride reduction ........ 1

3. The preparative scale asymmetric reduction of **5a** by *Gc*APRD Phe56Ile ........ 3

4. Asymmetric reduction of **12a-19a** by *Gc*APRD Trp288Ala, Phe56Ile/Trp288Ala and Phe56Ala/Trp288Ala ........ 3

Table S1. The primer sequences to prepare *Gc*APRD Phe56 mutants ........ 5

Table S2. The chiral GC analysis method and retention time of **1a-11a** and *rac*-**1b-11b** ........ 5

Table S3. The chiral HPLC analysis method and retention time of *rac*-**12b-19b** ........ 6

Figure S1. SDS-PAGE results of *Gc*APRD mutants on 12% of polyacrylamide gel ........ 7

Table S4. The ratio of the activity of Phe56Ile to wild type towards **1a-11a** ........ 7

Figure S2. Multiple sequences alignment ........ 8

Figure S3. Comparison of the location of the mutation site of *Gc*APRD with other medium-chain dehydrogenases/reductases (MDRs) ........ 9

References ........ 10

1. Synthesis of **13a-17a** and **19a** by Grignard reaction

**13a-17a** and **19a** were prepared according to a previously reported procedure (Tao et al. 2012). A mixture of 1-bromo-2-methylbenzene (1.664 g, 9.73 mmol) and magnesium (249.6 mg, 10.27 mmol) activated with $I_2$ (1.0 mg) in dry tetrahydrofuran (THF, 16 mL) was heated at 70°C, then refluxed for 4 h. After cooling to 0°C, a solution of 2-cyanopyridine (852.0 mg, 8.18 mmol) was added dropwise to the mixture. The mixture was warmed to 60°C and stirred overnight. The reaction was quenched with saturated $NH_4Cl$, and the product was extracted with $CH_2Cl_2$. The combined organic layer was dried over $Na_2SO_4$, and the solvent was removed by rotary evaporation under reduced pressure. The residue was then dissolved to $Et_2O$ (64 mL) and 6 N HCl (8 mL) was added to the solution. After stirring at room temperature for 30 min, saturated $NaHCO_3$ was added until the pH turned to 7. The product was extracted with $CH_2Cl_2$. The combined organic layer was dried over $Na_2SO_4$, and the solvent was removed by rotary evaporation under reduced pressure. The product was purified by silica gel column chromatography (hexane: ethyl acetate, 4:1) to give **13a**. **14a-17a** and **19a** were synthesized with the above procedure. The $^1$H-NMR spectrum of the products was obtained using 400 MHz Bruker Biospin Avance III 400A spectrometer (Bruker, USA), and compared with the spectra data reported in the literature (Tao et al. 2012; Liu et al. 2019). The results were as follows.

**13a** (324.3 mg, yield 20%, white solid), $^1$H-NMR (400 MHz, $CDCl_3$): $\delta$=8.68 (d, $J$=4.0 Hz, 1H), 8.07 (d, $J$=7.6 Hz, 1H), 7.87 (t, $J$=7.6 Hz, 1H), 7.45-7.38 (m, 3H), 7.29-7.23 (m, 2H), 2.38 (s, 3H).

**14a** (348.3 mg, yield 20%, brown oil), $^1$H-NMR (400 MHz, $CDCl_3$): $\delta$=8.72 (d, $J$=4.8 Hz, 1H), 8.01 (d, $J$=7.6 Hz, 1H), 7.89 (td, $J$=7.7 Hz, 1.7 Hz, 1H), 7.84-7.82 (m, 2H), 7.49-7.46 (m, 1H), 7.41-7.35 (m, 2H), 2.42 (s, 3H).

**15a** (432.9 mg, yield 26%, yellow oil), $^1$H-NMR (400 MHz, $CDCl_3$): $\delta$=8.71 (d, $J$=4.0 Hz, 1H), 8.02-7.97 (m, 3H), 7.90-7.86 (m, 1H), 7.48-7.45 (m, 1H), 7.29-7.27 (m, 2H), 2.42 (s, 3H).

**16a** (600.2 mg, yield 32%, yellow oil), $^1$H-NMR (400 MHz, $CDCl_3$): $\delta$=8.63 (d, $J$=4.8 Hz, 1H), 8.11 (d, $J$=8.0 Hz, 1H), 7.86 (td, $J$=7.8 Hz, 1.6 Hz, 1H), 7.49 (d, $J$=7.6 Hz, 1H), 7.45-7.33 (m, 4H).

**17a** (641.6 mg, yield 35%, white solid), $^1$H-NMR (400 MHz, $CDCl_3$): $\delta$=8.73 (d, $J$=4.4 Hz, 1H), 8.09-8.06 (m, 2H), 7.99-7.96 (m, 1H), 7.92 (td, $J$=7.6 Hz, 1.6 Hz, 1H), 7.58-7.55 (m, 1H), 7.53-7.50 (m, 1H),7.43 (t, $J$=7.8 Hz, 1H).

**19a** (152.9 mg, yield 9%, white solid), $^1$H-NMR (400 MHz, $CDCl_3$): $\delta$=8.71 (d, $J$=4.4 Hz, 1H), 8.17 (dd, $J$=8.8 Hz, 5.6 Hz, 2H), 8.05 (d, $J$=8.0 Hz, 1H), 7.90 (td, $J$=7.8 Hz, 1.3 Hz, 1H), 7.50-7.47 (m, 1H), 7.15 (t, $J$=8.6 Hz, 2H).

2. Synthesis of *rac*-**3b-5b** and *rac*-**12b-19b** by sodium borohydride reduction

$NaBH_4$ (129.0 mg, 3.41 mmol) was added to **3a** (580.6 mg, 4.20 mmol) in dry ethanol (20 mL)

and stirred in an ice bath. The suspension was further stirred at room temperature until the reaction was detected completely by TLC. Then the reaction was quenched by the addition of 1N HCl until pH turned to 7-8. After removed ethanol, the residue was extracted with $Et_2O$, then washed with saturated $NaHCO_3$. The combined $Et_2O$ layers were dried by $MgSO_4$ and the solvent was removed by rotary evaporation. The product was purified by silica gel column chromatography (hexane: ethyl acetate, 2:1~4:1) to give *rac*-**3b**. *Rac*-**4b**, *rac*-**5b** and *rac*-**12b-19b** were synthesized with the above procedure. The $^1$H-NMR spectrum of the products was obtained using 400 MHz Bruker Biospin Avance III 400A spectrometer (Bruker, USA), and compared with the spectra data reported in the literature (Li et al. 2014; Liu et al. 2019; Nian et al. 2019; Liu et al. 2021). The results were as follows.

*rac*-**3b** (508.7 mg, yield 77%, colorless oil), $^1$H-NMR (400 MHz, $CDCl_3$): $\delta$=7.33-7.28 (m, 1H), 7.14-7.09 (m, 2H), 6.95 (td, *J*=8.4 Hz, 2.0 Hz, 1H), 4.90 (q, *J*=6.5 Hz, 1H), 1.84 (brs, 1H), 1.49 (d, *J*=6.4 Hz, 3H).

*rac*-**4b** (652.3 mg, yield 86%, colorless oil), $^1$H-NMR (400 MHz, $CDCl_3$): $\delta$=7.36-7.33 (m, 2H), 7.06-7.00 (m, 2H), 4.90 (q, *J*=6.4 Hz, 1H), 1.77 (brs, 1H),1.48 (d, *J*=6.4 Hz, 3H).

*rac*-**5b** (709.1 mg, yield 78%, colorless oil), $^1$H-NMR (400 MHz, $CDCl_3$): $\delta$=7.60 (dd, *J*=7.8 Hz, 1.4 Hz, 1H), 7.34-7.28 (m, 2H), 7.20 (td, *J*=7.6 Hz, 1.6 Hz, 1H), 5.30 (q, *J*=6.4 Hz, 1H), 1.95 (brs, 1H), 1.50 (d, *J*=6.4 Hz, 3H).

*rac*-**12b** (161.3 mg, yield 84%, white solid), $^1$H-NMR (400 MHz, $CDCl_3$): $\delta$=8.56 (d, *J*=4.8 Hz, 1H), 7.61 (td, *J*=7.6 Hz, 1.6 Hz, 1H), 7.39-7.31 (m, 4H), 7.29-7.24 (m, 1H), 7.18 (dd, *J*=7.2 Hz, 5.2 Hz, 1H), 7.14 (d, *J*=8.0 Hz, 1H), 5.75 (d, *J*=4.0 Hz, 1H), 5.23 (d, *J*=4.4 Hz, 1H).

*rac*-**13b** (92.5 mg, yield 86%, colorless oil), $^1$H-NMR (400 MHz, $CDCl_3$): $\delta$=8.56 (d, *J*=4.8 Hz, 1H), 7.58 (td, *J*=7.6 Hz, 1.6 Hz, 1H), 7.26-7.24 (m, 1H), 7.19-7.14 (m, 4H), 7.02 (d, *J*=8.0 Hz, 1H), 5.96 (s, 1H), 5.27 (brs, 1H), 2.32 (s, 3H).

*rac*-**14b** (75.2 mg, yield 66%, white solid), $^1$H-NMR (400 MHz, $CDCl_3$): $\delta$=8.56 (d, *J*=4.8 Hz, 1H), 7.61 (td, *J*=7.8 Hz, 1.6 Hz, 1H), 7.25-7.14 (m, 5H), 7.08 (d, *J*=7.2 Hz, 1H), 5.71 (s, 1H), 5.19 (d, *J*=3.6 Hz, 1H), 2.32 (s, 3H).

*rac*-**15b** (109.1 mg, yield 90%, white solid), $^1$H-NMR (400 MHz, $CDCl_3$): $\delta$=8.55 (d, *J*=4.8 Hz, 1H), 7.60 (td, *J*=7.7 Hz, 1.7 Hz, 1H), 7.26-7.24 (m, 2H), 7.19-7.13 (m, 4H), 5.72 (d, *J*=3.6 Hz, 1H), 5.15 (d, *J*=4.0 Hz, 1H), 2.32 (s, 3H).

*rac*-**16b** (85.1 mg, yield 69%, colorless oil), $^1$H-NMR (400 MHz, $CDCl_3$): $\delta$=8.57 (d, *J*=4.8 Hz, 1H), 7.62 (td, *J*=7.7 Hz, 1.7 Hz, 1H), 7.41-7.37 (m, 2H), 7.25-7.18 (m, 4H), 6.27 (d, *J*=3.2 Hz 1H), 5.43 (d, *J*=4.4 Hz 1H).

*rac*-**17b** (94.5 mg, yield 82%, colorless oil), $^1$H-NMR (400 MHz, $CDCl_3$): $\delta$=8.57 (d, *J*=4.8 Hz, 1H), 7.64 (td, *J*=7.7 Hz, 1.7 Hz, 1H), 7.37 (s, 1H), 7.28-7.20 (m, 4H), 7.15 (d, *J*=8.0 Hz 1H), 5.71 (s, 1H), 5.28 (d, *J*=3.6 Hz 1H).

*rac*-**18b** (215.1 mg, yield >99%, white solid), $^{1}$H-NMR (400 MHz, $CDCl_3$): $\delta$=8.56 (d, *J*=4.8 Hz, 1H), 7.63 (td, *J*=7.7 Hz, 1.7 Hz, 1H), 7.33-7.28 (m, 4H), 7.22-7.19 (m, 1H), 7.12 (d, *J*=7.6 Hz 1H), 5.72 (s, 1H), 5.25 (s, 1H).
*rac*-**19b** (81.5 mg, yield 78%, white solid), $^{1}$H-NMR (400 MHz, $CDCl_3$): $\delta$=8.56 (d, *J*=4.8 Hz, 1H), 7.62 (td, *J*=7.7 Hz, 1.7 Hz, 1H), 7.36-7.32 (m, 2H), 7.22-7.19 (m, 1H), 7.12 (d, *J*=8.0 Hz, 1H), 7.03-6.99 (m, 2H), 5.73 (s, 1H), 5.26 (s, 1H).

3. The preparative scale asymmetric reduction of **5a** by *Gc*APRD Phe56Ile

**5a** (52.2 mg, 0.34 mmol) was converted to (*S*)-**5b** (41.1 mg, 0.26 mmol, yield 77%, colorless oil), $[\alpha]_D^{20}$= -58.4 (c=0.50, $CHCl_3$, >99% *ee*) (*lit.*(Liang et al. 2018) $[\alpha]_D^{20}$= –56.8 (c=1.0, $CHCl_3$, >99% *ee* (*S*)); $^{1}$H-NMR (400 MHz, $CDCl_3$): $\delta$=7.59 (dd, *J*=7.8 Hz, 1.4 Hz, 1H), 7.33-7.28 (m, 2H), 7.19 (td, *J*=7.6 Hz, 1.6 Hz, 1H), 5.29 (q, *J*=6.4 Hz, 1H), 1.99 (brs, 1H), 1.49 (d, *J*=6.4 Hz, 3H).

4. Asymmetric reduction of **12a-19a** by *Gc*APRD Trp288Ala, Phe56Ile/Trp288Ala and Phe56Ala/Trp288Ala

Reduction of **12a**

Trp288Ala: **12a** (6.6 mg, 0.036 mmol, 12 mM) was converted to (*S*)-**12b** (5.1 mg, 0.028 mmol, yield 76%).

Phe56Ile/Trp288Ala: **12a** (6.1 mg, 0.033 mmol, 11 mM) was converted to (*S*)-**12b** (5.7 mg, 0.031 mmol, yield 92%).

Phe56Ala/Trp288Ala: **12a** (6.4 mg, 0.035 mmol, 12 mM) was converted to (*R*)-**12b** (3.5 mg, 0.019 mmol, yield 54%).

$^{1}$H-NMR (400 MHz, $CDCl_3$): $\delta$=8.57 (d, *J*=4.8 Hz, 1H), 7.62 (t, *J*=7.8 Hz, 1H), 7.39-7.27 (m, 5H), 7.21-7.18 (m, 1H), 7.15 (d, *J*=8.0 Hz, 1H), 5.75 (s, 1H), 5.26 (d, *J*=3.6 Hz, 1H).

Reduction of **13a**

Trp288Ala: **13a** (6.2 mg, 0.031mmol, 11 mM) was converted to (*S*)-**13b** (4.2 mg, 0.021 mmol, yield 67%).

Phe56Ile/Trp288Ala: **13a** (8.0 mg, 0.041 mmol, 14 mM) was converted to (*R*)-**13b** (7.7 mg, 0.039 mmol, yield 95%).

Phe56Ala/Trp288Ala: **13a** (6.0 mg, 0.030 mmol, 10 mM) was converted to (*R*)-**13b** (3.8 mg, 0.019 mmol, yield 63%).

$^{1}$H-NMR (400 MHz, $CDCl_3$): $\delta$=8.60 (d, *J*=4.8 Hz, 1H), 7.60 (td, *J*=7.6 Hz, 1.6 Hz, 1H), 7.24-7.15 (m, 5H), 7.02 (d, *J*=8.0 Hz, 1H), 5.96 (s, 1H), 5.11 (brs, 1H), 2.34 (s, 3H).

Reduction of **14a**

Trp288Ala: **14a** (5.9 mg, 0.030 mmol, 10 mM) was converted to (*R*)-**14b** (4.5 mg, 0.023 mmol,

yield 76%).

Phe56Ile/Trp288Ala: **14a** (8.2 mg, 0.042 mmol, 14 mM) was converted to (*R*)-**14b** (6.6 mg, 0.031 mmol, yield 80%).

Phe56Ala/Trp288Ala: **14a** (6.2 mg, 0.031 mmol, 11 mM) was converted to (*R*)-**14b** (5.2 mg, 0.026 mmol, yield 83%).

$^1$H-NMR (400 MHz, $CDCl_3$): $\delta$=8.57 (d, *J*=4.8 Hz, 1H), 7.61 (td, *J*=7.6 Hz, 1.6 Hz, 1H), 7.24-7.14 (m, 5H), 7.09 (d, *J*=7.2 Hz, 1H), 5.71 (s, 1H), 5.22 (brd, *J*=3.2 Hz, 1H), 2.33 (s, 3H).

Reduction of **15a**

Trp288Ala: **15a** (6.3 mg, 0.032 mmol, 11 mM) was converted to (*S*)-**15b** (5.1 mg, 0.026 mmol, yield 80%).

Phe56Ile/Trp288Ala: **15a** (9.0 mg, 0.046 mmol, 15 mM) was converted to (*S*)-**15b** (8.7 mg, 0.044 mmol, yield 96%).

Phe56Ala/Trp288Ala: **15a** (6.8 mg, 0.034 mmol, 12 mM) was converted to (*R*)-**15b** (5.6 mg, 0.028 mmol, yield 82%).

$^1$H-NMR (400 MHz, $CDCl_3$): $\delta$=8.56 (d, *J*=4.8 Hz, 1H), 7.61 (td, *J*=7.7 Hz, 1.7 Hz, 1H), 7.26-7.25 (m, 2H), 7.20-7.13 (m, 4H), 5.72 (s, 1H), 5.18 (brd, *J*=2.8 Hz, 1H), 2.32 (s, 3H).

Reduction of **16a**

Trp288Ala: **16a** (6.6 mg, 0.030 mmol, 10 mM) was converted to (*R*)-**16b** (5.6 mg, 0.025 mmol, yield 84%).

Phe56Ile/Trp288Ala: **16a** (10.9 mg, 0.050 mmol, 17 mM) was converted to (*R*)-**16b** (10.5 mg, 0.048 mmol, yield 95%).

Phe56Ala/Trp288Ala: **16a** (6.8 mg, 0.031 mmol, 10 mM) was converted to (*R*)-**16b** (4.9 mg, 0.022 mmol, yield 71%).

$^1$H-NMR (400 MHz, $CDCl_3$): $\delta$=8.57 (d, *J*=4.8 Hz, 1H), 7.62 (td, *J*=7.6 Hz, 16 Hz, 1H), 7.40-7.37 (m, 2H), 7.25-7.18 (m, 4H), 6.27 (s, 1H), 5.43 (brs, 1H).

Reduction of **17a**

Trp288Ala: **17a** (6.8 mg, 0.031 mmol, 10 mM) was converted to (*R*)-**17b** (5.7 mg, 0.026 mmol, yield 83%).

Phe56Ile/Trp288Ala: **17a** (7.0 mg, 0.032 mmol, 11 mM) was converted to (*R*)-**17b** (6.6 mg, 0.300 mmol, yield 93%).

Phe56Ala/Trp288Ala: **17a** (6.6 mg, 0.030 mmol, 10 mM) was converted to (*R*)-**17b** (2.2 mg, 0.010 mmol, yield 33%).

$^1$H-NMR (400 MHz, $CDCl_3$): $\delta$=8.57 (d, *J*=4.8 Hz, 1H), 7.64 (td, *J*=7.7 Hz, 1.7 Hz, 1H), 7.38 (s, 1H), 7.28-7.26 (m, 2H), 7.25-7.20 (m, 2H), 7.15 (d, *J*=7.6 Hz, 1H), 5.71 (d, *J*=3.6 Hz, 1H), 5.27 (brd, *J*=4.0 Hz, 1H).

Reduction of **18a**

Trp288Ala: **18a** (8.3 mg, 0.038 mmol, 13 mM) was converted to (*R*)-**18b** (4.9 mg, 0.022 mmol, yield 56%).

Phe56Ile/Trp288Ala: **18a** (7.3 mg, 0.034 mmol, 11 mM) was converted to (*R*)-**18b** (7.1 mg, 0.032 mmol, yield 96%).

Phe56Ala/Trp288Ala: **18a** (6.7 mg, 0.031 mmol, 10 mM) was converted to (*R*)-**18b** (3.5 mg, 0.016 mmol, yield 51%).

$^1$H-NMR (400 MHz, $CDCl_3$): $\delta$=8.57 (d, *J*=4.8 Hz, 1H), 7.63 (t, *J*=7.8 Hz, 1H), 7.33-7.29 (m, 4H), 7.23-7.20 (m, 1H), 7.12 (d, *J*=8.0 Hz, 1H), 5.72 (d, *J*=3.6 Hz, 1H), 5.27 (d, *J*=4.4 Hz, 1H).

Reduction of **19a**

Trp288Ala: **19a** (6.6 mg, 0.033 mmol, 11 mM) was converted to (*R*)-**19b** (4.9 mg, 0.024 mmol, yield 74%).

Phe56Ile/Trp288Ala: **19a** (6.3 mg, 0.031 mmol, 10 mM) was converted to (*R*)-**19b** (5.7 mg, 0.028 mmol, yield 90%).

Phe56Ala/Trp288Ala: **19a** (6.5 mg, 0.032 mmol, 11 mM) was converted to (*R*)-**19b** (5.8 mg, 0.029 mmol, yield 88%).

$^1$H-NMR (400 MHz, $CDCl_3$): $\delta$=8.57 (d, *J*=5.2 Hz, 1H), 7.63 (td, *J*=7.7 Hz, 1.7 Hz, 1H), 7.36-7.32 (m, 2H), 7.22-7.19 (m, 1H), 7.11 (d, *J*=7.6 Hz, 1H), 7.05-6.99 (m, 2H), 5.73 (s, 1H), 5.24 (d, *J*=3.2 Hz, 1H).

Table S1. The primer sequences to prepare *Gc*APRD Phe56 mutants

| Mutants | Forward primers (5'→3') | Reverse primer (5'→3') |
|---|---|---|
| Phe56Ala | GCNCCCATTCCCTCCAACAGCGTT | AGAGCCTTGGAGAATGT GCAGGTC |
| Phe56Val | GTNCCCATTCCCTCCAACAGCGTT | |
| Phe56Ile | ATHCCCATTCCCTCCAACAGCGTT | |
| Phe56His | CAYCCCATTCCCTCCAACAGCGTT | |

Table S2. The chiral GC analysis method and retention time of **1a-11a** and *rac*-**1b-11b**

| Compound | Condition | Retention time (min) | | | |
|---|---|---|---|---|---|
| | | Internal standard (3-methyl-1-butanol) | Ketone (**a**) | Alcohol (**b**)[a] | |
| | | | | *S* | *R* |
| **1** | A | 9.1 | 15.3 | 19.9 | 19.6 |

Table S2. Continued

| | | | | | |
|---|---|---|---|---|---|
| **2** | B | 6.7 | 9.0 | 12.5 | 12.3 |
| **3** | B | 6.7 | 9.8 | 12.9 | 12.6 |
| **4** | B | 6.7 | 10.1 | 12.8 | 12.5 |
| **5** | B | 6.7 | 11.9 | 17.0 | 16.0 |
| **6** | B | 6.7 | 12.4 | 16.8 | 16.4 |
| **7** | B | 6.7 | 13.0 | 17.2 | 16.6 |
| **8** | C | 6.7 | 15.3 | 22.9 | 22.2 |
| **9** | D | 6.7 | 13.4 | 21.8 | 19.4 |
| **10** | D | 6.7 | 14.0 | 20.4 | 19.7 |
| **11** | D | 6.7 | 15.0 | 21.3 | 20.4 |

Condition A: 40 °C, 1 min, 5 °C/min, 150 °C, 10 min; Condition B: 40 °C, 1 min, 10 °C/min, 150 °C, 10 min; Condition C: 40 °C, 1 min, 10 °C/min, 160 °C, 15 min; Condition D: 40 °C, 1 min, 10 °C/min, 150 °C, 15 min.

[a]The retention time of *R* and *S* enantiomers was determined by referring to the literature (Koesoema et al. 2019a; Koesoema et al. 2020).

Table S3. The chiral HPLC analysis method and retention time of *rac*-**12b-19b**

| Compound | Retention time (min)[a] | |
|---|---|---|
| | *S* | *R* |
| **12b** | 20.2 | 23.6 |
| **13b** | 17.2 | 19.5 |
| **14b** | 18.1 | 22.0 |
| **15b** | 20.0 | 22.7 |
| **16b** | 17.5 | 18.4 |
| **17b** | 18.7 | 22.6 |
| **18b** | 20.8 | 25.9 |
| **19b** | 20.5 | 24.4 |

HPLC condition: hexane: 2-propanol=9:1, 0.5 mL/min, 254 nm, room temperature.

[a]The retention time of *R* and *S* enantiomers was determined by referring to the literature (Liu et al. 2019; Nian et al. 2019).

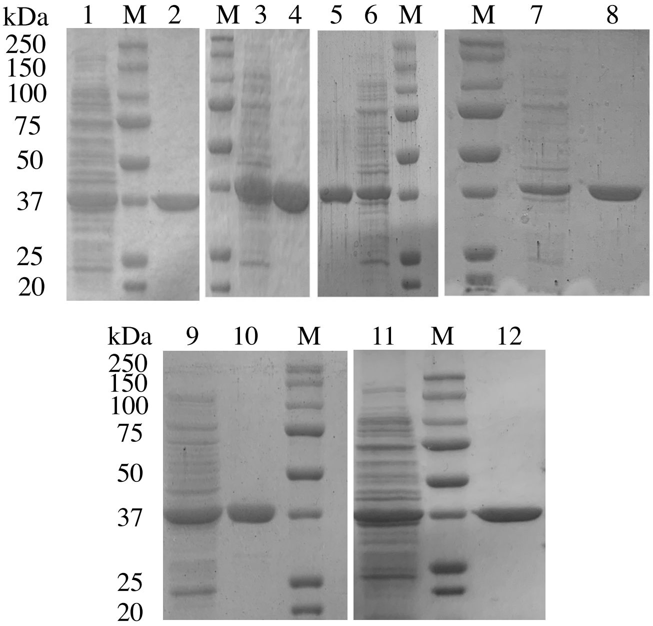


Figure S1. SDS-PAGE results of *Gc*APRD mutants on 12% of polyacrylamide gel (Lane M, molecular weight standards; Lane 1, cell-free extract of Phe56Ala; Lane 2, purified Phe56Ala; Lane 3, cell-free extract of Phe56Val; Lane 4, purified Phe56Val; Lane 5, purified Phe56Ile; Lane 6, cell-free extract of Phe56Ile; Lane 7, cell-free extract of Phe56His; Lane 8, purified Phe56His; Lane 9, cell-free extract of Phe56Ile/Trp288Ala; Lane 10, purified Phe56Ile/Trp288Ala; Lane 11, cell-free extract of Phe56Ala/Trp288Ala; Lane 12, purified Phe56Ala/Trp288Ala.)

Table S4. The ratio[a] of the activity of Phe56Ile to wild type towards **1a-11a**

| Substituent | 2’-substituted | 3’-substituted | 4’-substituted | 3’, 4’ -disubstituted |
|---|---|---|---|---|
| H | **1a** 2.2 | | | |
| F | **2a** 3.3 | **3a** 3.3 | **4a** 4.8 | - |
| Cl | **5a** 6.5 | **6a** 5.6 | **7a** 4.2 | **8a** 1.5 |
| Br | **9a** 4.5 | **10a** 1.1 | **11a** 1.9 | - |

[a]The ratio was calculated using the relative activity in Fig. 2 and the specific activity in Table 1. Ratio = (Relative activity of Phe56Ile / Relative activity of wild type) × (48.8 μmol/min/mg / 22.2 μmol/min/mg)

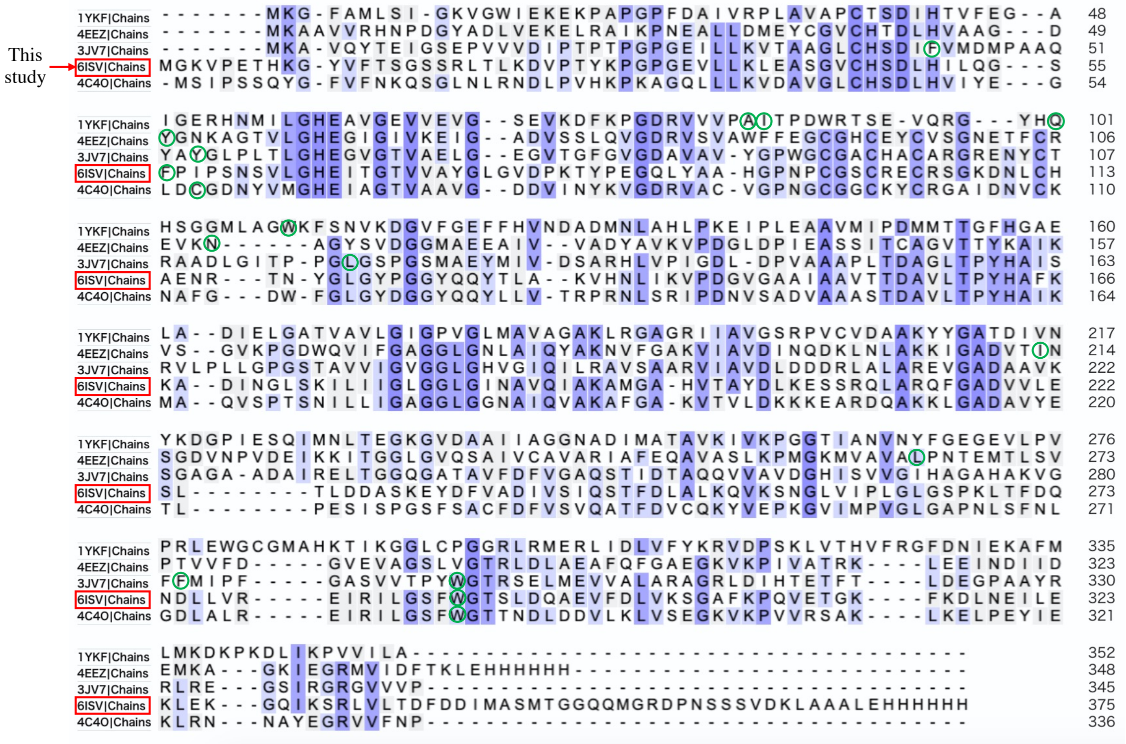


Figure S2. Multiple sequences alignment (Alcohol dehydrogenase from *Thermoanaerobacter brockii* (*Tb*SADH, PDB ID: 1YKF) (Korkhin et al. 1998)[a]; *Lactococcus lactis* alcohol dehydrogenase (LlAdhA, PDB ID: 4EEZ) (Liu et al. 2012); alcohol dehydrogenase ADH-A from *Rhodococcus ruber* DSM 44541(ADH-A, PDB ID: 3JV7) (Kroutil and Gruber 2010); acetophenone reductase from *Geotrichum candidum* NBRC 4597 (*Gc*APRD, PDB ID: 6ISV) (Koesoema et al. 2019b); carbonyl reductase from *Candida parapsilosis* (CPCR2/*cp*ADH5, PDB ID: 4C4O) (Man et al. 2014); Green circle: mutation site of each enzyme.)

[a]Alcohol dehydrogenase from *Thermoanaerobacter ethanolicus* (*Te*SADH) is identical in sequence to *Tb*SADH (Bsharat et al. 2017).

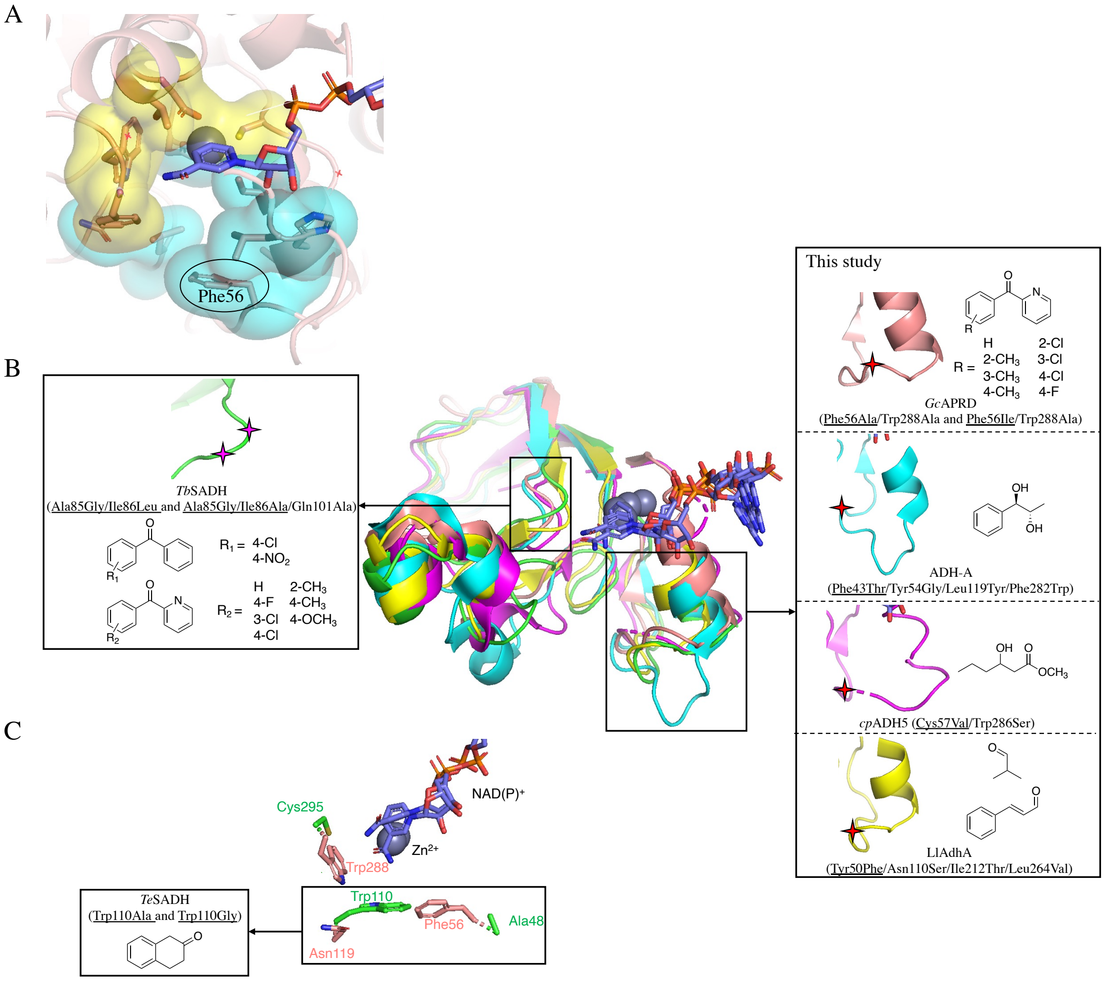


Figure S3. Comparison of the location of the mutation site of *Gc*ARPD with other medium-chain dehydrogenases/reductases (MDRs) [a, b]. (A) Crystal structure of binding pockets of *Gc*APRD containing the target loop with Phe56; (B) Comparison of *Gc*ARPD with other MDRs; (C) Spatial alignment result of *Gc*ARPD and *Te*SADH. (Yellow cavity: small binding pocket in *Gc*APRD; blue cavity: large binding pocket in *Gc*APRD; peach pink: *Gc*APRD; blue: ADH-A; pink: *cp*ADH5; yellow: LlAdhA; green: *Tb*SADH or *Te*SADH; gray sphere: $Zn^{2+}$ and violet stick: $NAD(P)^{+}$.)

[a]The crystal structure of *Gc*APRD was provided from literature (Koesoema et al. 2019b).

[b]The mutation site of other MDRs were provided from the following literatures: *Tb*SADH (Ala85Gly/Ile86Leu and Ala85Gly/Ile86Ala/Gln101Ala) (Liu et al. 2019; Qu et al. 2019); ADH-A (Phe43Thr/Tyr54Gly/Leu119Tyr/Phe282Trp) (Maurer et al. 2018); *cp*ADH5 (Cys57Val/Trp286Ser) (Ensari et al. 2018); LlAdhA (Tyr50Phe/Asn110Ser/Ile212Thr/Leu264Val) (Liu et al. 2012); *Te*SADH (Trp110Ala and Trp110Gly) (Musa et al. 2007; Bsharat et al. 2017).

References

Bsharat O, Musa MM, Vieille C, Oladepo SA, Takahashi M, Hamdan SM (2017) Asymmetric reduction of substituted 2-tetralones by *Thermoanaerobacter pseudoethanolicus* secondary alcohol dehydrogenase. ChemCatChem 9:1487–1493. https://doi.org/10.1002/cctc.201601618

Ensari Y, Dhoke G V, Davari MD, Ruff AJ, Schwaneberg U (2018) A comparative reengineering study of *cp*ADH5 through iterative and simultaneous multisite saturation mutagenesis. ChemBioChem 19:1563–1569. https://doi.org/10.1002/cbic.201800159

Koesoema AA, Standley DM, Ohshima S, Tamura M, Matsuda T (2020) Control of enantioselectivity in the enzymatic reduction of halogenated acetophenone analogs by substituent positions and sizes. Tetrahedron Lett 61:151820. https://doi.org/10.1016/j.tetlet.2020.151820

Koesoema AA, Sugiyama Y, T.sriwong K, Xu Z, Verina S, Standley DM, Senda M, Senda T, Matsuda T (2019a) Reversible control of enantioselectivity by the length of ketone substituent in biocatalytic reduction. Appl Microbiol Biotechnol 103:9529–9541. https://doi.org/10.1007/S00253-019-10206-5

Koesoema AA, Sugiyama Y, Xu Z, Standley DM, Senda M, Senda T, Matsuda T (2019b) Structural basis for a highly (*S*)-enantioselective reductase towards aliphatic ketones with only one carbon difference between side chain. Appl Microbiol Biotechnol 103:9543–9553. https://doi.org/10.1007/s00253-019-10093-w

Korkhin Y, Gilboa AJK, Peretz M, Bogin O, Burstein Y, Frolow F (1998) NADP-dependent bacterial alcohol dehydrogenases : crystal structure, cofactor-binding and cofactor specificity of the ADHs of *Clostridium beijerinckii* and *Thermoanaerobacter brockii*. J Mol Biol 278:967–981. https://doi.org/10.1006/jmbi.1998.1750

Kroutil W, Gruber K (2010) Structural insights into substrate specificity and solvent tolerance in alcohol dehydrogenase ADH-‘A’ from *Rhodococcus ruber* DSM 44541. Chem Commun 46:6314–6316. https://doi.org/10.1039/c0cc00929f

Li M, Li B, Xia H, Ye D, Wu J, Shi Y (2014) Mesoporous silica KIT-6 supported superparamagnetic $CuFe_2O_4$ nanoparticles for catalytic asymmetric hydrosilylation of ketones in air. Green Chem 16:2680–2688. https://doi.org/10.1039/c3gc42638f

Liang Z, Yang T, Gu G, Dang L, Zhang X (2018) Scope and mechanism on iridium-*f*-amphamide catalyzed asymmetric hydrogenation of ketones. Chinese J Chem 36:851–856.

https://doi.org/10.1002/cjoc.201800129

Liu B, Qu G, Li J, Fan W, Ma J, Xu Y, Nie Y, Sun Z (2019) Conformational dynamics-guided loop engineering of an alcohol dehydrogenase: Capture, turnover and enantioselective transformation of difficult-to-reduce ketones. Adv Synth Catal 361:3182–3190. https://doi.org/10.1002/adsc.201900249

Liu J, Li W, Li Y, Liu Y, Ke Z (2021) Selective C-alkylationbetween alcohols catalyzed by N-heterocyclic carbene molybdenum. Chem - An Asian J 16:3124–3128. https://doi.org/10.1002/asia.202100959

Liu X, Bastian S, Snow CD, Brustad EM, Saleski TE, Xu J, Meinhold P, Arnold FH (2012) Structure-guided engineering of *Lactococcus lactis* alcohol dehydrogenase LlAdhA for improved conversion of isobutyraldehyde to isobutanol. J Biotechnol 164:188–195. https://doi.org/10.1016/j.jbiotec.2012.08.008

Man H, Loderer C, Ansorge-schumacher MB, Grogan G (2014) Structure of NADH-dependent carbonyl reductase (CPCR2) from *Candida parapsilosis* provides insight into mutations that improve catalytic properties. ChemCatChem 6:1103–1111. https://doi.org/10.1002/cctc.201300788

Maurer D, Enugala TR, Hamnevik E, Bauer P, Lüking M, Petrović D, Hillier H, Kamerlin SCL, Dobritzsch D, Widersten M (2018) Stereo- and regioselectivity in catalyzed transformation of a 1,2-disubstituted vicinal diol and the corresponding diketone by wild type and laboratory evolved alcohol dehydrogenases. ACS Catal 8:7526–7538. https://doi.org/10.1021/acscatal.8b01762

Musa MM, Ziegelmann-Fjeld KI, Vieille C, Zeikus JG, Phillips RS (2007) Asymmetric reduction and oxidation of aromatic ketones and alcohols using W110A secondary alcohol dehydrogenase from *Thermoanaerobacter ethanolicus*. J Org Chem 72:30–34. https://doi.org/https://doi.org/10.1021/jo0616097

Nian S, Ling F, Chen J, Wang Z, Shen H, Yi X, Yang Y, She Y, Zhong W (2019) Highly enantioselective hydrogenation of non-*ortho*-substituted 2-pyridyl aryl ketones via iridium-*f*-diaphos catalysis. Org Lett 21:5392–5396. https://doi.org/10.1021/acs.orglett.9b01415

Qu G, Liu B, Jiang Y, Nie Y, Yu H, Sun Z (2019) Laboratory evolution of an alcohol dehydrogenase towards enantioselective reduction of difficult-to-reduce ketones. Bioresour Bioprocess 6:18. https://doi.org/10.1186/s40643-019-0253-9

Tao X, Li W, Ma X, Li X, Fan W, Xie X, Ayad T, Ratovelomanana-Vidal V, Zhang Z (2012)

Ruthenium-catalyzed enantioselective hydrogenation of aryl-pyridyl ketones. J Org Chem 77:612–616. https://doi.org/10.1021/jo202204j
