# Supplementary material for: Substrate expansion of Geotrichum candidum alcohol dehydrogenase towards diaryl ketones by mutation
Source: Appl Microbiol Biotechnol. 2024 Dec 27;108(1):545. doi: 10.1007/s00253-024-13375-0 (PMC11680648; doi:10.1007/s00253-024-13375-0)
Supplement: Supplementary file 2 — Supplementary file2 (PDF 33.7 MB) [file 253_2024_13375_MOESM2_ESM.pdf]

Journal name: Applied Microbiology and Biotechnology

Appendix

Substrate Expansion of *Geotrichum candidum* Alcohol Dehydrogenase towards Diaryl Ketones by Mutation

Zhongyao Tang[1], Yuuki Takagi[1], Afifa Ayu Koesoema[1], Tomoko Matsuda[1*]

Department of Life Science and Technology, School of Life Science and Technology, Institute of Science Tokyo, 4259 Nagatsuta-cho Midori-ku, Yokohama 226-8501, Japan

Corresponding author:
Tomoko Matsuda (tmatsuda@bio.titech.ac.jp, +81-45-924-5757)

Table of contents

1. ^{1}H-NMR of diaryl ketone **13a-17a** and **19a**...1

2. ^{1}H-NMR of racemic alcohol **3b-5b** and **12b-19b**..4

3. GC results of racemic alcohol **1b-11b** and Phe56Ile catalyzed analytical scale asymmetric reduction of **1a-11a**...10

4. Phe56Ile catalyzed preparative scale asymmetric reduction of **5a**...21

5. HPLC results of racemic alcohol **12b-19b** and *Gc*APRD mutants catalyzed reduction of **12a-19a**...22

6. ^{1}H-NMR of the asymmetric reduction of **12a-19a** by *Gc*APRD mutants...............................30

1. ^{1}H-NMR of diaryl ketone **13a-17a** and **19a**

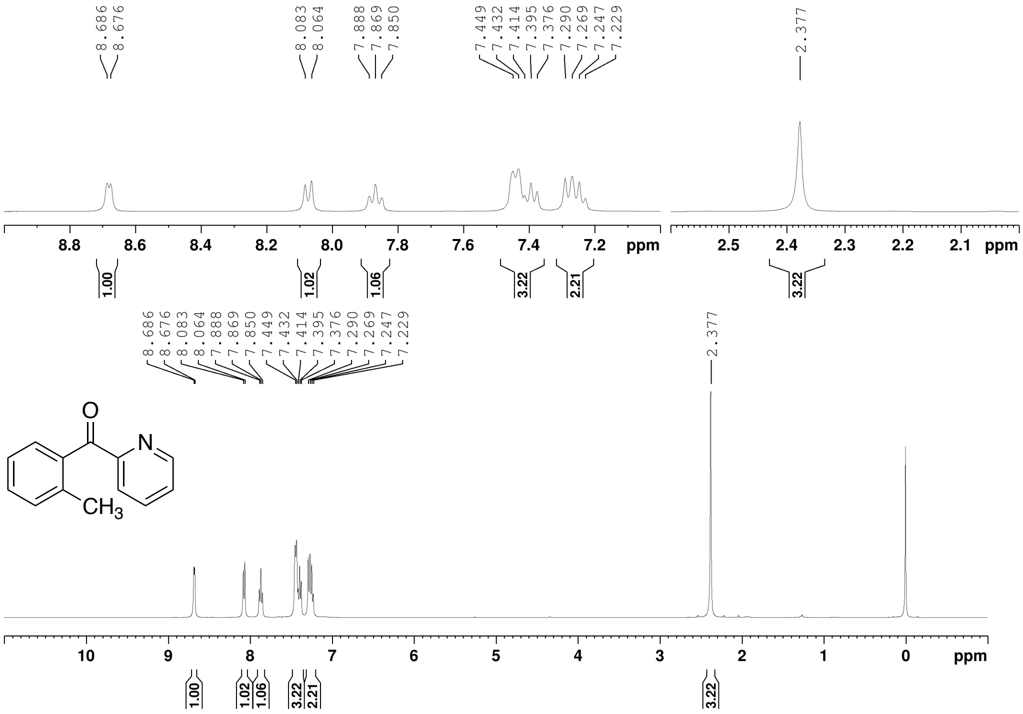

^{1}H-NMR of **13a**

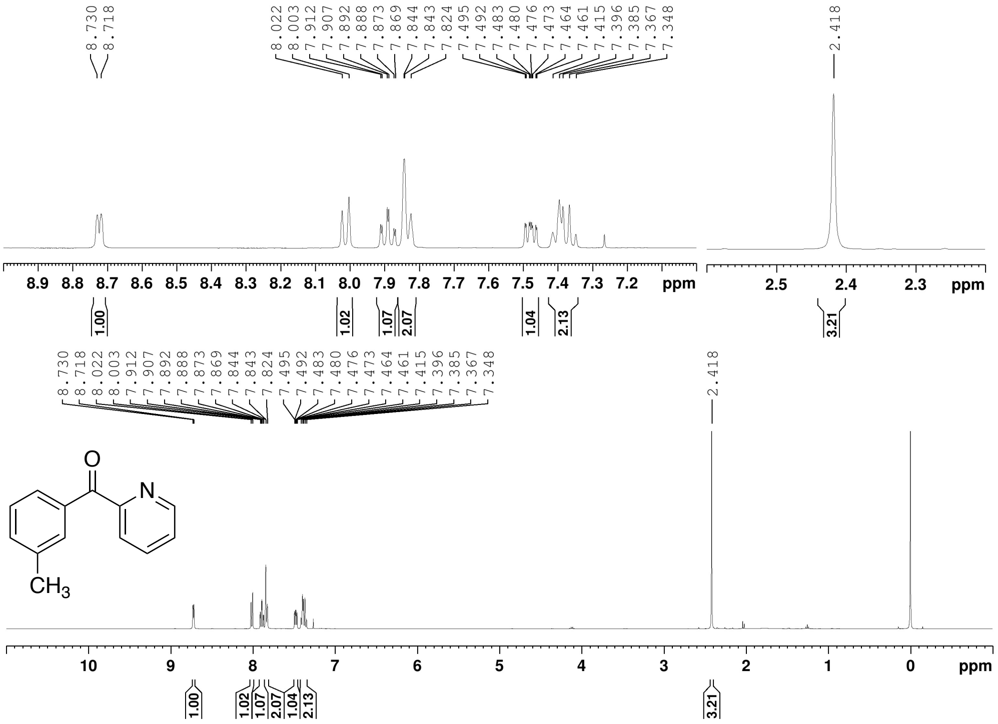

^{1}H-NMR of **14a**

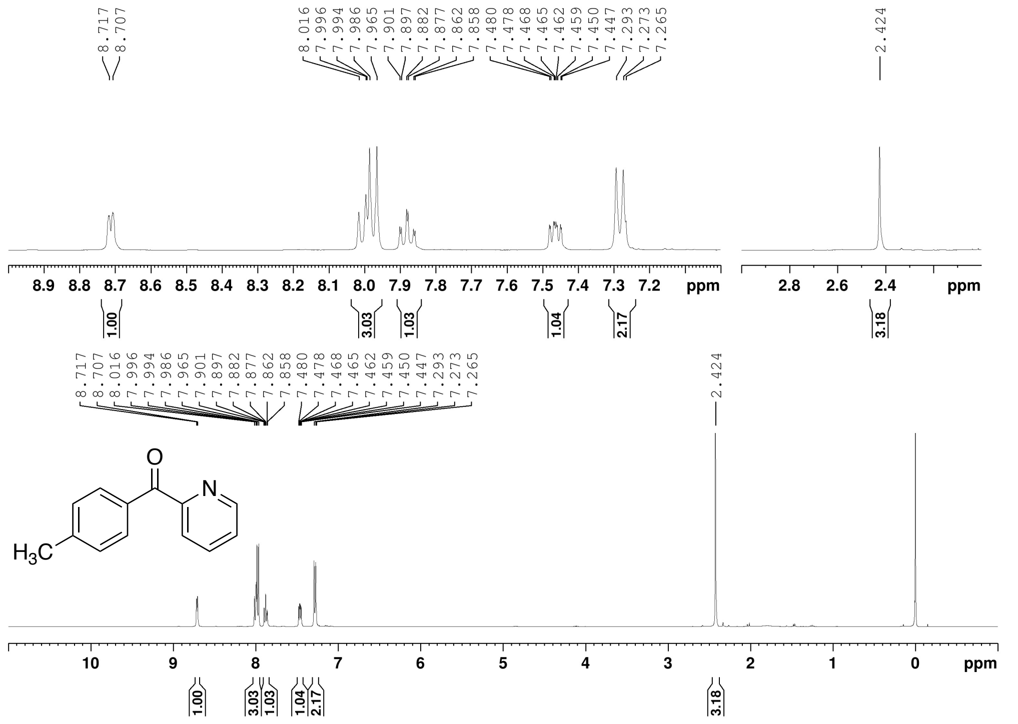

^{1}H-NMR of **15a**

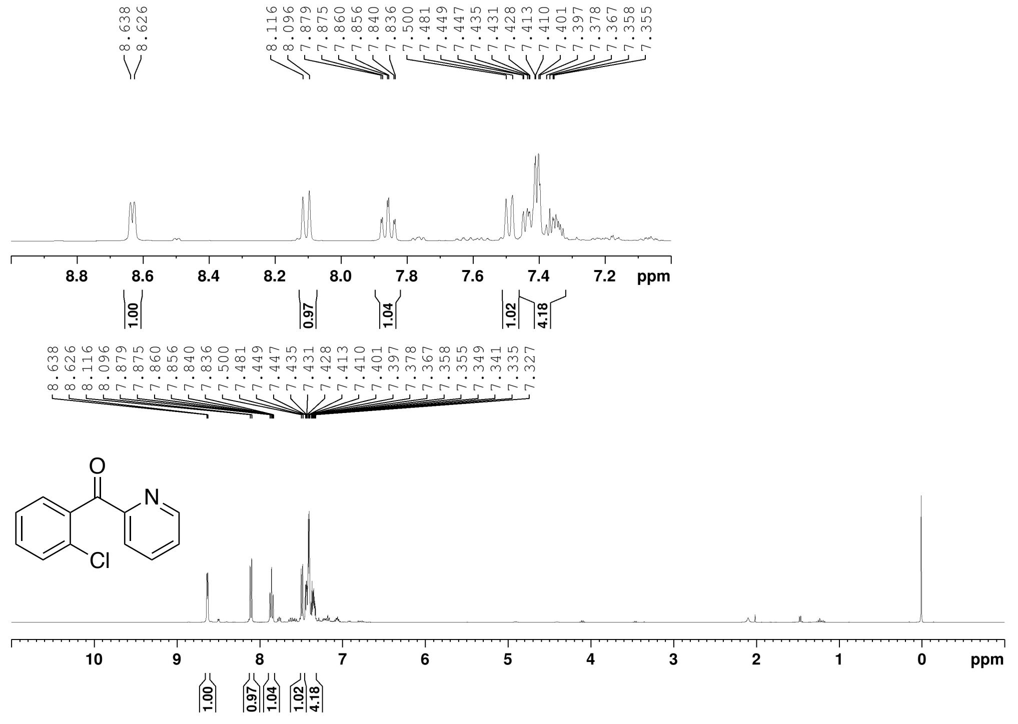

^{1}H-NMR of **16a**

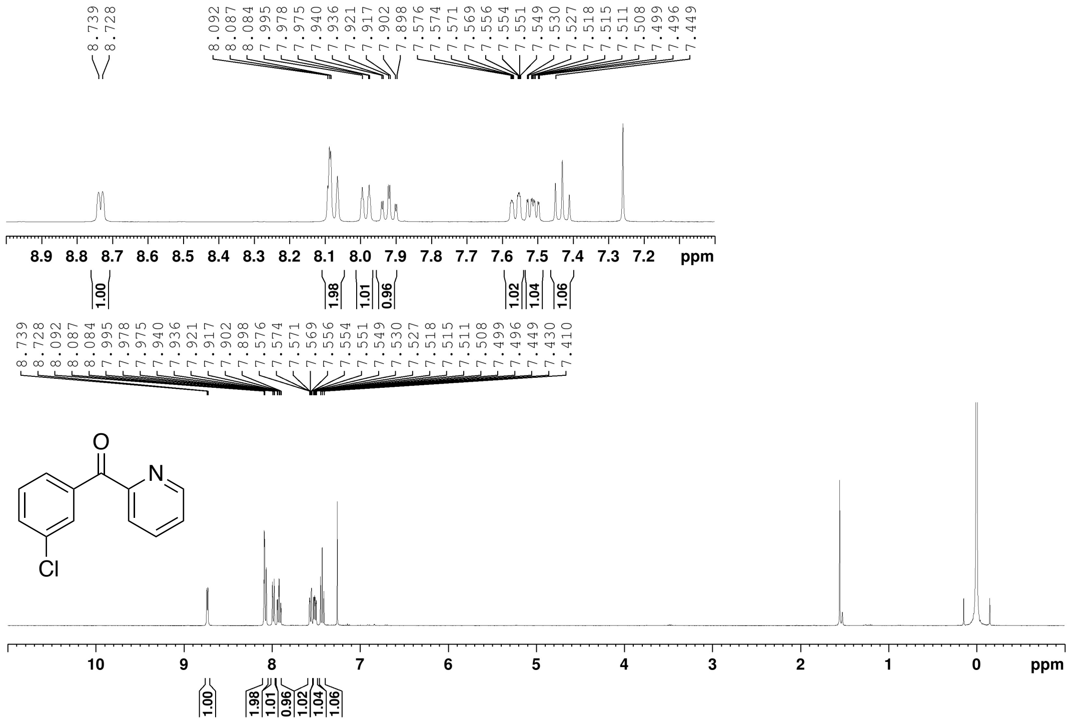

^{1}H-NMR of **17a**

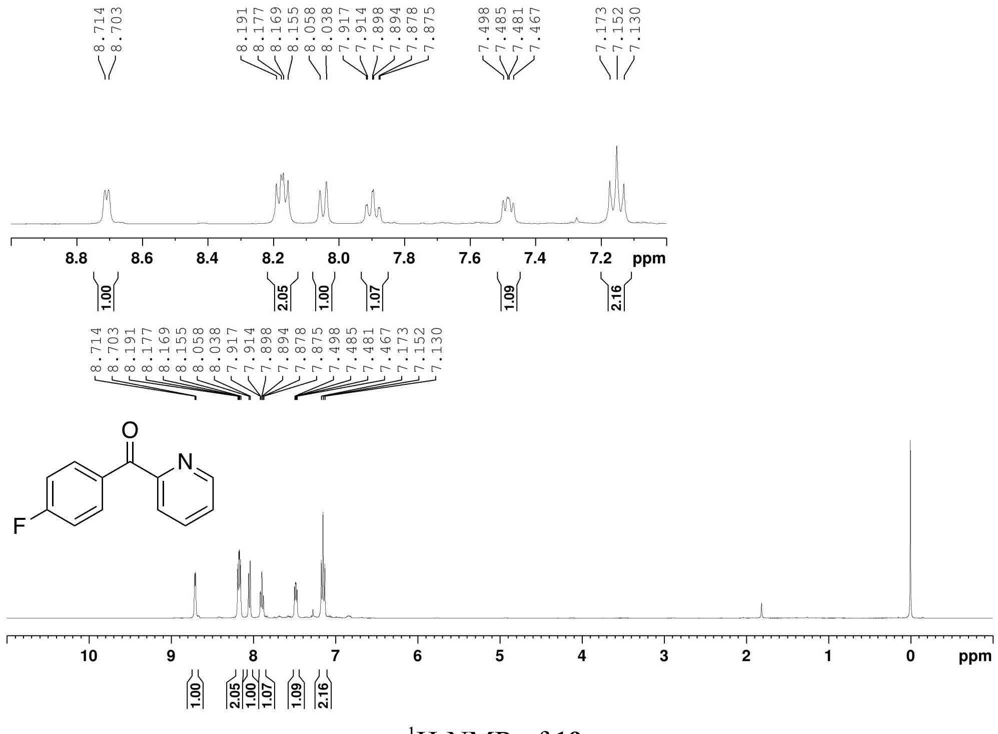

^{1}H-NMR of **19a**

2. ¹H-NMR of racemic alcohol **3b-5b** and **12b-19b**

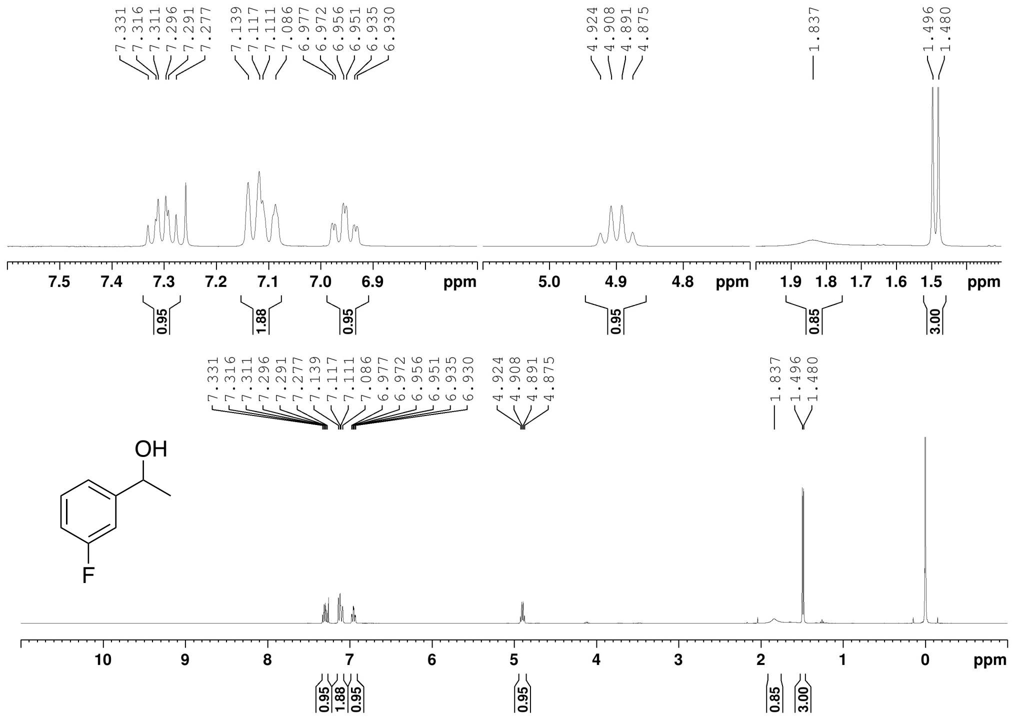

¹H-NMR of *rac*-**3b**

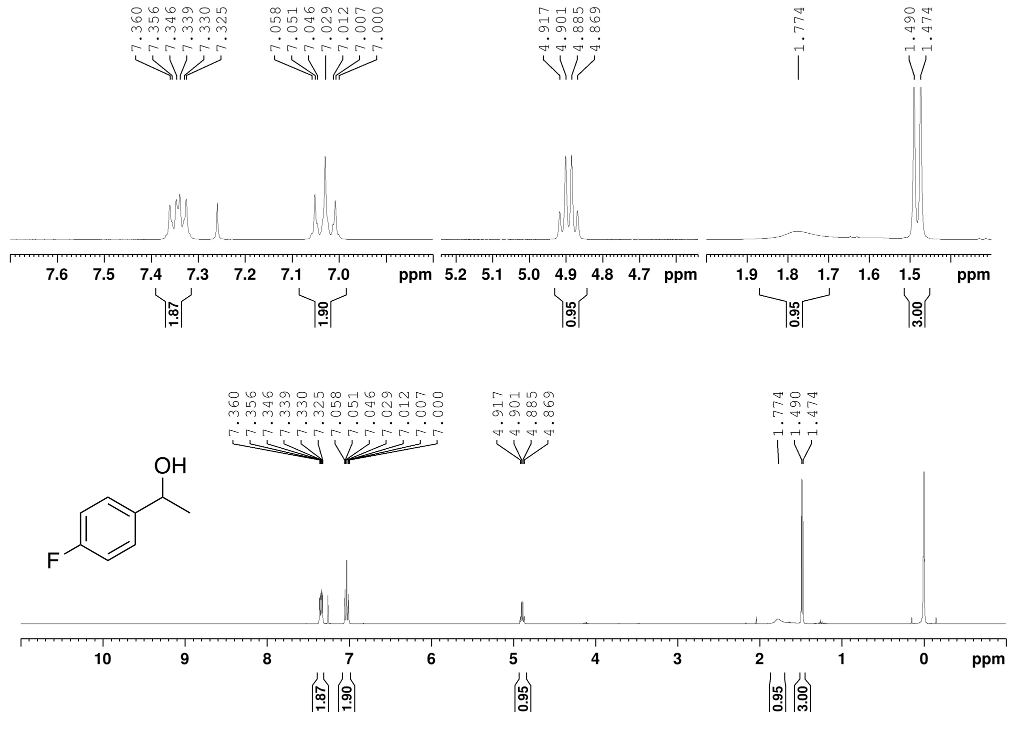

¹H-NMR of *rac*-**4b**

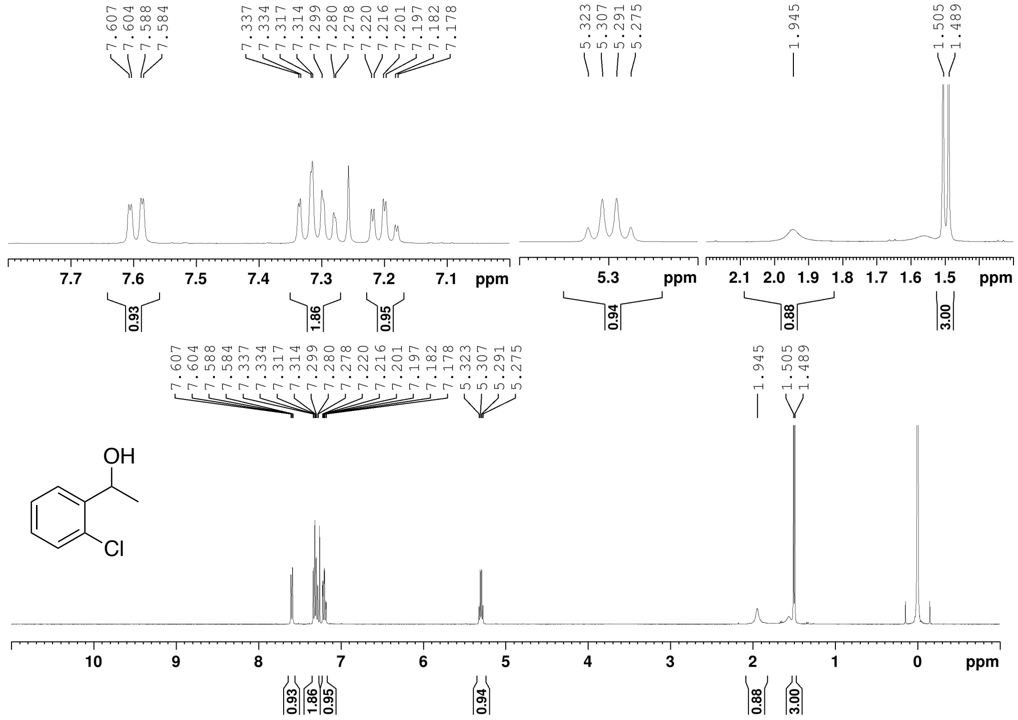

^{1}H-NMR of *rac*-**5b**

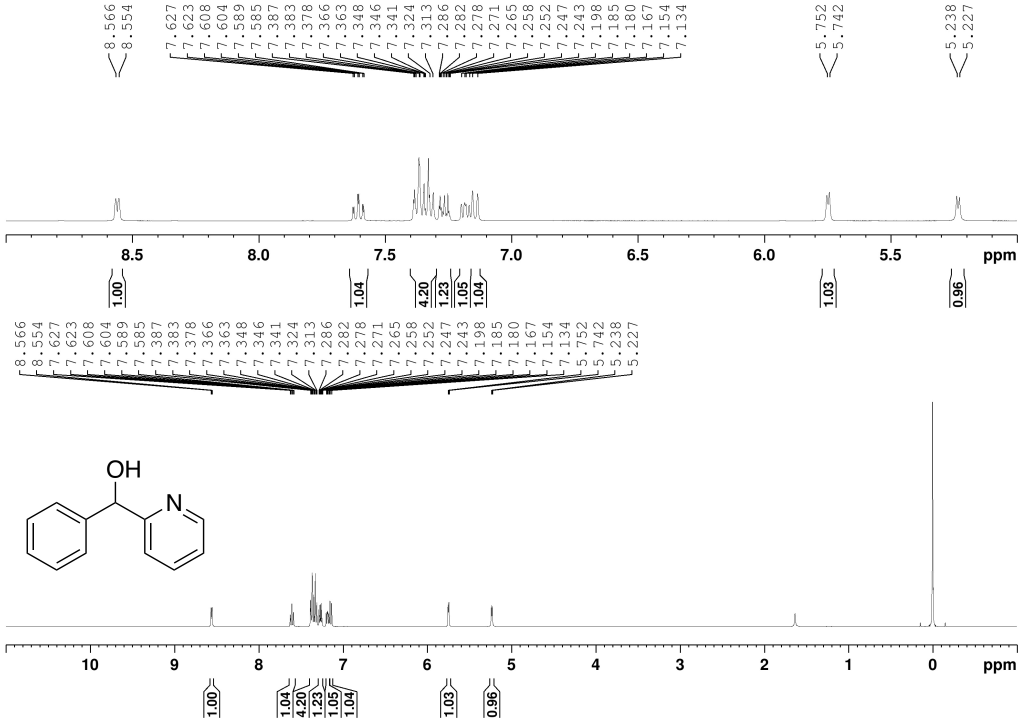

^{1}H-NMR of *rac*-**12b**

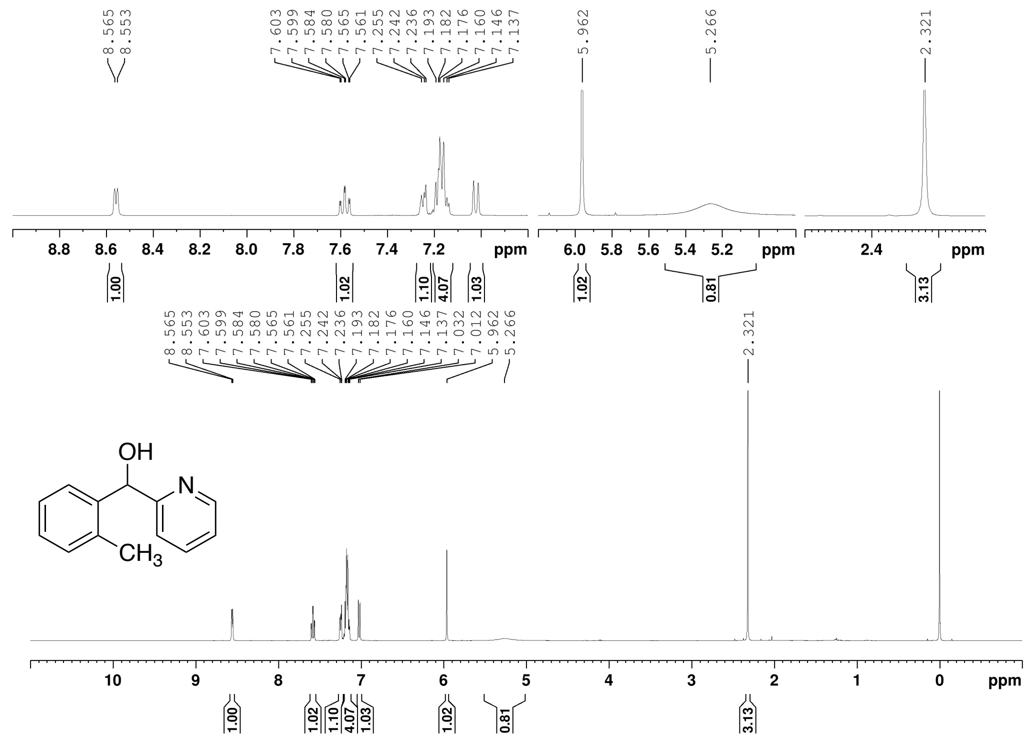

^{1}H-NMR of *rac*-**13b**

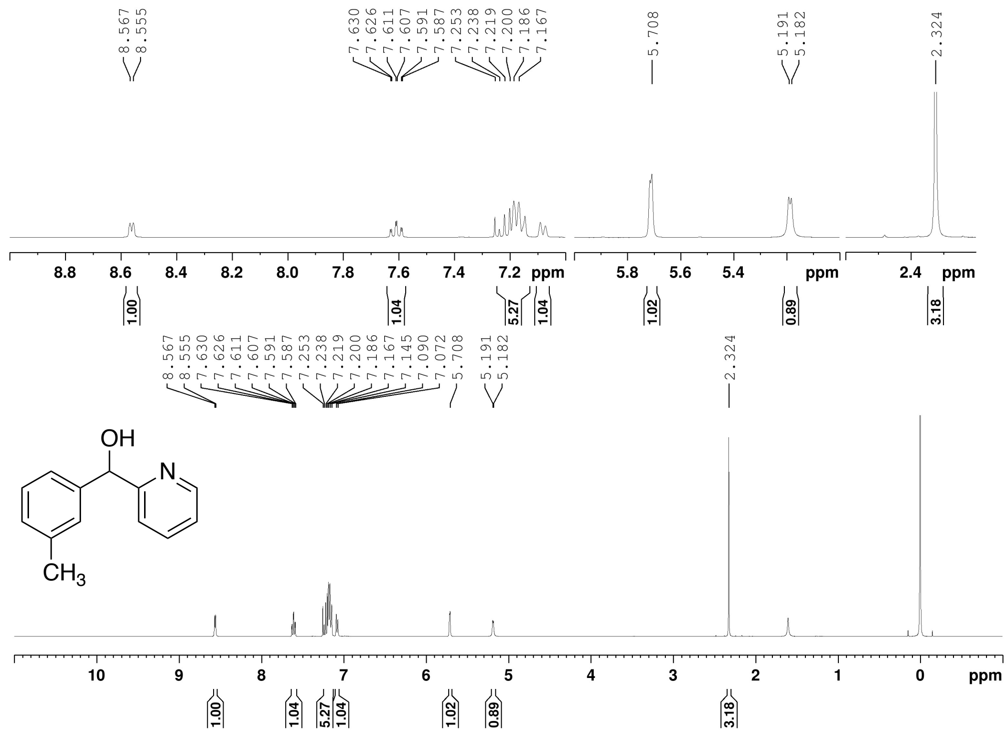

^{1}H-NMR of *rac*-**14b**

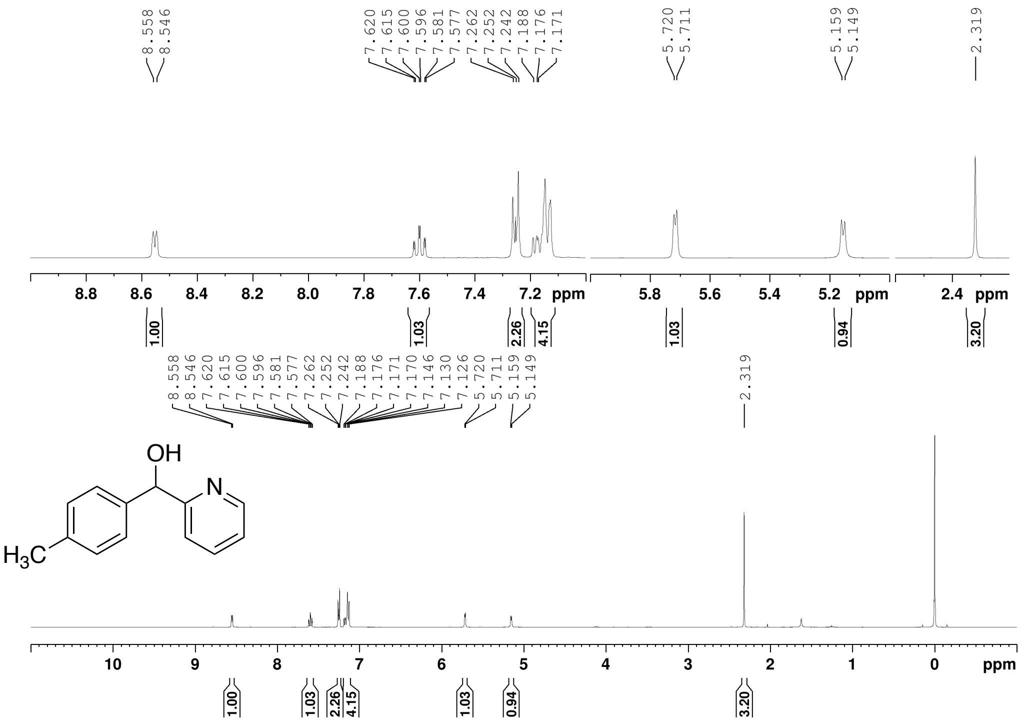

^{1}H-NMR of *rac*-**15b**

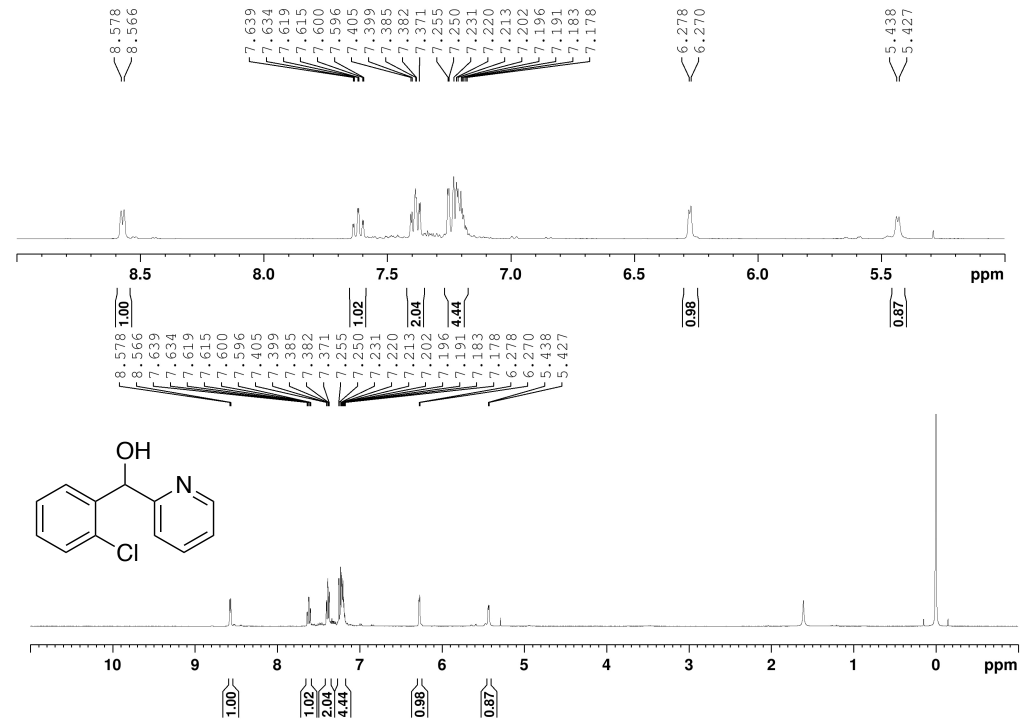

^{1}H-NMR of *rac*-**16b**

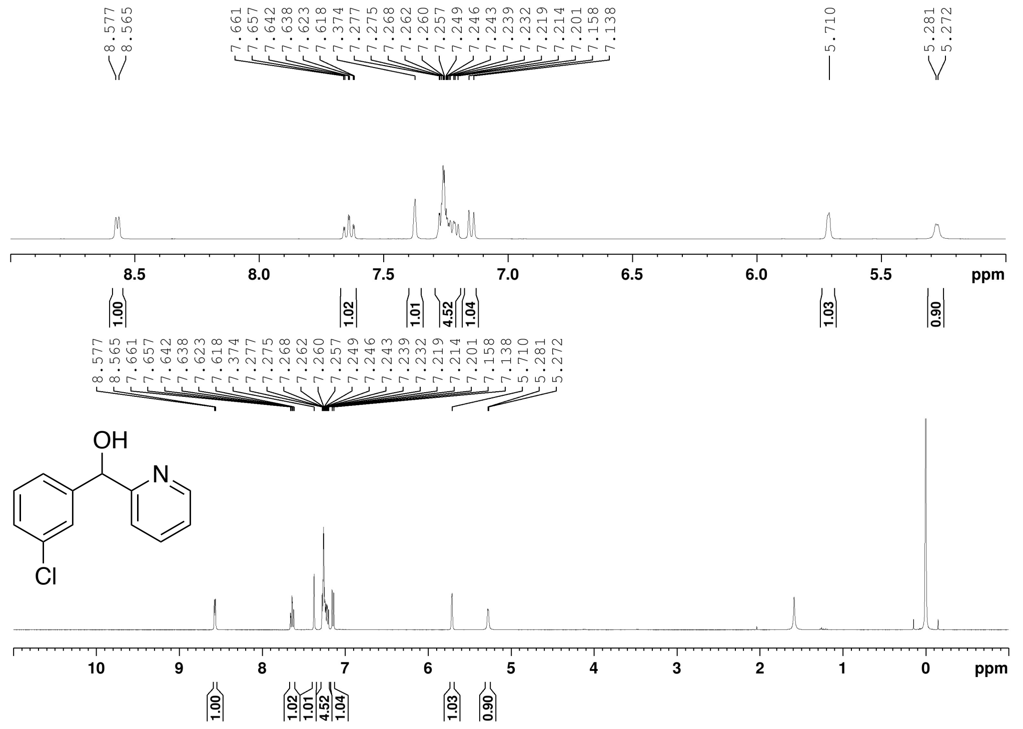

^{1}H-NMR of *rac*-**17b**

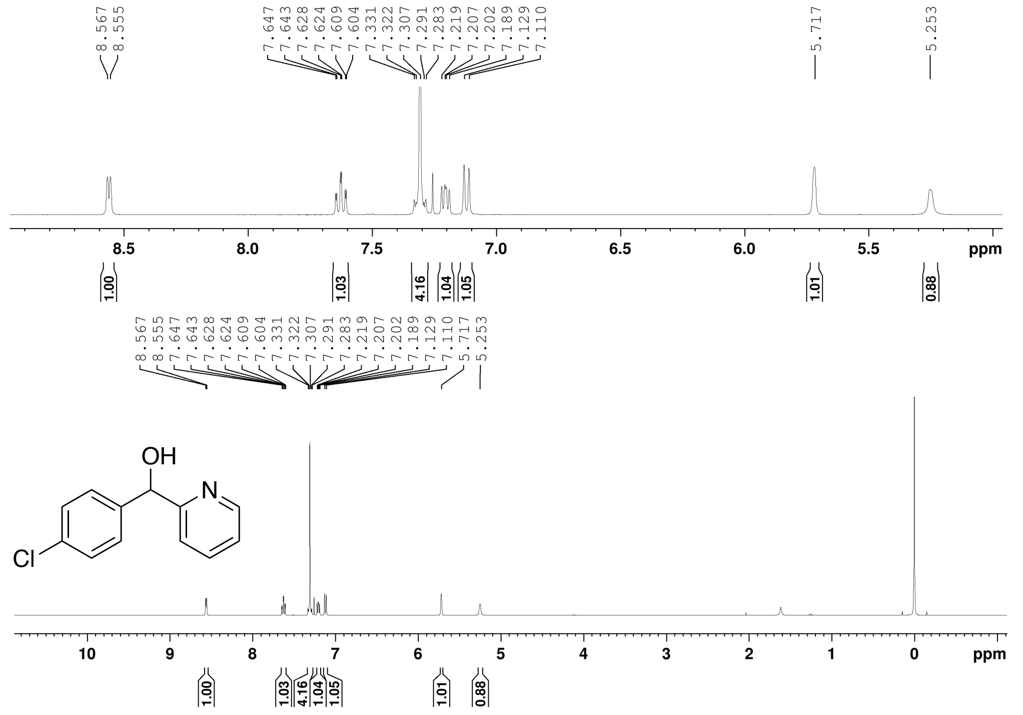

^{1}H-NMR of *rac*-**18b**

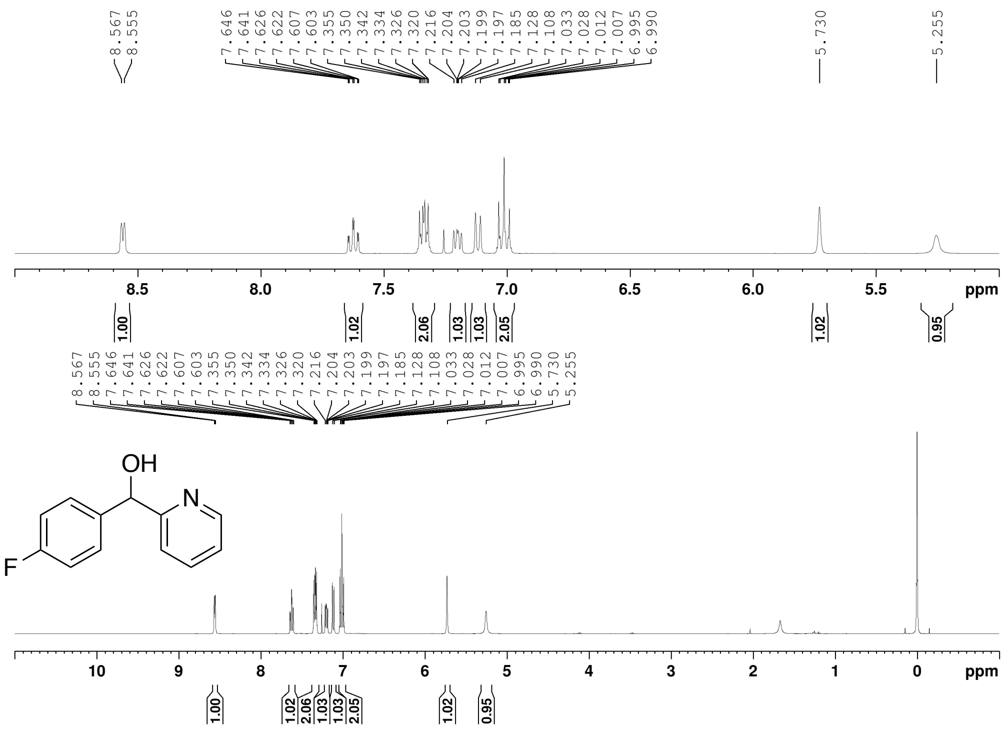

^{1}H-NMR of *rac*-**19b**

3. GC results of racemic alcohol **1b-11b** and Phe56Ile catalyzed analytical scale asymmetric reduction of **1a-11a** (The GC samples also contain internal standard (IS) and/or corresponding ketone.)

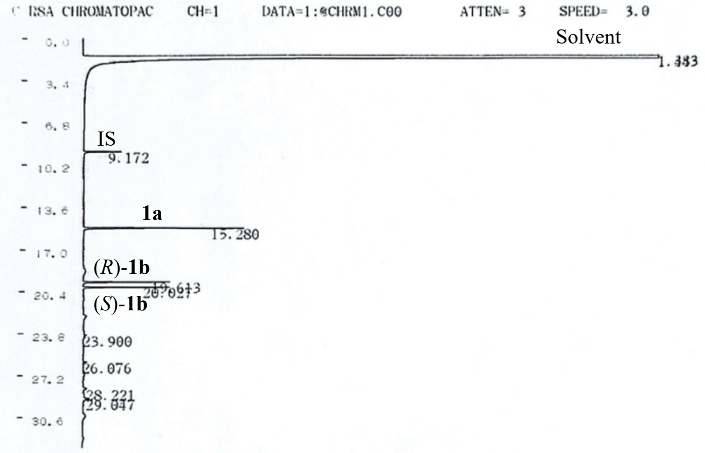

C-RSA CHROMATOPAC CH=1 Report No.=5 DATA=1:@CHRM1.C00 22/06/24 16:31:52

** CALCULATION REPORT **

CH	PKNO	TIME	AREA	HEIGHT	MK	IDNO	CONC	NAME
1	1	1.383	3488396	1109808	E		38.5159	
	2	1.443	5549811	1099301	VE		61.2763	
	3	9.172	1865	521			0.0206	
	4	15.28	7850	2210			0.0867	
	5	19.613	3922	1178			0.0433	
	6	20.027	3939	1179			0.0435	
	7	23.9	163	32			0.0018	
	8	26.076	215	50			0.0024	
	9	28.221	372	43			0.0041	
	10	29.047	489	70			0.0054	
		TOTAL	9057020	2214392			100	

rac-**1b**

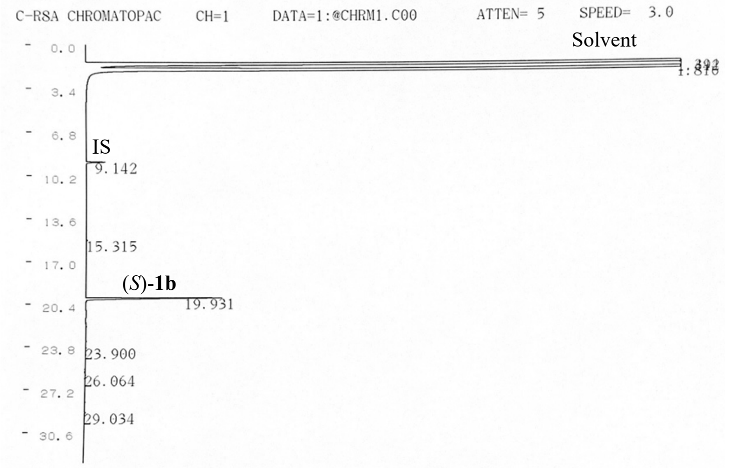

C-RSA CHROMATOPAC CH=1 Report No.=2 DATA=1:@CHRM1.C00 22/06/26 11:37:12

** CALCULATION REPORT **

CH	PKNO	TIME	AREA	HEIGHT	MK	IDNO	CONC	NAME
1	1	1.394	3306863	1100118	E		31.5546	
	2	1.442	5463560	1097247	VE		52.1341	
	3	1.816	1665643	267852	V		15.8938	
	4	9.142	4121	1037			0.0393	
	5	15.315	331	100			0.0032	
	6	19.931	38530	7292			0.3677	
	7	23.9	139	28			0.0013	
	8	26.064	279	66			0.0027	
	9	29.034	356	50			0.0034	
		TOTAL	10479818	2473789			100	

Phe56Ile catalyzed analytical scale asymmetric reduction of **1a**

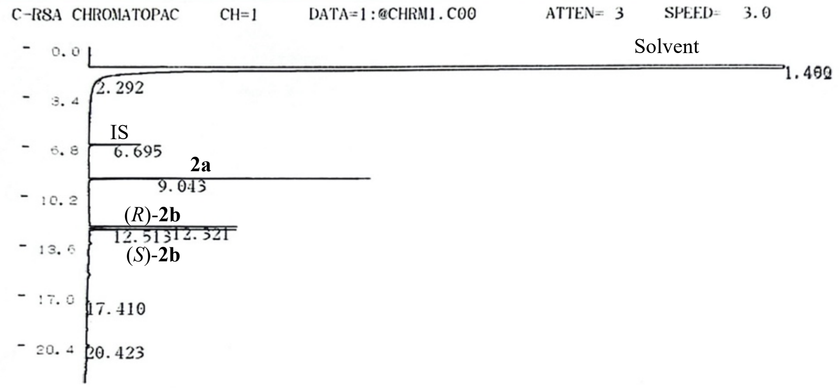

C-R8A CHROMATOPAC CH=1 Report No.=4 DATA=1:@CHRM1.C00 22/07/03 12:05:30

** CALCULATION REPORT **

CH	PKNO	TIME	AREA	HEIGHT	MK	IDNO	CONC	NAME
1	1	1.4	3243635	1101898	E		36.5674	
	2	1.452	5609651	1097959	SVE		63.2409	
	4	6.695	1417	620			0.016	
	5	9.043	7389	3260			0.0833	
	6	12.321	3976	1720			0.0448	
	7	12.513	3873	1703	V		0.0437	
	8	17.41	133	30			0.0015	
	9	20.423	210	33			0.0024	
	TOTAL		8870281	2207223			100	

rac-**2b**

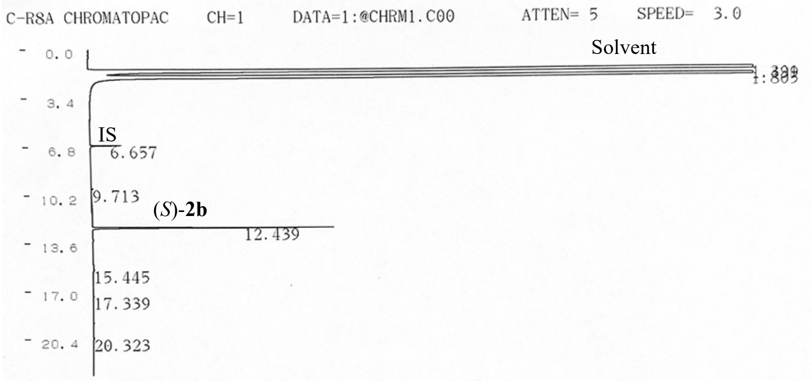

C-R8A CHROMATOPAC CH=1 Report No.=14 DATA=1:@CHRM1.C00 22/06/25 19:55:24

** CALCULATION REPORT **

CH	PKNO	TIME	AREA	HEIGHT	MK	IDNO	CONC	NAME
1	1	1.391	3523856	1100359	E		34.2756	
	2	1.439	5175177	1097559	VE		50.3376	
	3	1.805	1539686	265445	V		14.9761	
	4	6.657	3736	1513			0.0363	
	5	9.713	220	103			0.0021	
	6	12.439	37526	11775			0.365	
	7	15.445	299	39			0.0029	
	8	17.339	157	35			0.0015	
	9	20.323	285	40			0.0028	
	TOTAL		10280937	2476865			100	

Phe56Ile catalyzed analytical scale asymmetric reduction of **2a**

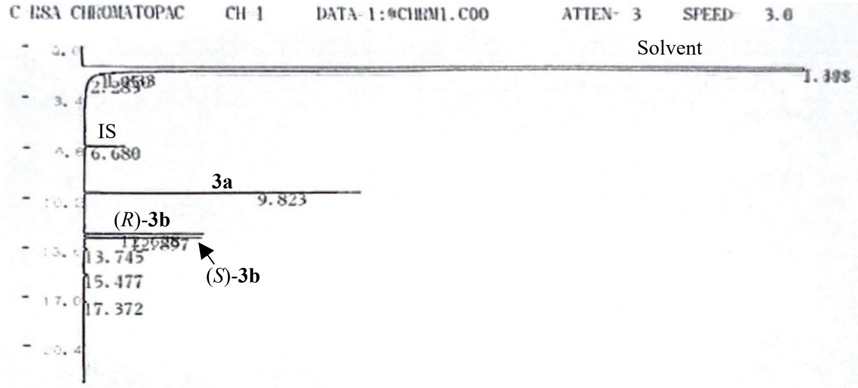

C-RSA CHROMATOPAC CH=1 Report No.=4 DATA=1:@CHRM1.C00 22/06/16 15:48:18

** CALCULATION REPORT **

CH	PKNO	TIME	AREA	HEIGHT	MK	IDNO	CONC	NAME
1	1	1.391	3620903	1108389	E		40.7305	
	2	1.448	5253540	1099336	SVE		59.0956	
	3	1.833	136	70	T		0.0015	
	4	1.95	115	70	T		0.0013	
	6	6.68	1050	458			0.0118	
	7	9.823	6806	3163			0.0766	
	8	12.628	3366	1370			0.0379	
	9	12.897	3347	1345	V		0.0377	
	10	13.745	246	33			0.0028	
	11	15.477	247	40			0.0028	
	12	17.372	145	33			0.0016	
		TOTAL	8889894	2214306			100	

rac-**3b**

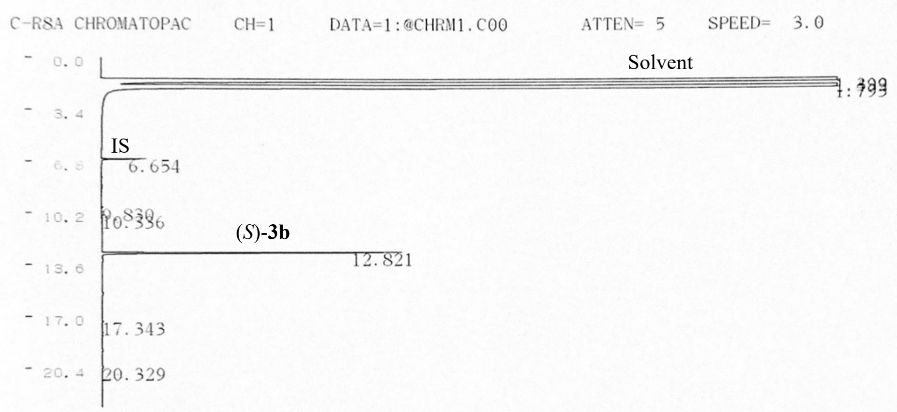

C-RSA CHROMATOPAC CH=1 Report No.=15 DATA=1:@CHRM1.C00 22/06/25 20:26:08

** CALCULATION REPORT **

CH	PKNO	TIME	AREA	HEIGHT	MK	IDNO	CONC	NAME
1	1	1.392	3489145	1100396	E		33.1812	
	2	1.439	5188832	1097500	VE		49.345	
	3	1.793	1781668	296389	V		16.9434	
	4	6.654	4873	1938			0.0463	
	5	9.83	146	62			0.0014	
	6	10.336	193	88			0.0018	
	7	12.821	50041	12969			0.4759	
	8	17.343	237	55			0.0022	
	9	20.329	288	41			0.0027	
		TOTAL	10515419	2509438			100	

Phe56Ile catalyzed analytical scale asymmetric reduction of **3a**

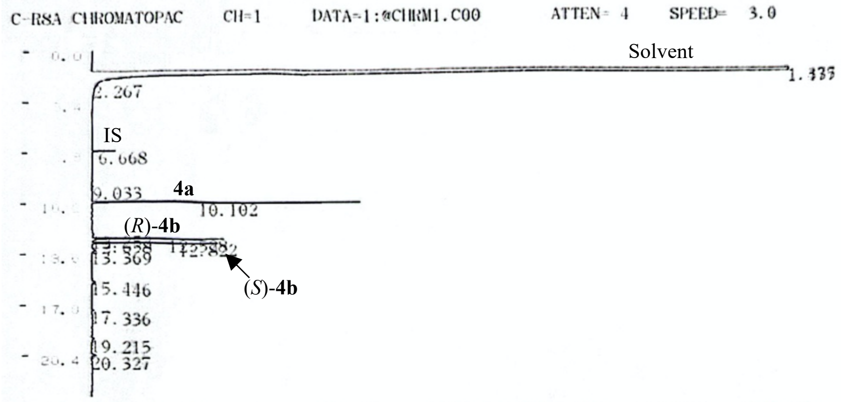

C-R8A CHROMATOPAC CH=1 Report No.=4 DATA=1:@CHRM1.C00 22/06/22 12:00:40

** CALCULATION REPORT **

CH	PKNO	TIME	AREA	HEIGHT	MK	IDNO	CONC	NAME
1	1	1.375	3331686	1106704	E		37.0228	
	2	1.437	5632230	1099251	SVE		62.5873	
	4	6.668	1300	554			0.0144	
	5	9.033	64	28			0.0007	
	6	10.102	15499	6174			0.1722	
	7	12.252	283	32			0.0031	
	8	12.528	7407	2981	V		0.0823	
	9	12.658	255	57	V		0.0028	
	10	12.822	7279	2835	V		0.0809	
	11	13.369	728	58			0.0081	
	12	15.446	651	85			0.0072	
	13	17.336	297	68			0.0033	
	14	19.215	534	69			0.0059	
	15	20.327	791	111			0.0088	
		TOTAL	8998999	2219004			100	

rac-**4b**

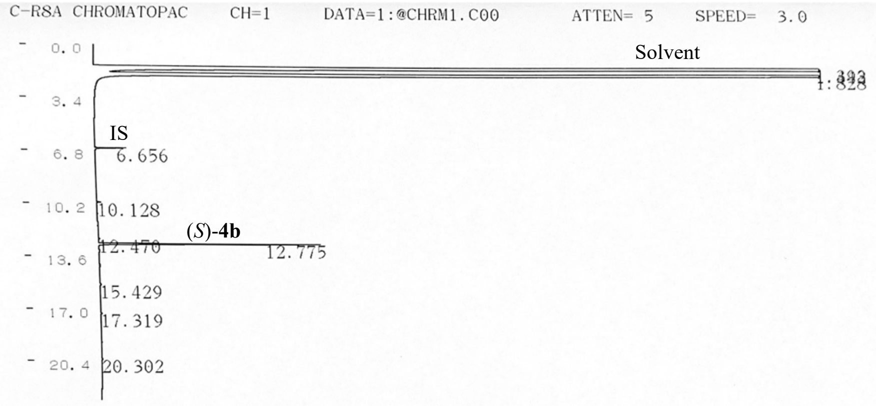

C-R8A CHROMATOPAC CH=1 Report No.=1 DATA=1:@CHRM1.C00 22/06/26 11:06:40

** CALCULATION REPORT **

CH	PKNO	TIME	AREA	HEIGHT	MK	IDNO	CONC	NAME
1	1	1.393	3362026	1100043	E		34.4635	
	2	1.442	5221543	1097225	VE		53.5251	
	3	1.828	1132617	207623	V		11.6102	
	4	6.656	3294	1379			0.0338	
	5	10.128	470	206			0.0048	
	6	12.47	171	74			0.0018	
	7	12.775	34226	9809			0.3508	
	8	15.429	299	44			0.0031	
	9	17.319	339	77			0.0035	
	10	20.302	341	47			0.0035	
		TOTAL	9755321	2416527			100	

Phe56Ile catalyzed analytical scale asymmetric reduction of **4a**

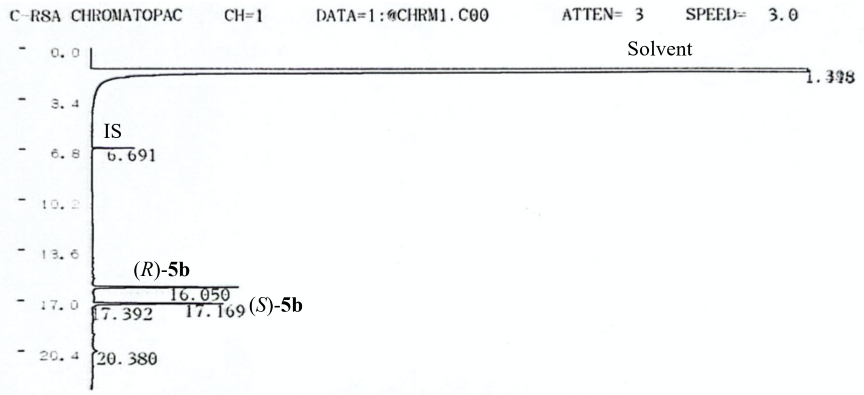

C-R8A CHROMATOPAC CH=1 Report No.=8 DATA=1:@CHRM1.C00 22/06/28 15:27:14

** CALCULATION REPORT **

CH	PKNO	TIME	AREA	HEIGHT	MK	IDNO	CONC	NAME
1	1	1.396	2990879	1101350	E		34.0569	
	2	1.448	5775301	1097609	VE		65.7629	
	3	6.691	1119	478			0.0127	
	4	16.05	7136	1609			0.0813	
	5	17.169	7129	1437			0.0812	
	6	17.392	141	29	V		0.0016	
	7	20.38	299	43			0.0034	
	TOTAL		8782001	2202554			100	

rac-**5b**

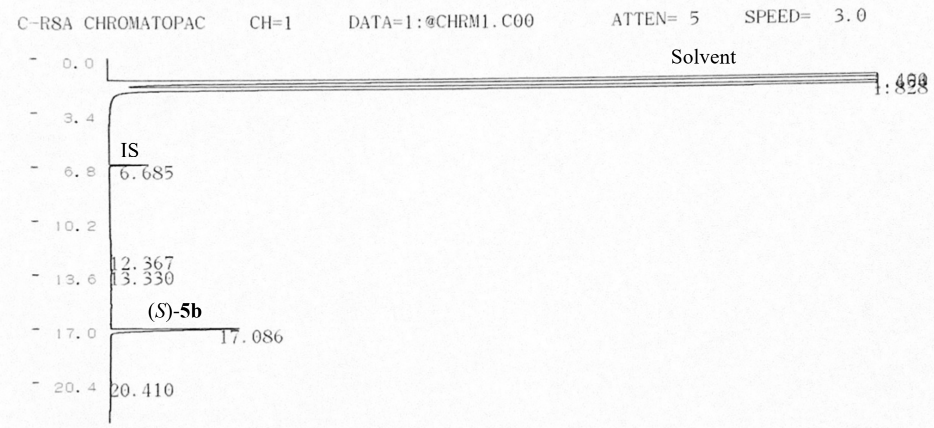

C-R8A CHROMATOPAC CH=1 Report No.=2 DATA=1:@CHRM1.C00 22/07/02 13:38:18

** CALCULATION REPORT **

CH	PKNO	TIME	AREA	HEIGHT	MK	IDNO	CONC	NAME
1	1	1.4	3101560	1105209	E		30.1936	
	2	1.454	5655978	1098037	VE		55.0608	
	3	1.828	1469563	253364	V		14.3061	
	4	6.685	4167	1633			0.0406	
	5	12.367	157	32			0.0015	
	6	13.33	182	71			0.0018	
	7	17.086	40262	5407			0.3919	
	8	20.41	379	47			0.0037	
	TOTAL		10272243	2463799			100	

Phe56Ile catalyzed analytical scale asymmetric reduction of **5a**

C-R8A CHROMATOPAC CH=1 DATA=1:@CHRM1.C00 ATTEN= 3 SPEED= 3.0

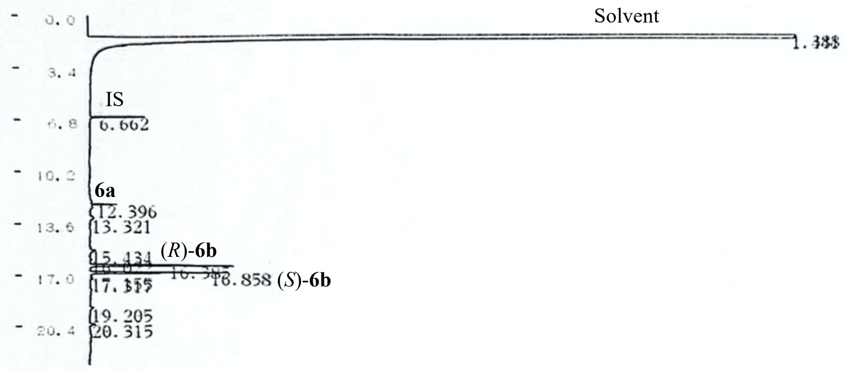

C-R8A CHROMATOPAC CH=1 Report No.=1 DATA=1:@CHRM1.C00 22/06/25 12:06:16

** CALCULATION REPORT **

CH	PKNO	TIME	AREA	HEIGHT	MK	IDNO	CONC	NAME
1	1	1.388	3466588	1102080	E		38.8725	
	2	1.441	5432206	1097897	VE		60.914	
	3	6.662	1408	619			0.0158	
	4	12.396	649	290			0.0073	
	5	13.321	452	37			0.0051	
	6	15.434	435	61			0.0049	
	7	16.022	102	25			0.0011	
	8	16.385	7470	1593			0.0838	
	9	16.858	7529	1514			0.0844	
	10	17.155	124	24	V		0.0014	
	11	17.317	172	35	V		0.0019	
	12	19.205	264	38			0.003	
	13	20.315	434	63			0.0049	
		TOTAL	8917830	2204275			100	

rac-**6b**

C-R8A CHROMATOPAC CH=1 DATA=1:@CHRM1.C00 ATTEN= 5 SPEED= 3.0

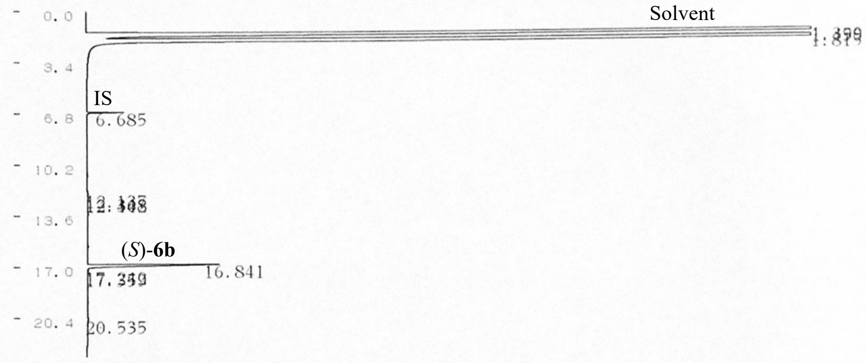

C-R8A CHROMATOPAC CH=1 Report No.=3 DATA=1:@CHRM1.C00 22/07/02 14:09:24

** CALCULATION REPORT **

CH	PKNO	TIME	AREA	HEIGHT	MK	IDNO	CONC	NAME
1	1	1.399	3271720	1105525	E		31.0946	
	2	1.45	5513380	1097976	VE		52.3994	
	3	1.815	1690926	285260	V		16.0706	
	4	6.685	4225	1646			0.0402	
	5	12.137	152	63			0.0014	
	6	12.368	170	42	V		0.0016	
	7	12.442	227	74	V		0.0022	
	8	16.841	40242	5874	S		0.3825	
	9	17.242	110	26	T		0.001	
	10	17.359	123	29	TV		0.0012	
	11	20.535	567	46			0.0054	
		TOTAL	10521836	2496561			100	

Phe56Ile catalyzed analytical scale asymmetric reduction of **6a**

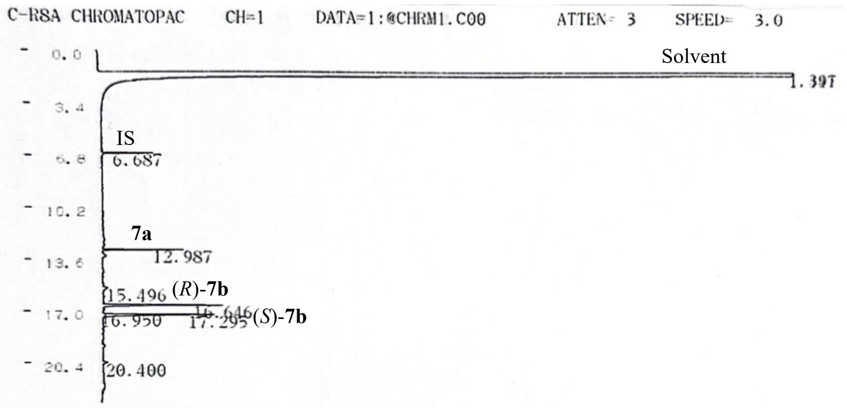

C-RSA CHROMATOPAC CH=1 Report No.=2 DATA=1:@CHRM1.C00 22/06/30 12:46:08

** CALCULATION REPORT **

CH	PKNO	TIME	AREA	HEIGHT	MK	IDNO	CONC	NAME
1	1	1.391	3596594	1108494	E		40.2468	
	2	1.447	5322315	1099198	VE		59.5581	
	3	6.687	1362	588			0.0152	
	4	12.987	2262	904			0.0253	
	5	15.496	403	48			0.0045	
	6	16.646	6386	1343			0.0715	
	7	16.95	88	18	V		0.001	
	8	17.295	6588	1244	V		0.0737	
	9	20.4	344	48			0.0038	
	TOTAL		8936339	2211884			100	

rac-**7b**

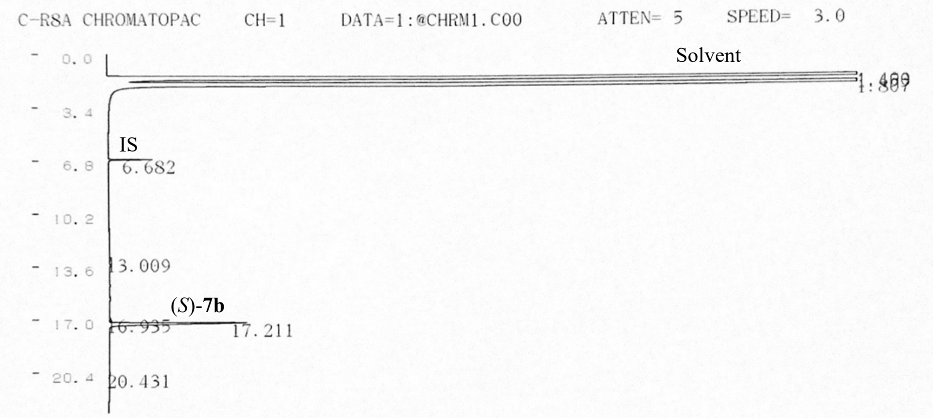

C-RSA CHROMATOPAC CH=1 Report No.=4 DATA=1:@CHRM1.C00 22/07/02 14:40:38

** CALCULATION REPORT **

CH	PKNO	TIME	AREA	HEIGHT	MK	IDNO	CONC	NAME
1	1	1.4	3026293	1104396	E		28.5318	
	2	1.453	5634816	1098064	VE		53.1249	
	3	1.807	1896386	313378	V		17.8791	
	4	6.682	4827	1887			0.0455	
	5	13.009	265	102			0.0025	
	6	16.935	255	54			0.0024	
	7	17.211	43586	5834			0.4109	
	8	20.431	317	40			0.003	
	TOTAL		10606742	2523755			100	

Phe56Ile catalyzed analytical scale asymmetric reduction of **7a**

C-R8A CHROMATOPAC CH=1 DATA=1:@CHRM1.C00 ATTEN= 3 SPEED= 3.0

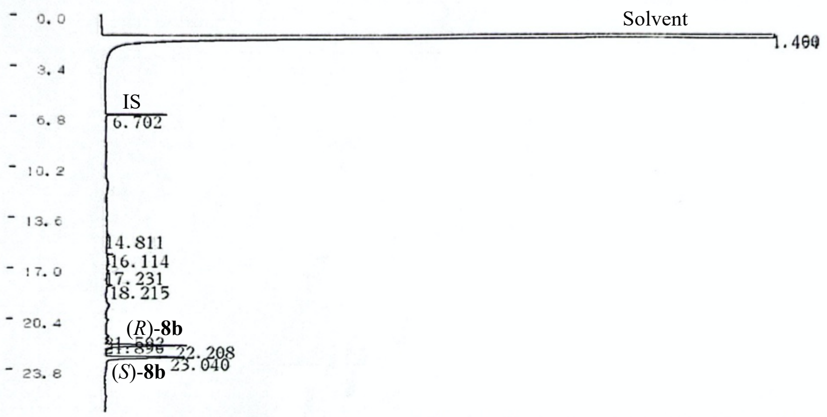

C-R8A CHROMATOPAC CH=1 Report No.=7 DATA=1:@CHRM1.C00 22/06/30 15:32:44

** CALCULATION REPORT **

CH	PKNO	TIME	AREA	HEIGHT	MK	IDNO	CONC	NAME
1	1	1.4	3578578	1108460	E		40.1615	
	2	1.454	5314999	1099361	VE		59.6489	
	3	6.702	1592	703			0.0179	
	4	14.811	124	30			0.0014	
	5	16.114	271	80			0.003	
	6	17.231	113	23			0.0013	
	7	18.215	326	63			0.0037	
	8	21.592	172	28			0.0019	
	9	21.896	200	28			0.0022	
	10	22.208	6830	908			0.0767	
	11	23.04	7276	885			0.0817	
	TOTAL		8910479	2210568			100	

rac-**8b**

C-R8A CHROMATOPAC CH=1 DATA=1:@CHRM1.C00 ATTEN= 5 SPEED= 3.0

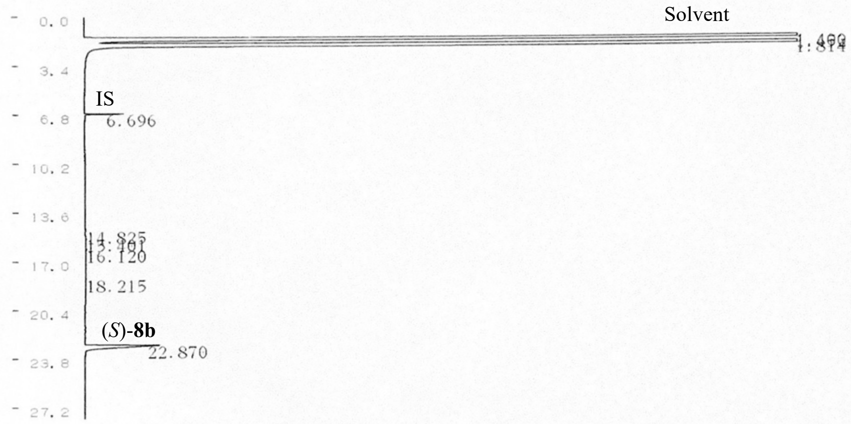

C-R8A CHROMATOPAC CH=1 Report No.=14 DATA=1:@CHRM1.C00 22/07/02 20:30:08

** CALCULATION REPORT **

CH	PKNO	TIME	AREA	HEIGHT	MK	IDNO	CONC	NAME
1	1	1.4	3065857	1105921	E		29.2787	
	2	1.452	5583536	1098380	VE		53.3224	
	3	1.814	1779272	296151	V		16.9919	
	4	6.696	4394	1769			0.042	
	5	14.825	90	21			0.0009	
	6	15.401	86	24			0.0008	
	7	16.12	288	83			0.0027	
	8	18.215	209	41			0.002	
	9	22.87	37550	3359			0.3586	
	TOTAL		10471278	2505749			100	

Phe56Ile catalyzed analytical scale asymmetric reduction of **8a**

C-RSA CHROMATOPAC CH=1 DATA=1:@CHRM1.C00 ATTEN= 3 SPEED= 3.0

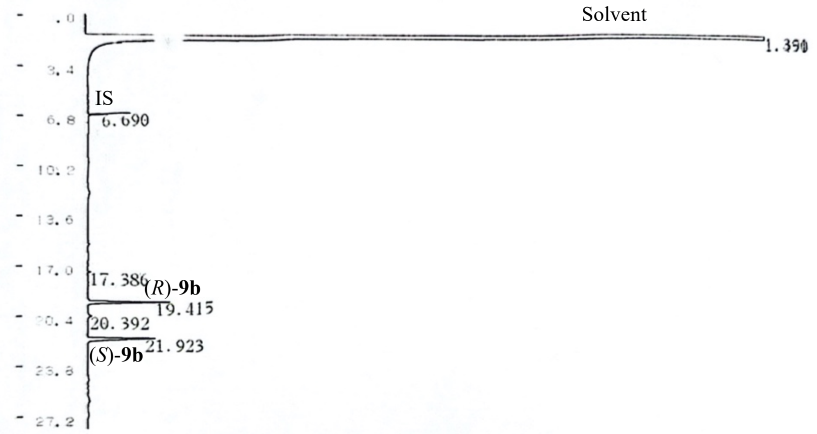

C-RSA CHROMATOPAC CH=1 Report No.=11 DATA=1:@CHRM1.C00 22/06/29 16:51:24

** CALCULATION REPORT **

CH	PKNO	TIME	AREA	HEIGHT	MK	IDNO	CONC	NAME
1	1	1.391	3438258	1107367	E		38.9632	
	2	1.45	5372635	1098512	VE		60.8841	
	3	6.69	1107	495			0.0125	
	4	17.386	168	37			0.0019	
	5	19.415	5959	918			0.0675	
	6	20.392	215	32			0.0024	
	7	21.923	6028	766			0.0683	
		TOTAL	8824368	2208127			100	

rac-**9b**

C-R8A CHROMATOPAC CH=1 DATA=1:@CHRM1.C00 ATTEN= 5 SPEED= 3.0

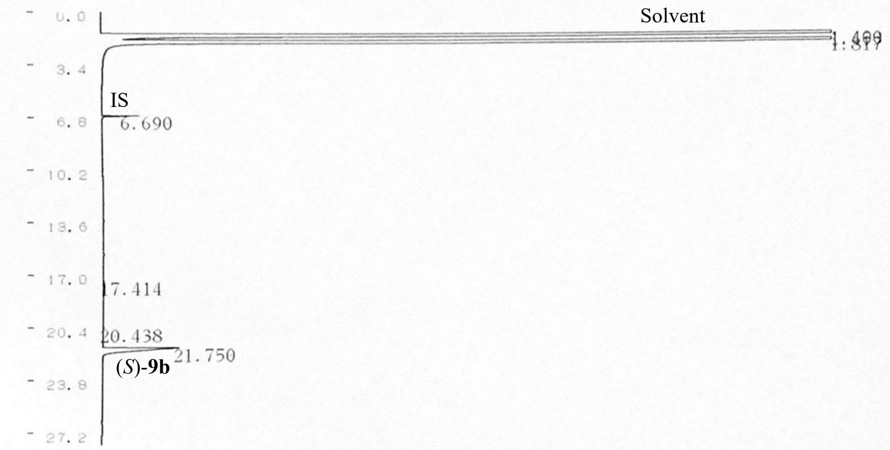

C-R8A CHROMATOPAC CH=1 Report No.=5 DATA=1:@CHRM1.C00 22/07/02 15:11:36

** CALCULATION REPORT **

CH	PKNO	TIME	AREA	HEIGHT	MK	IDNO	CONC	NAME
1	1	1.4	3040296	1104896	E		29.1595	
	2	1.453	5649048	1098134	VE		54.1801	
	3	1.817	1691934	281531	V		16.2274	
	4	6.69	4082	1644			0.0392	
	5	17.414	198	47			0.0019	
	6	20.438	218	33			0.0021	
	7	21.75	40649	3324			0.3899	
		TOTAL	10426422	2489608			100	

Phe56Ile catalyzed analytical scale asymmetric reduction of **9a**

-RSA CHROMATOPAC CH=1 DATA=1:@CHRM1.C00 ATTEN= 3 SPEED= 3.0

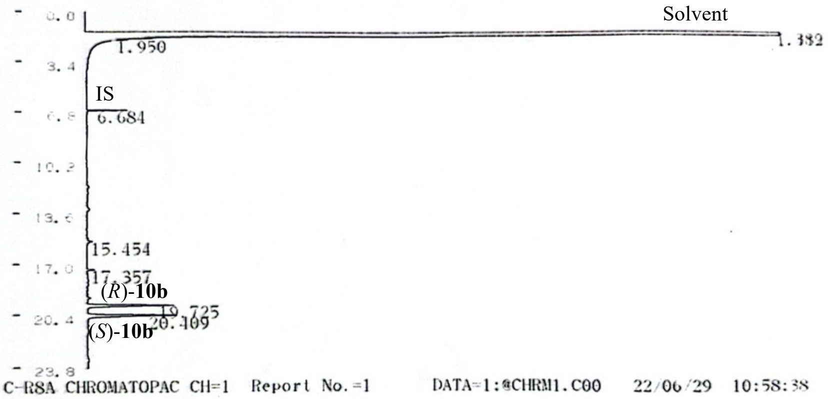

C-RSA CHROMATOPAC CH=1 Report No.=1 DATA=1:@CHRM1.C00 22/06/29 10:58:38

** CALCULATION REPORT **

CH	PKNO	TIME	AREA	HEIGHT	MK	IDNO	CONC	NAME
1	1	1.389	3442404	1101771	E		38.7296	
	2	1.442	5430454	1097781	SVE		61.0966	
	3	1.95	185	142	T		0.0021	
	4	6.684	1123	479			0.0126	
	5	15.454	369	53			0.0042	
	6	17.357	391	86			0.0044	
	7	19.725	6414	952			0.0722	
	8	20.409	6974	962			0.0785	
		TOTAL	8888312	2202225			100	

rac-**10b**

C-RSA CHROMATOPAC CH=1 DATA=1:@CHRM1.C00 ATTEN= 5 SPEED= 3.0

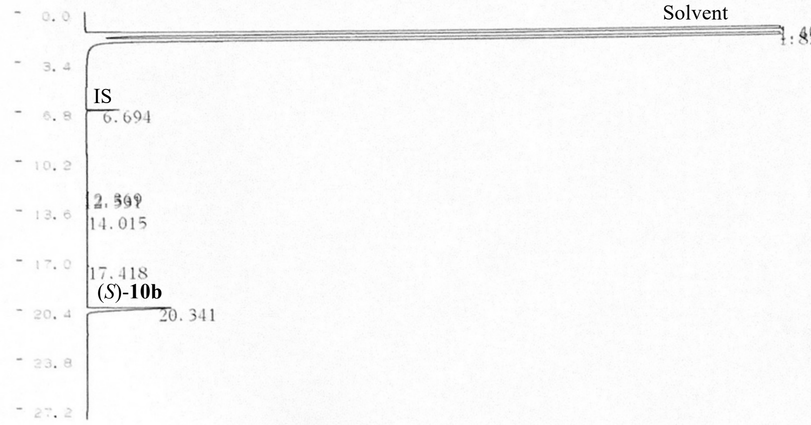

C-RSA CHROMATOPAC CH=1 Report No.=6 DATA=1:@CHRM1.C00 22/07/02 15:47:42

** CALCULATION REPORT **

CH	PKNO	TIME	AREA	HEIGHT	MK	IDNO	CONC	NAME
1	1	1.4	3196514	1105967	E		31.048	
	2	1.452	5535036	1098112	VE		53.7622	
	3	1.824	1520261	260915	V		14.7664	
	4	6.694	3861	1551			0.0375	
	5	12.369	203	67			0.002	
	7	14.015	113	38			0.0011	
	8	17.418	244	55			0.0024	
	9	20.341	39171	3881			0.3805	
		TOTAL	10295399	2470585			100	

Phe56Ile catalyzed analytical scale asymmetric reduction of **10a**

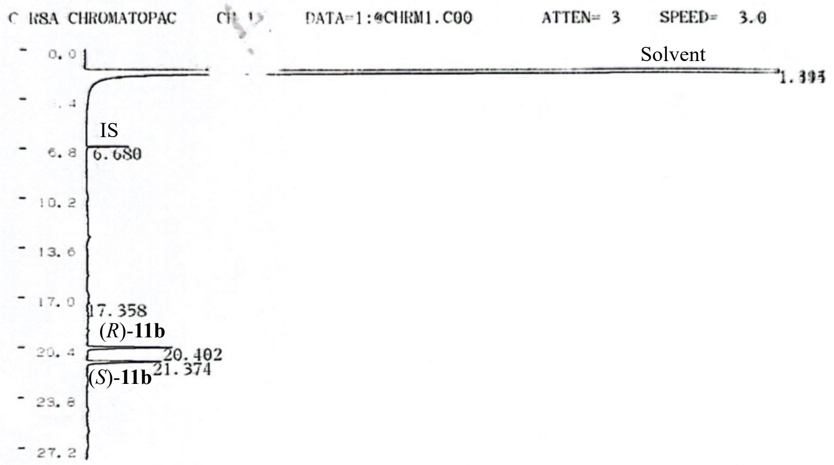

C-RSA CHROMATOPAC CH=1 Report No.=4 DATA=1:@CHRM1.C00 22/06/29 12:40:22

** CALCULATION REPORT **

CH	PKNO	TIME	AREA	HEIGHT	MK	IDNO	CONC	NAME
1	1	1.394	3107209	1102185	E		35.2688	
	2	1.445	5688086	1097720	VE		64.5633	
	3	6.68	1096	495			0.0124	
	4	17.358	117	26			0.0013	
	5	20.402	6961	961			0.079	
	6	21.374	6623	840			0.0752	
	TOTAL		8810088	2202225			100	

rac-**11b**

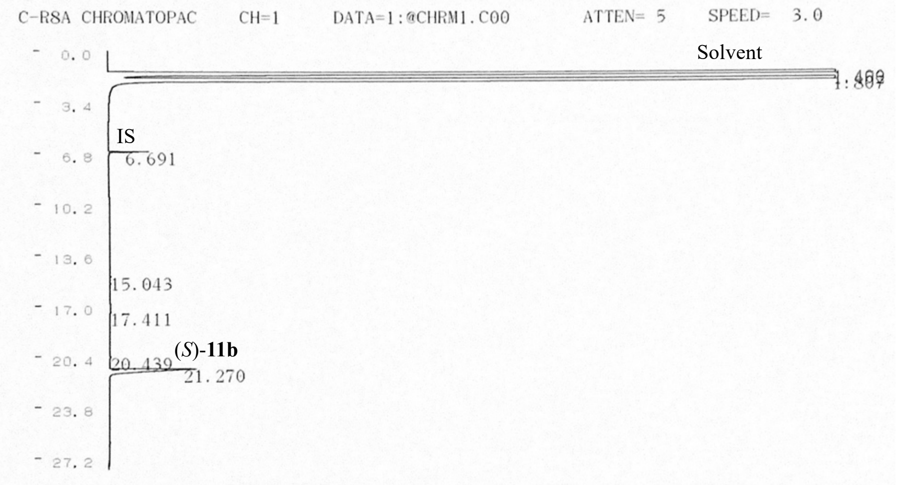

C-RSA CHROMATOPAC CH=1 Report No.=7 DATA=1:@CHRM1.C00 22/07/02 16:23:48

** CALCULATION REPORT **

CH	PKNO	TIME	AREA	HEIGHT	MK	IDNO	CONC	NAME
1	1	1.4	3120779	1102222	E		29.4332	
	2	1.452	5525494	1098155	VE		52.113	
	3	1.807	1909477	318135	V		18.009	
	4	6.691	4490	1773			0.0423	
	5	15.043	205	58			0.0019	
	6	17.411	183	39			0.0017	
	7	20.439	353	48			0.0033	
	8	21.27	41933	3817			0.3955	
	TOTAL		10602911	2524247			100	

Phe56Ile catalyzed analytical scale asymmetric reduction of **11a**

4. Phe56Ile catalyzed preparative scale asymmetric reduction of **5a**

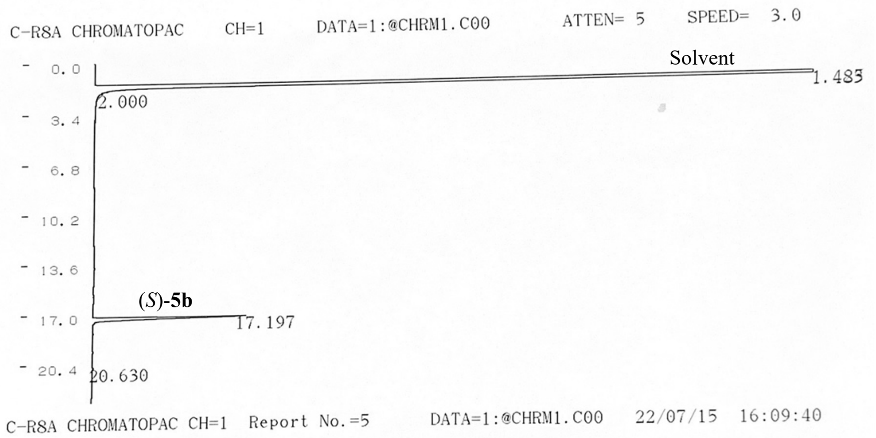

C-R8A CHROMATOPAC CH=1 Report No.=5 DATA=1:@CHRM1.C00 22/07/15 16:09:40

** CALCULATION REPORT **

CH	PKNO	TIME	AREA	HEIGHT	MK	IDNO	CONC	NAME
1	1	1.425	3448416	1102355	E		38.2063	
	2	1.483	5518339	1097615	SVE		61.1398	
	3	2	120	100	T		0.0013	
	4	17.197	58568	6976			0.6489	
	5	20.63	327	48			0.0036	
		TOTAL	9025768	2207093			100	

GC of Phe56Ile catalyzed preparative scale asymmetric reduction of **5a**

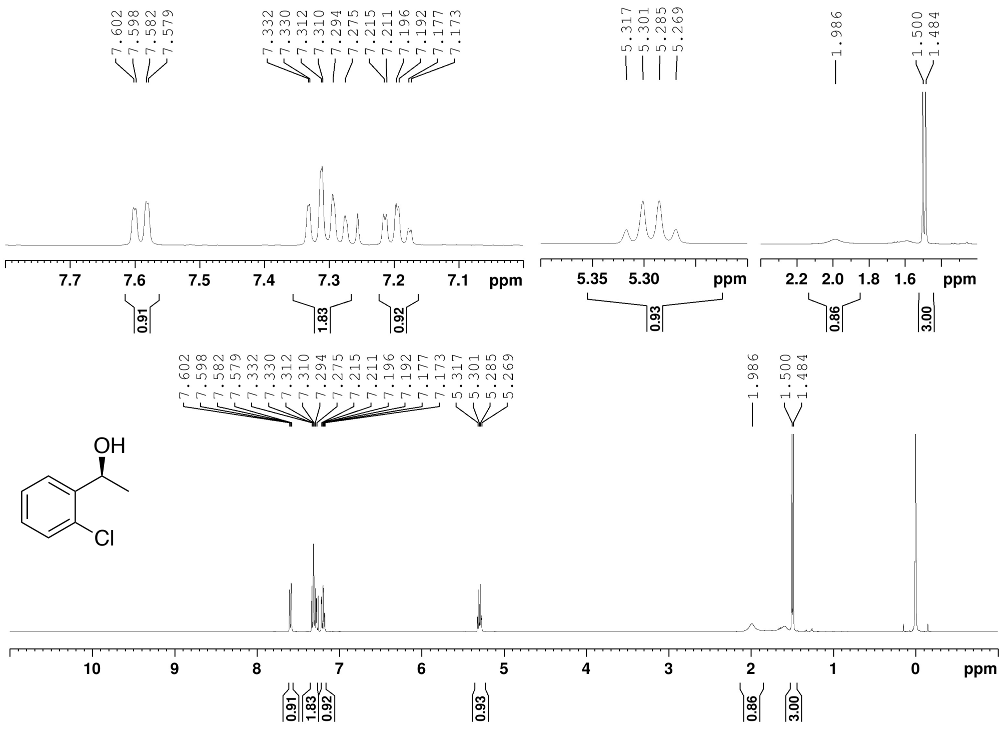

^{1}H-NMR of preparative scale synthesis of (*S*)-**5b**

5. HPLC results of racemic alcohol **12b-19b** and *Gc*APRD mutants catalyzed reduction of **12a-19a**

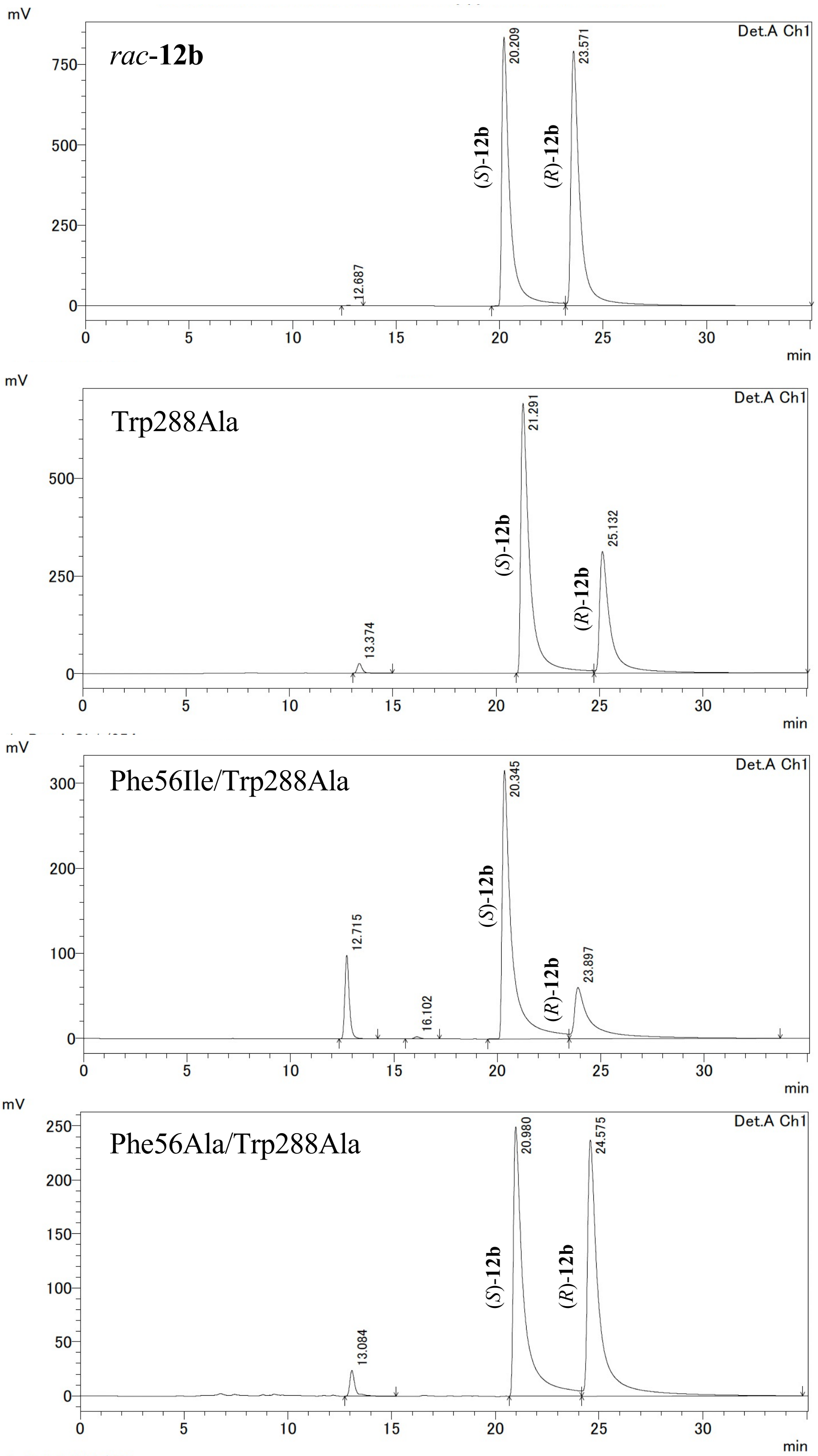

rac-**12b** and *Gc*APRD mutants catalyzed asymmetric reduction of **12a**

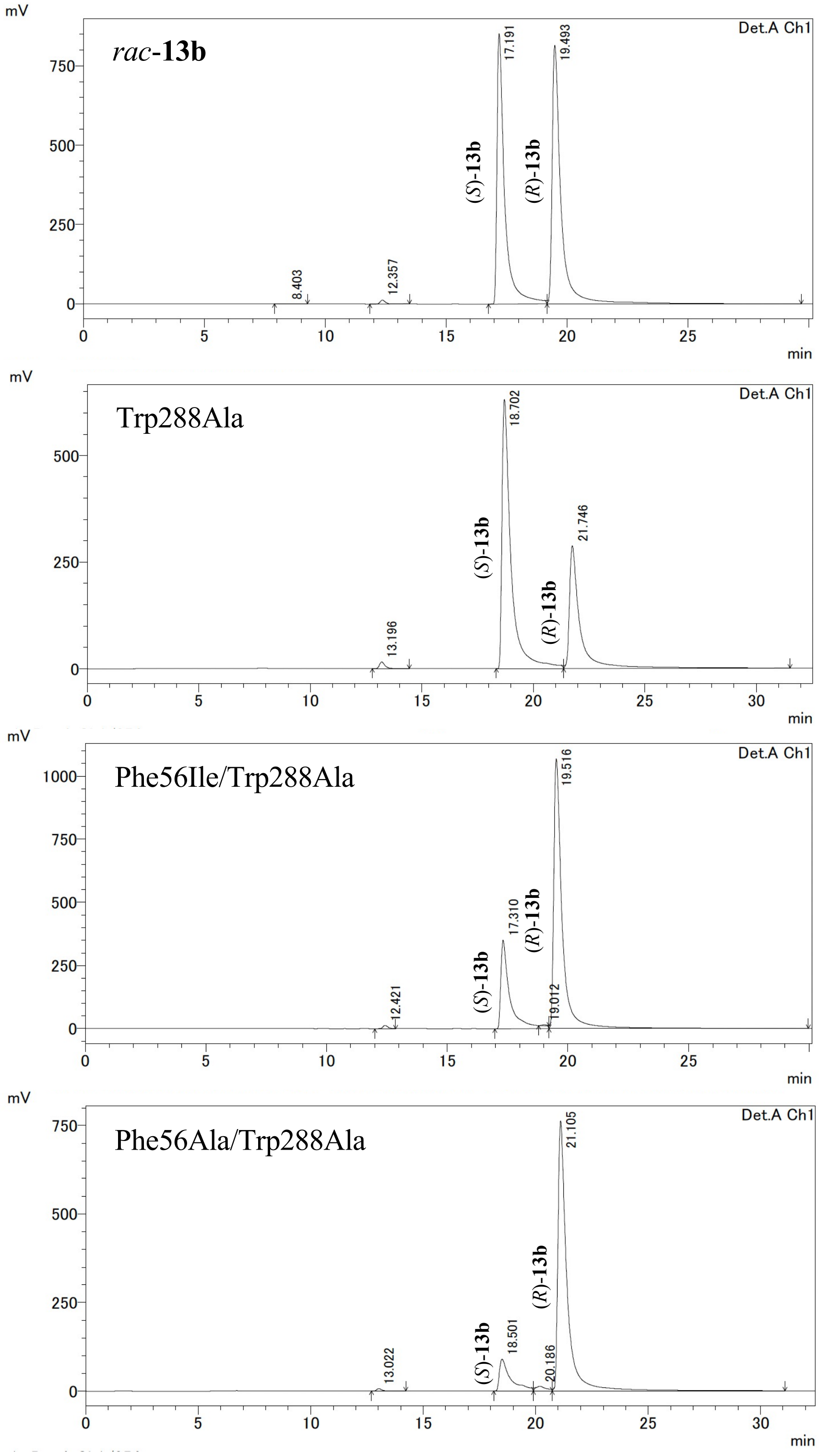

rac-**13b** and *Gc*APRD mutants catalyzed asymmetric reduction of **13a**

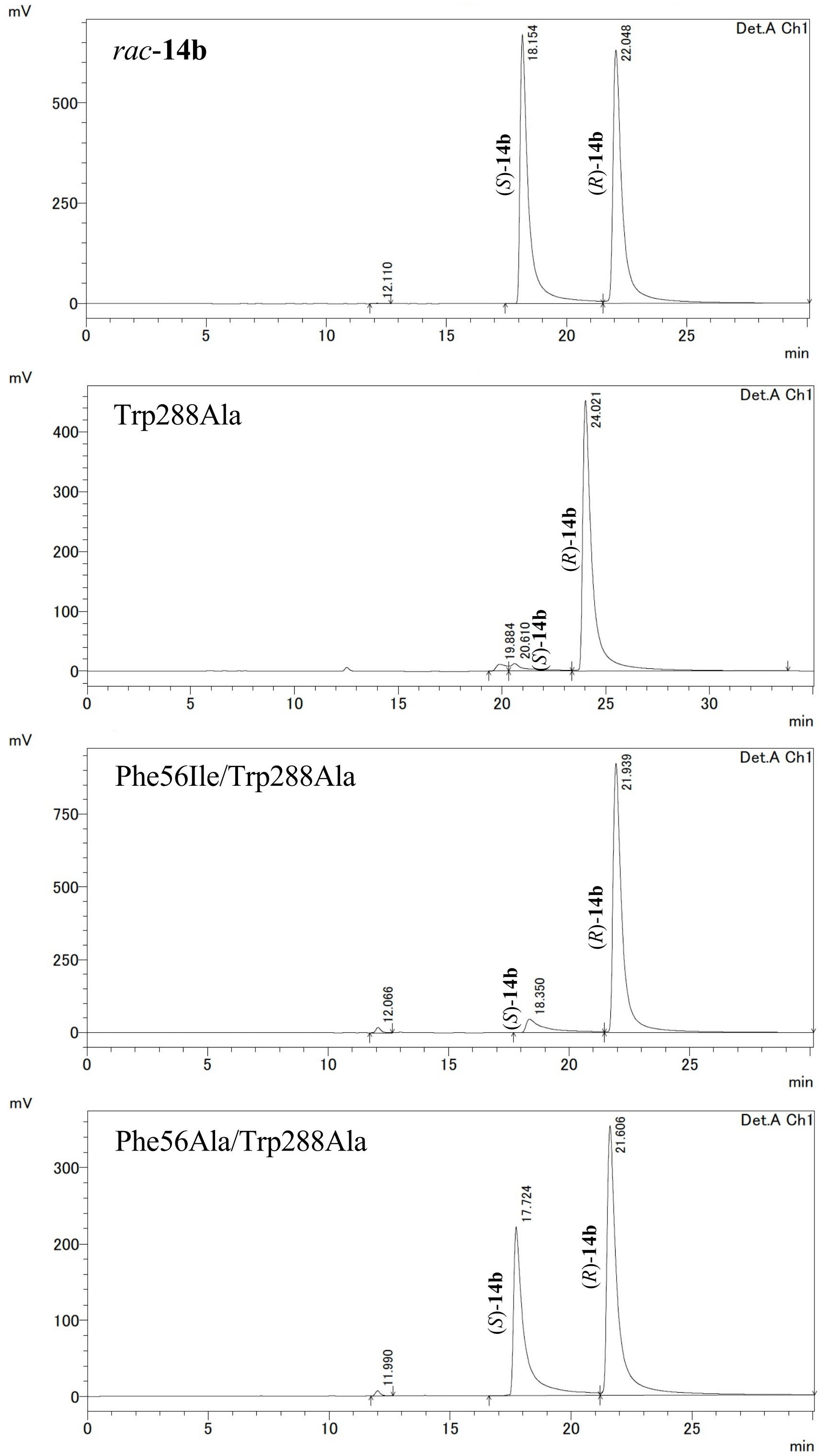

rac-**14b** and *Gc*APRD mutants catalyzed asymmetric reduction of **14a**

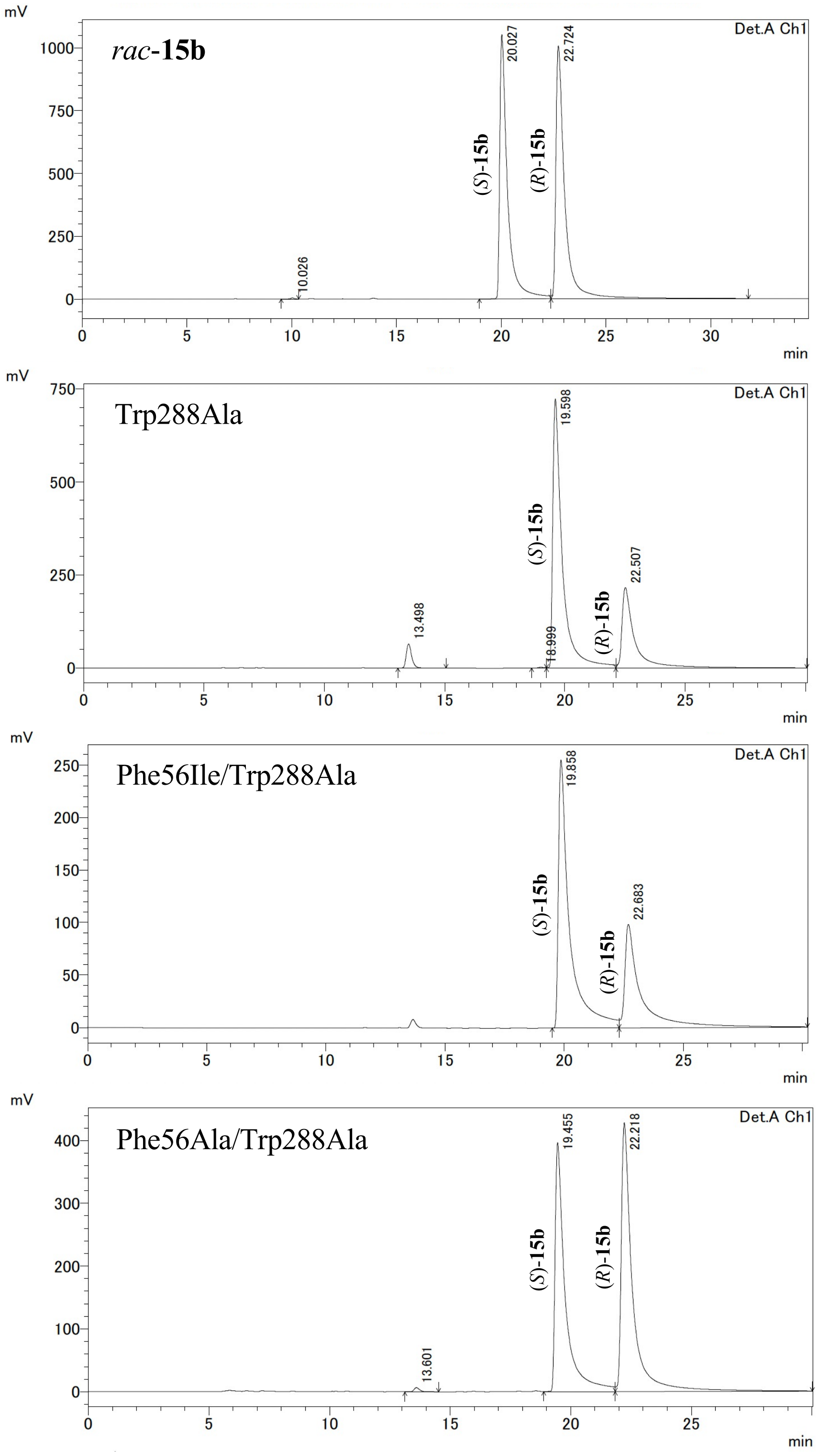

rac-**15b** and *Gc*APRD mutants catalyzed asymmetric reduction of **15a**

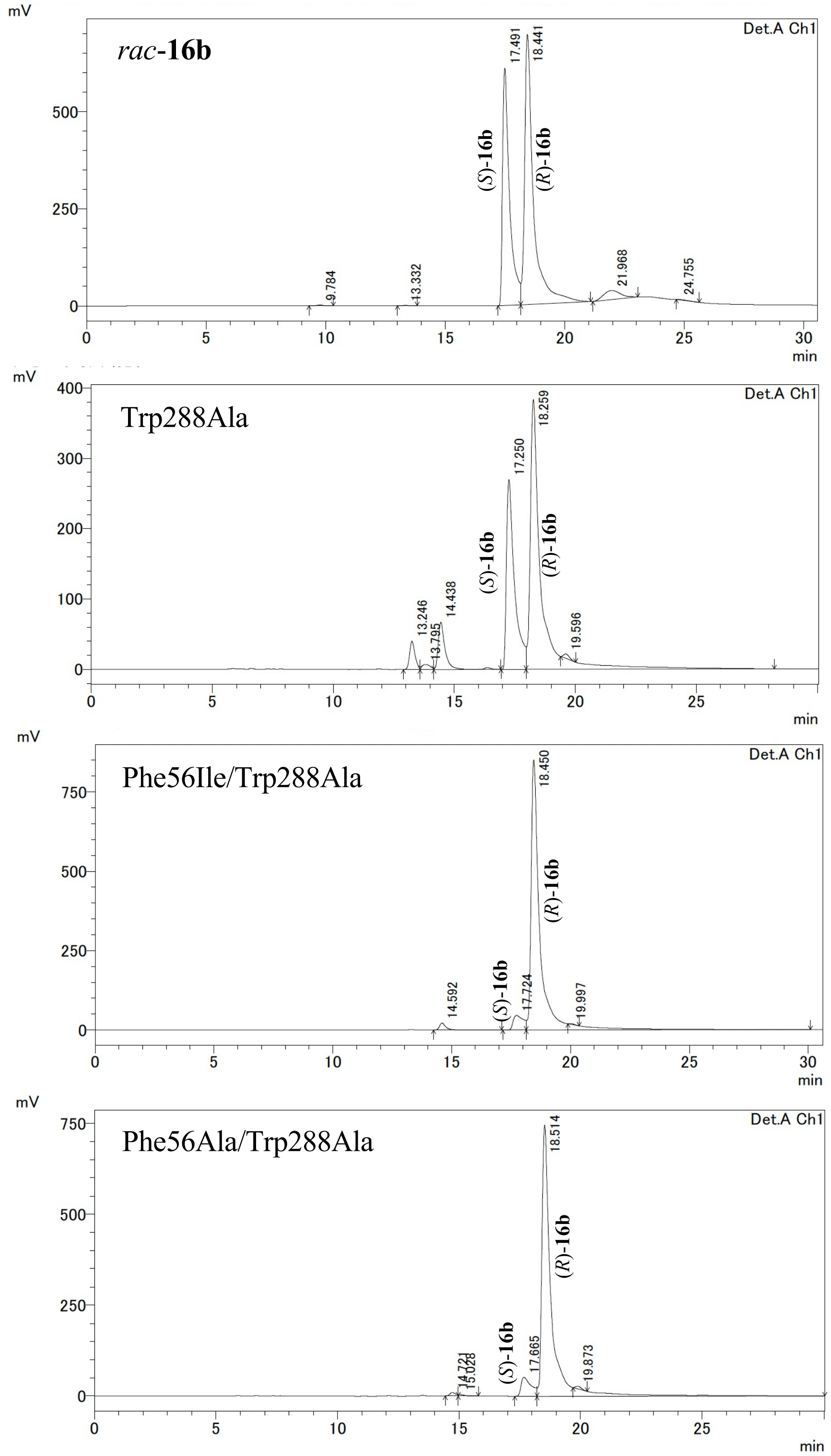

rac-**16b** and *Gc*APRD mutants catalyzed asymmetric reduction of **16a**

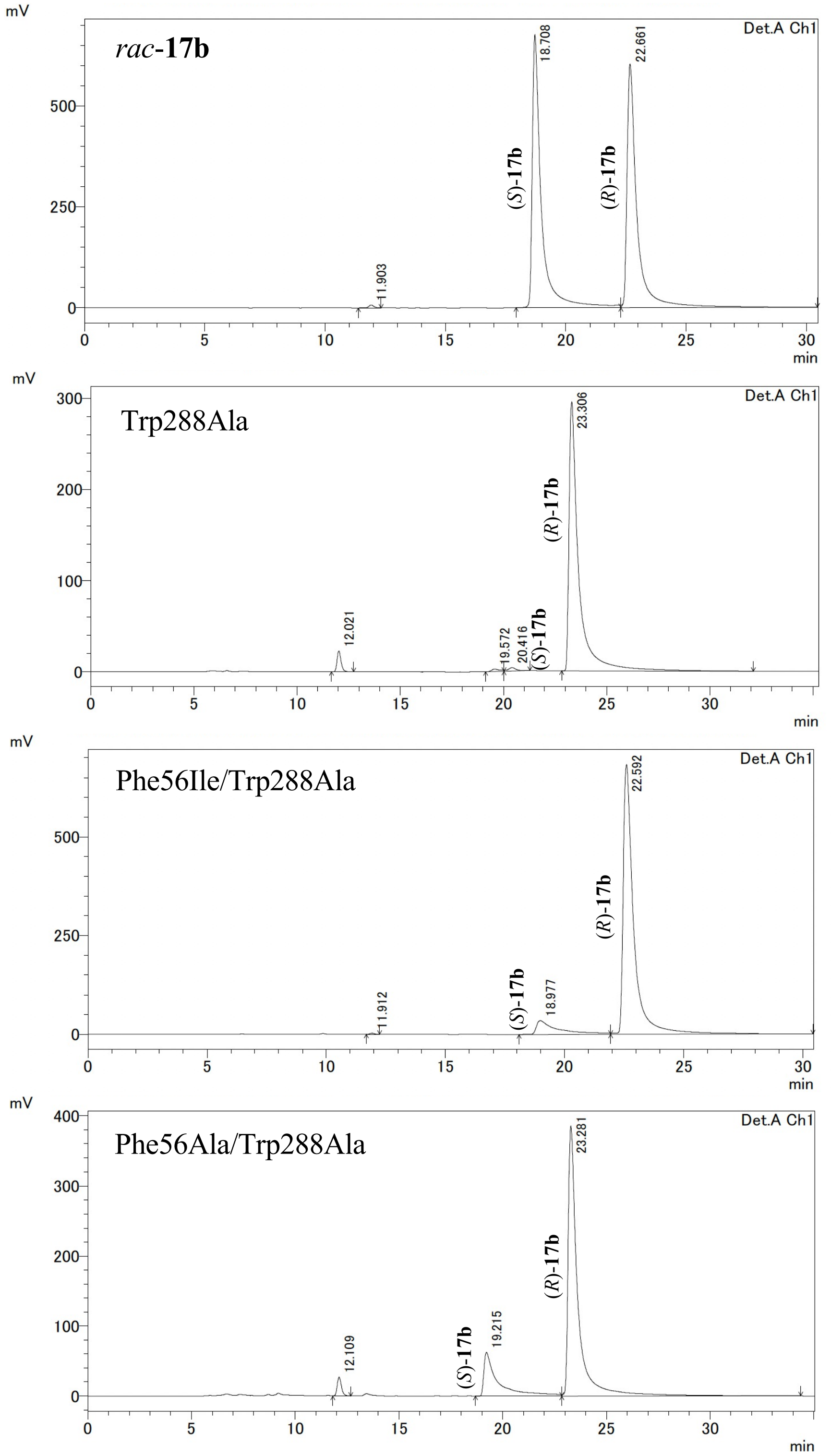

rac-**17b** and *Gc*APRD mutants catalyzed asymmetric reduction of **17a**

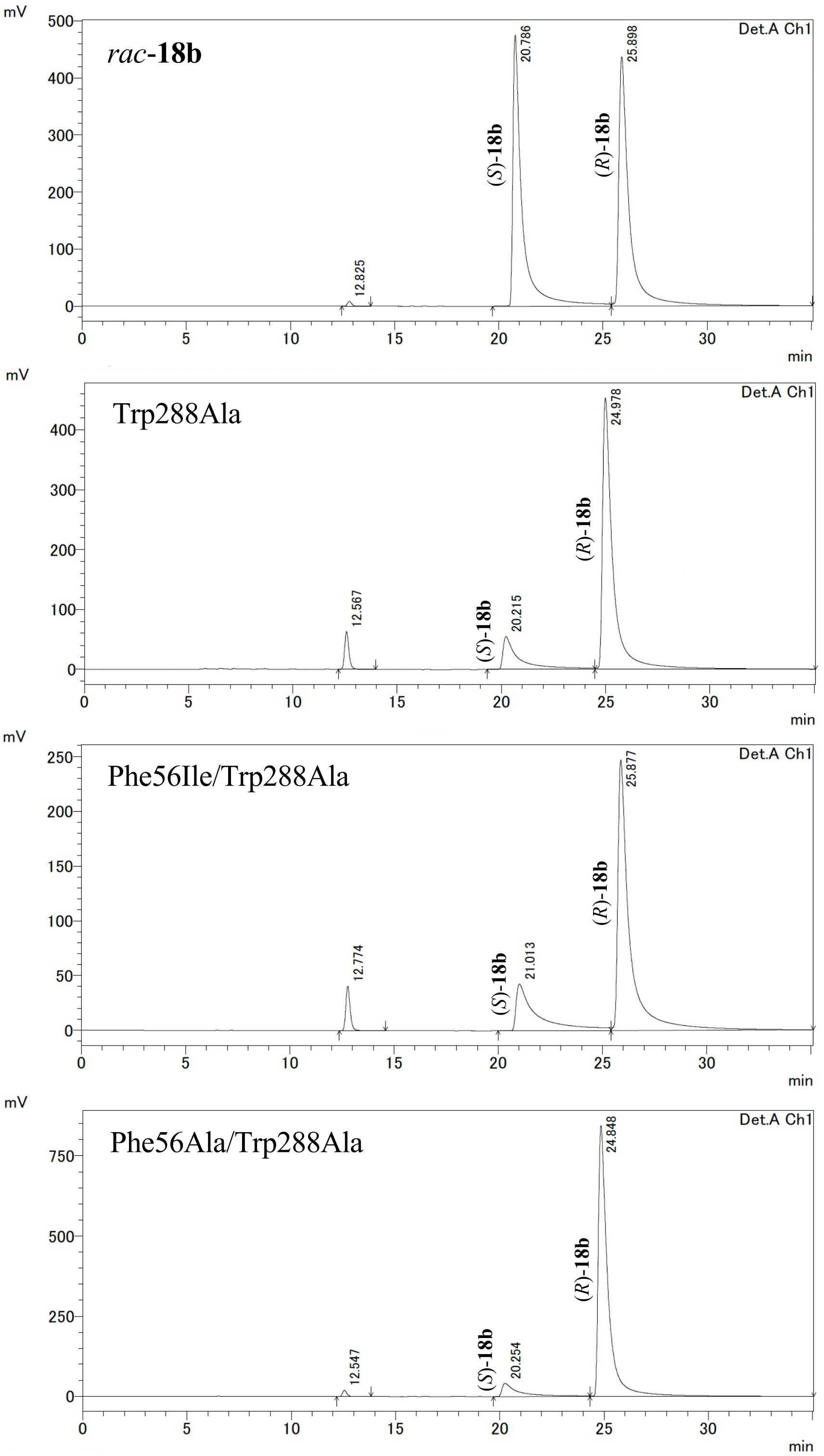

rac-**18b** and *Gc*APRD mutants catalyzed asymmetric reduction of **18a**

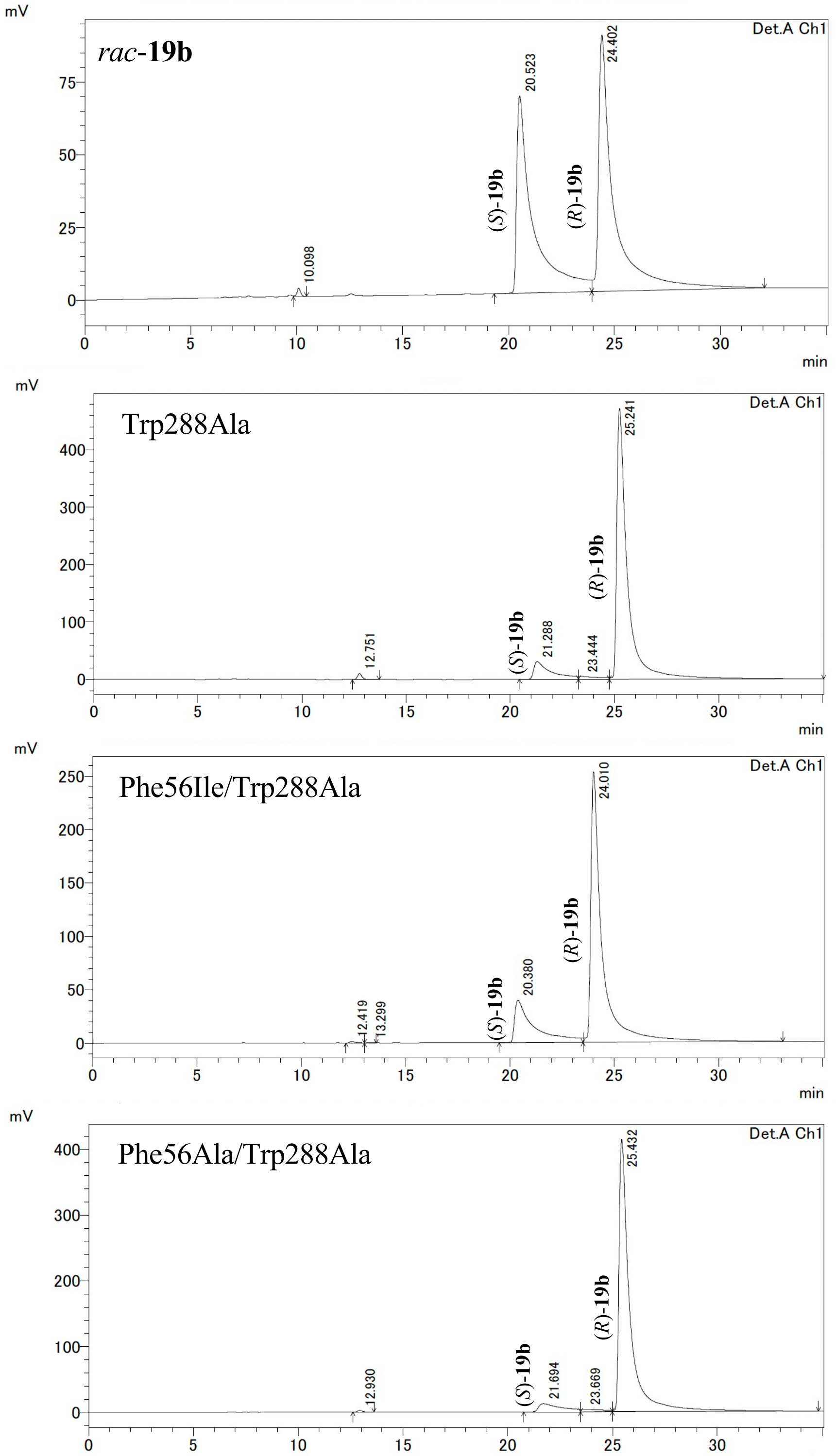

rac-**19b** and *Gc*APRD mutants catalyzed asymmetric reduction of **19a**

6. ^{1}H-NMR of the asymmetric reduction of **12a-19a** by *Gc*APRD mutants

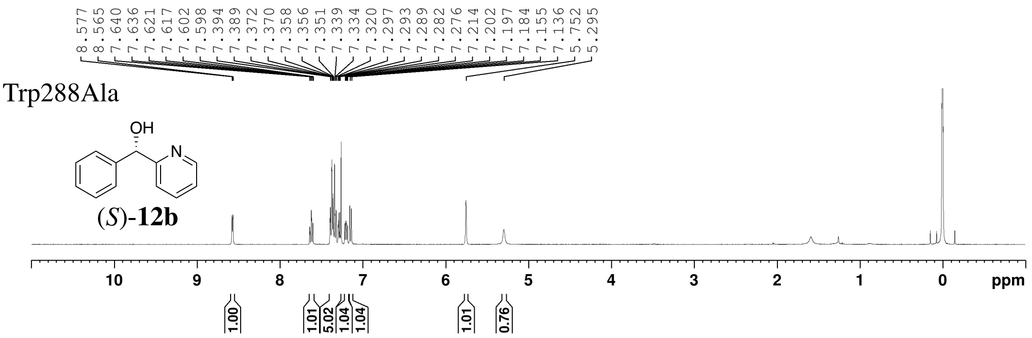

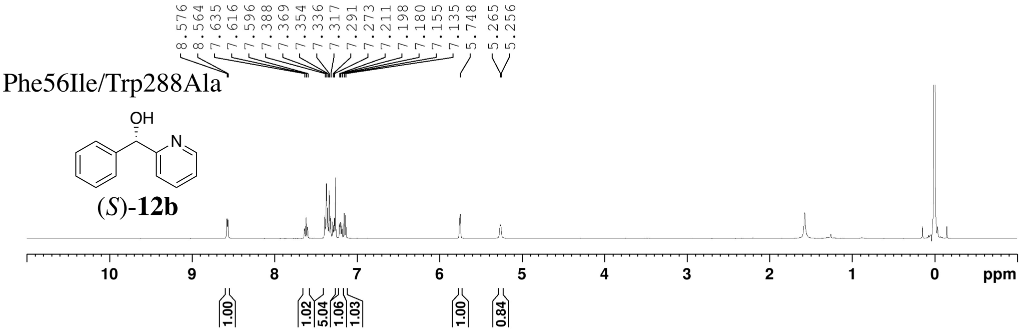

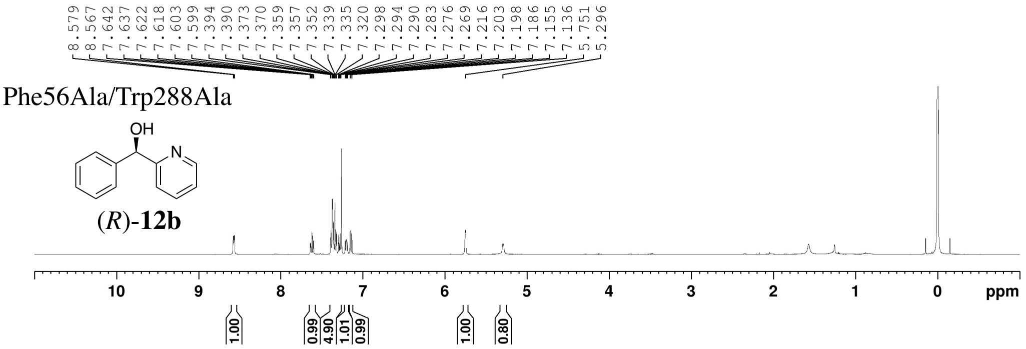

^{1}H-NMR of *Gc*APRD mutants catalyzed asymmetric reduction of **12a**

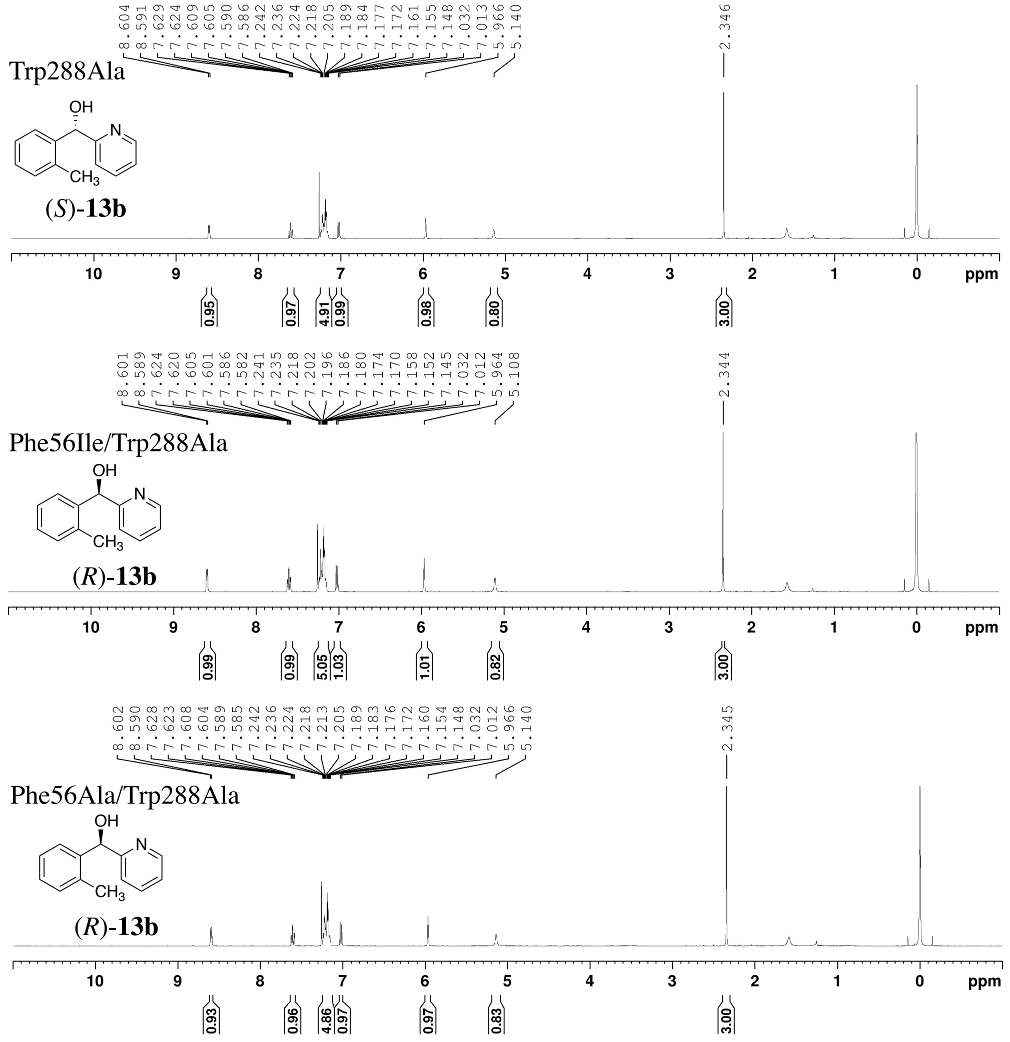

^{1}H-NMR of *Gc*APRD mutants catalyzed asymmetric reduction of **13a**

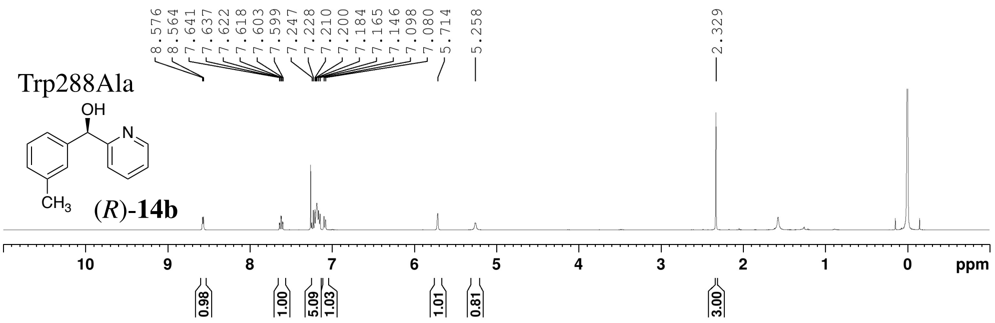

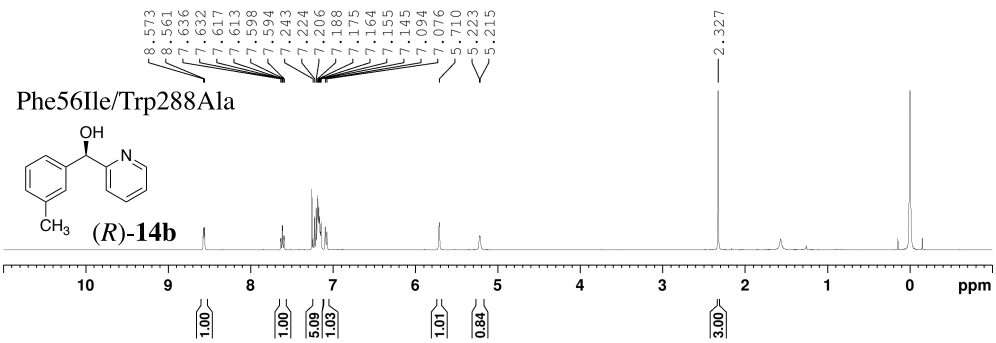

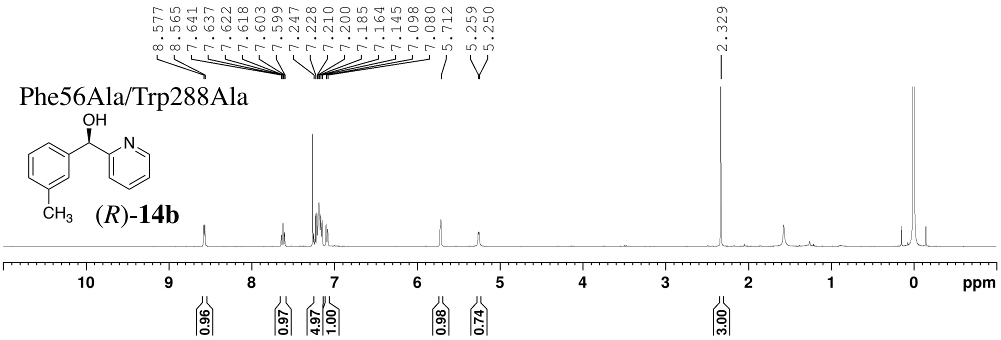

^{1}H-NMR of *Gc*APRD mutants catalyzed asymmetric reduction of **14a**

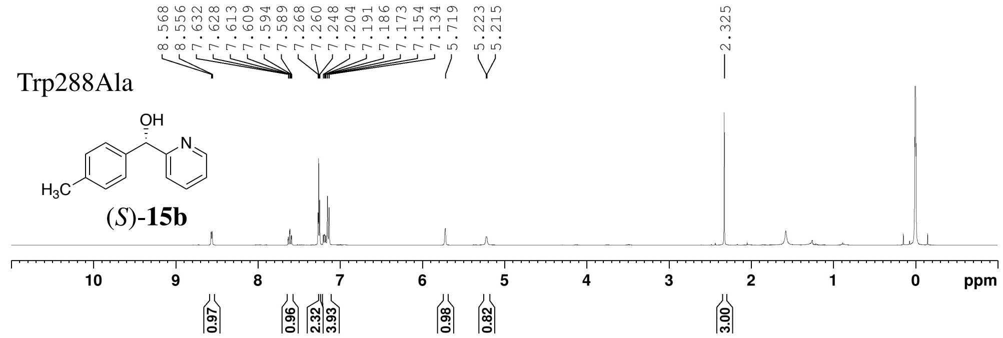

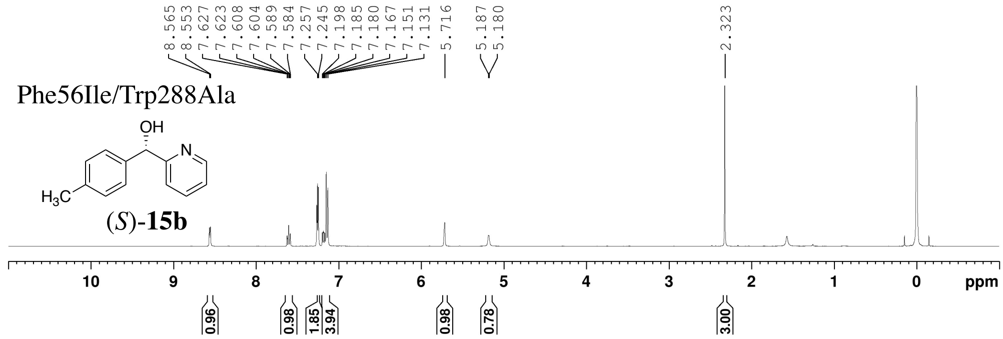

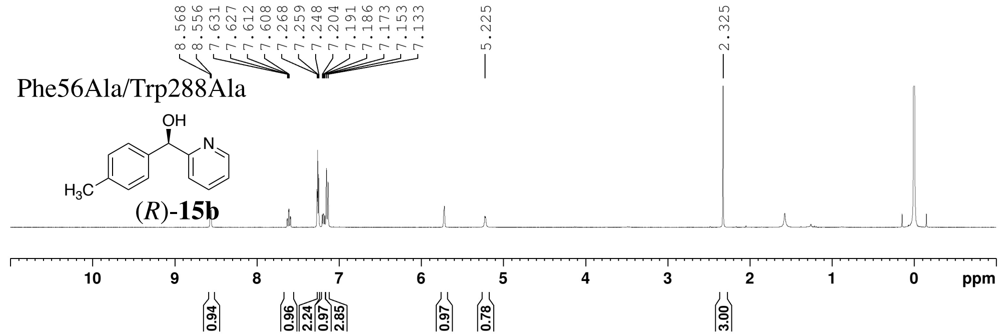

^{1}H-NMR of *Gc*APRD mutants catalyzed asymmetric reduction of **15a**

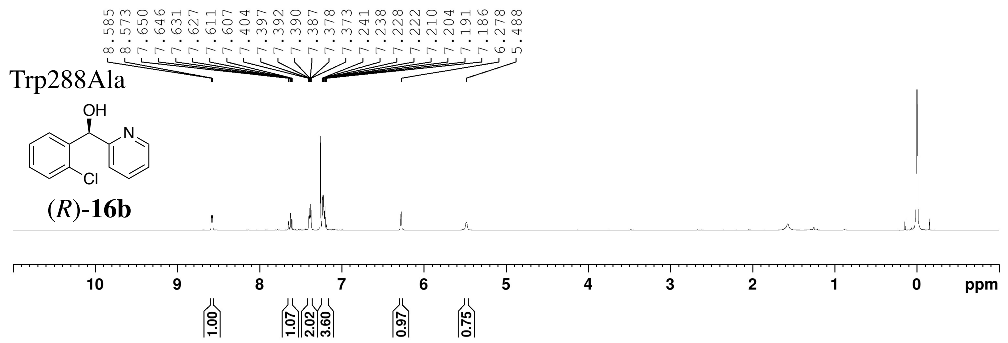

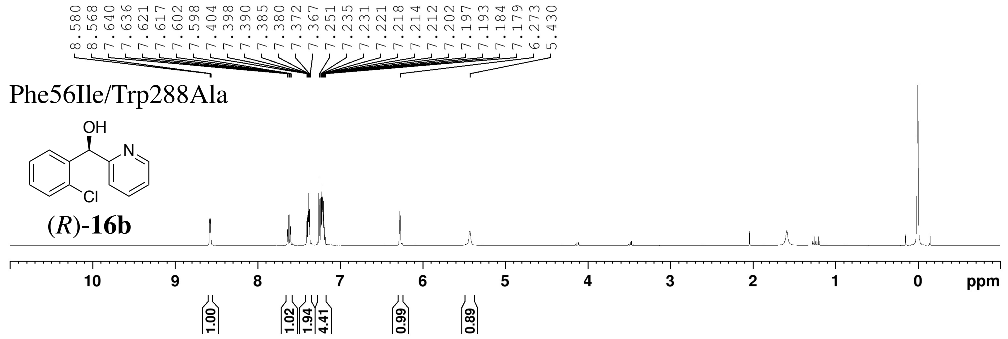

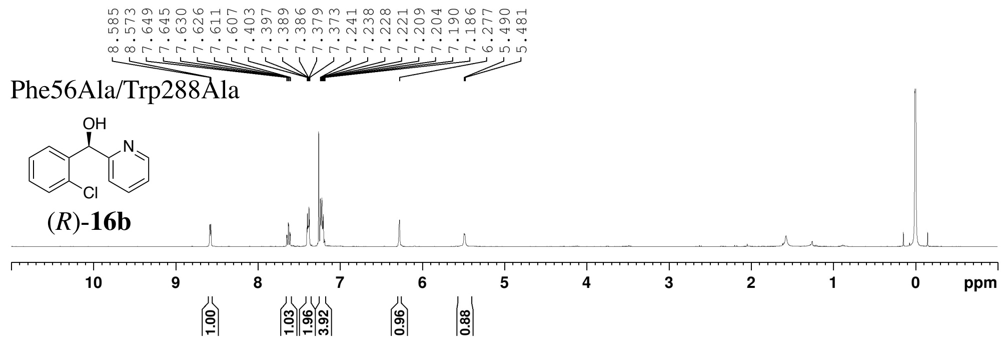

^{1}H-NMR of *Gc*APRD mutants catalyzed asymmetric reduction of **16a**

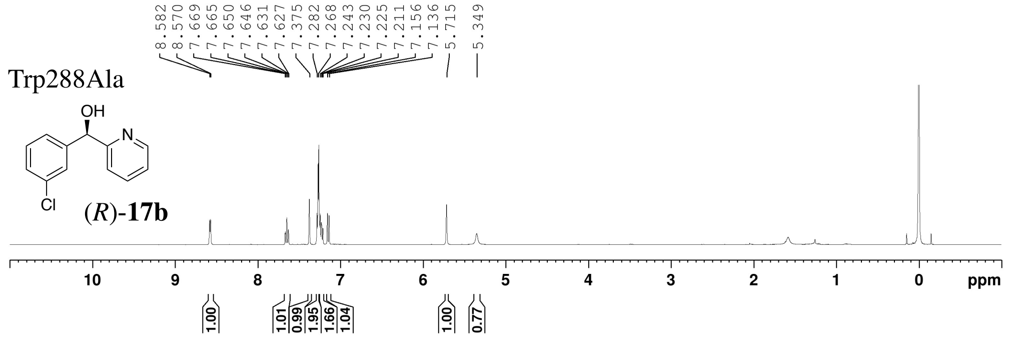

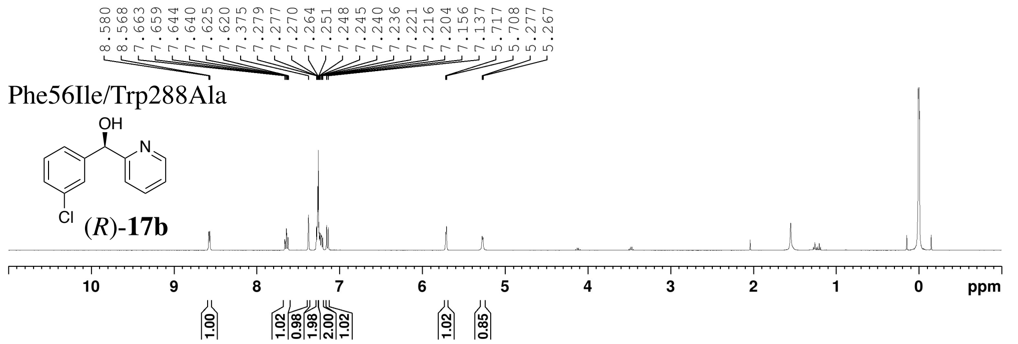

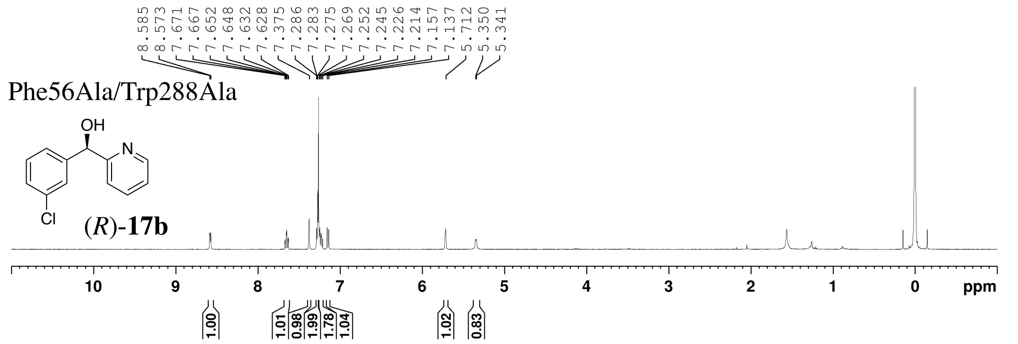

^{1}H-NMR of *Gc*APRD mutants catalyzed asymmetric reduction of **17a**

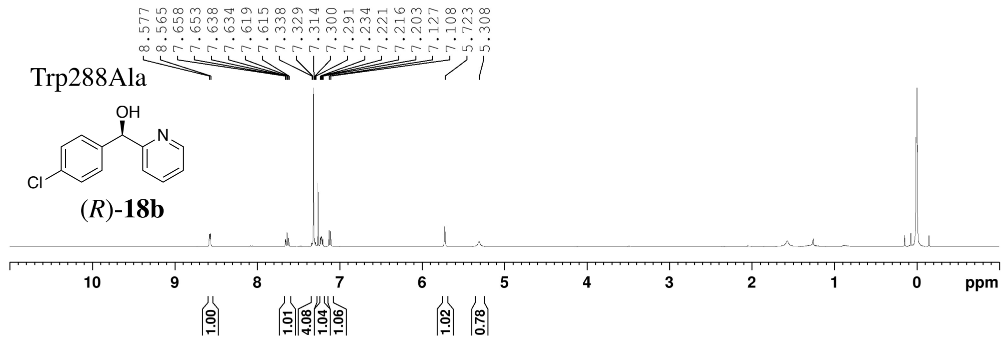

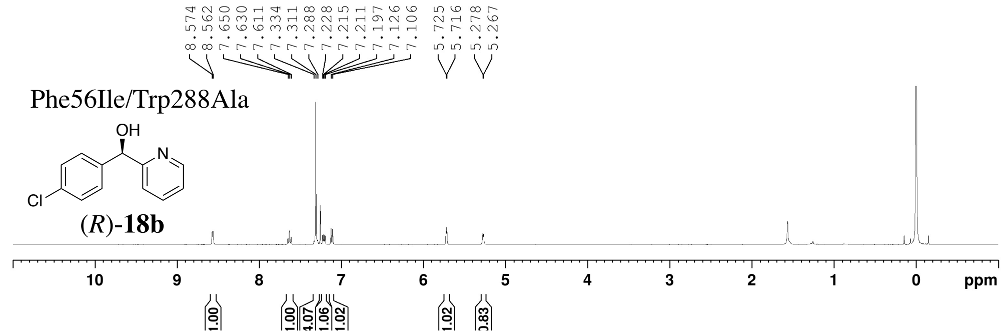

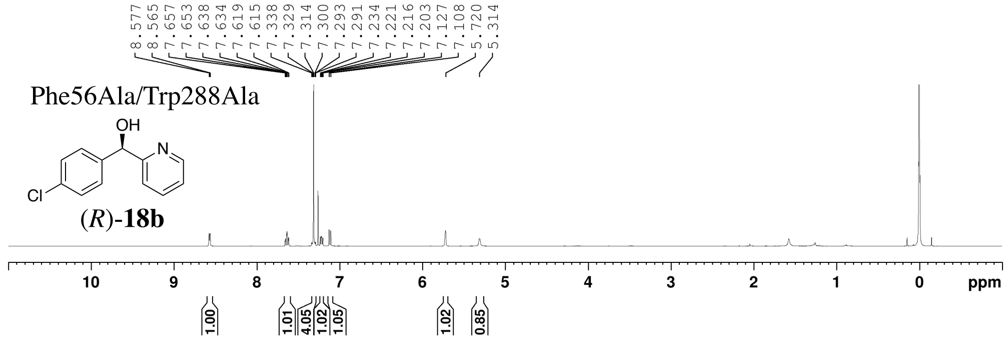

^{1}H-NMR of *Gc*APRD mutants catalyzed asymmetric reduction of **18a**

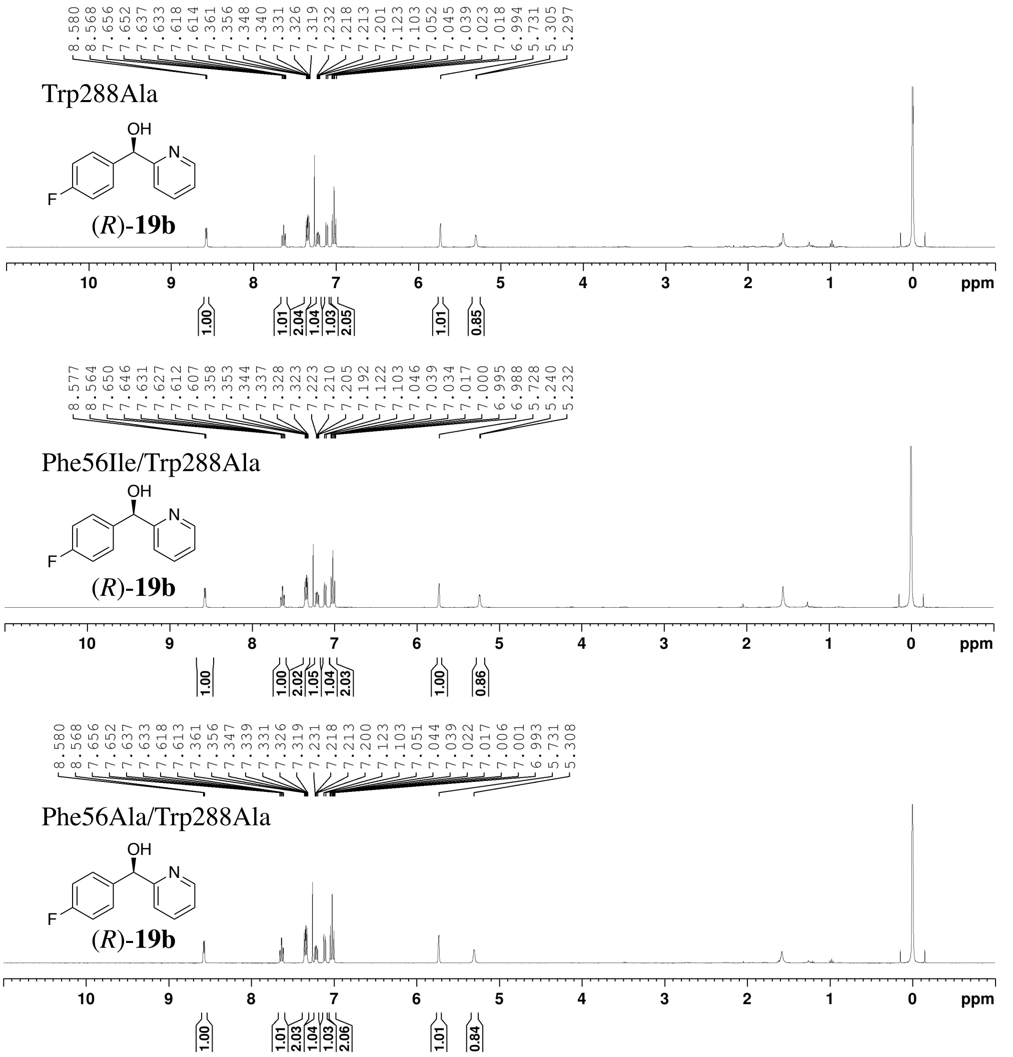

^{1}H-NMR of *Gc*APRD mutants catalyzed asymmetric reduction of **19a**
